# Supplementary material for: CRITERIA: A network decomposition and elementary flux mode translation-based tool for computing equilibria of biochemical systems
Source: PLoS Comput Biol. 2026 Aug 11;22(8):e1014633. doi: 10.1371/journal.pcbi.1014633 (PMC13475990; doi:10.1371/journal.pcbi.1014633)
Supplement: Supplementary file 1 [file pcbi.1014633.s001.pdf]

S1 Text for CRITERIA: A network decomposition and elementary flux mode translation-based tool for computing equilibria of biochemical systems

Exequiel Jun V. Villejo, Aurelio A. de los Reyes V, and Bryan S. Hernandez*
Institute of Mathematics, University of the Philippines Diliman, Quezon City, Philippines
(Dated: July 30, 2026)

SUPPLEMENTARY METHODS

Preservation of weak reversibility and zero deficiency under network merging of independent subnetworks

The newly developed framework for computing the equilibria of biochemical reaction networks consists of the following ordered steps: network decomposition, network translation, merging of the translated subnetworks through the union of their respective reaction sets, and lastly, the computation of the equilibria of the merged network. The EFM–based network translation, when successful, produces a WR and DZ translation of the network. Consequently, if the method yields a valid translation, then after the second step of the framework we obtain subnetworks that are WR and DZ. This naturally raises the question of whether merging the translated subnetworks preserves the properties of WR and DZ. If so, we can always apply the deficiency-based approach of Johnston, Müller, and Pantea in computing the equilibria of biochemical reaction systems which is given by Theorem 2 in the main text [1].

Theorem S1. *Let $\mathcal{N}$ be a chemical reaction network (CRN) with α independent subnetworks $\mathcal{N}_1, \mathcal{N}_2, \ldots, \mathcal{N}_\alpha$. Suppose that for each $i = 1, 2, \ldots, \alpha$, there exists a weakly reversible and deficiency zero translation $\widetilde{\mathcal{N}}_i$ for $\mathcal{N}_i$. Then the network $\mathcal{N}' = \bigcup_{i=1}^{\alpha} \widetilde{\mathcal{N}}_i$ admits a generalized CRN such that its stoichiometric CRN $\mathcal{N}'_S$ is weakly reversible and deficiency zero, and its kinetic-order CRN $\mathcal{N}'_K$ is weakly reversible.*

Proof. Let $\mathcal{N}$ be a CRN that is decomposed into α independent subnetworks $\mathcal{N}_1, \mathcal{N}_2, \ldots, \mathcal{N}_\alpha$. Suppose each $\mathcal{N}_i$ admits a weakly reversible and deficiency zero structural translation $\widetilde{\mathcal{N}}_i$ for $i = 1, 2, \ldots, \alpha$. So, we have $\mathcal{N}'_S = \bigcup_{i=1}^{\alpha} \widetilde{\mathcal{N}}_i$. We first show that $\mathcal{N}'_S$ is weakly reversible. We consider the following cases:

- Case 1: $\widetilde{\mathcal{C}}_{S_i} \cap \widetilde{\mathcal{C}}_{S_j} = \emptyset$ $(i \neq j)$, where $\widetilde{\mathcal{C}}_{S_i}$ is the translated complex set of subnetwork $\mathcal{N}_i$.

  Then no reaction in $\widetilde{\mathcal{N}}_i$ shares a complex with any reaction in $\widetilde{\mathcal{N}}_j$, $i \neq j$. So, $\mathcal{N}'_S$ is the disjoint union of the reaction graphs of the subnetworks $\widetilde{\mathcal{N}}_i$, for $i = 1, 2, \ldots, \alpha$. Let $\widetilde{\Lambda}_{S_i}$ be a linkage class of $\mathcal{N}'_S$. Since $\mathcal{N}'_S = \bigsqcup_{i=1}^{\alpha} \widetilde{\mathcal{N}}_i$, $\widetilde{\Lambda}_{S_i}$ is contained in some subnetwork $\widetilde{\mathcal{N}}_i$. By hypothesis, every linkage class of $\widetilde{\mathcal{N}}_i$ is a strong linkage class for all $i = 1, 2, \ldots, \alpha$. Hence, $\widetilde{\Lambda}_{S_i}$ is a strong linkage class implying that $\mathcal{N}'_S$ is weakly reversible.

- Case 2: $\widetilde{\mathcal{C}}_{S_i} \cap \widetilde{\mathcal{C}}_{S_j} \neq \emptyset$ $(i \neq j)$

  Let $\widetilde{\Lambda}_{S_i}$ be a linkage class of $\mathcal{N}'_S$. Then $\widetilde{\Lambda}_{S_i}$ can be written as $\widetilde{\Lambda}_{S_i} = \bigcup_{k=1}^{p} \widetilde{\Lambda}_{i_k}$, where p is the number of linkage classes with common complexes, and $\widetilde{\Lambda}_{i_k}$ is a linkage classes of $\widetilde{\mathcal{N}}_i$. Since $\widetilde{\mathcal{N}}_i$ is weakly reversible for all $i = 1, 2, \ldots, \alpha$, then $\widetilde{\Lambda}_{i_k}$ is a strong linkage class for all $i = 1, 2, \ldots, \alpha$ and $k = 1, 2, \ldots, p$.

  Let y, y' be complexes in $\widetilde{\mathcal{N}}_i$. If $y, y' \in \widetilde{\Lambda}_{i_k}$ for some k, then there exists a directed path from y to y', and vice versa, by hypothesis. Suppose $y \in \widetilde{\Lambda}_{i_j}$ and $y' \in \widetilde{\Lambda}_{i_l}$ for some $j \neq l$. Since $\widetilde{\Lambda}_{S_i} = \bigcup_{k=1}^{p} \widetilde{\Lambda}_{i_k}$, there exists a series of complexes $y_0, y_1, \ldots, y_m$ where $y_k \in \widetilde{\Lambda}_{i_{j+k}} \cap \widetilde{\Lambda}_{i_{j+k+1}}$ for $k = 0, 1, 2, \ldots, m$ so that

$$\widetilde{\Lambda}_{i_j} \to y_0 \to \widetilde{\Lambda}_{i_{j+1}} \to y_1 \to \cdots \to y_m \to \widetilde{\Lambda}_{i_{j+k+m}} =: \widetilde{\Lambda}_{i_l}$$

* bshernandez@up.edu.ph

Since $\widetilde{\Lambda}_{i_k}$ is weakly reversible for all k, we can find a directed path from y to y', and vice versa. Hence, $\widetilde{\Lambda}_{S_i}$ is a strong linkage class. Since i is arbitrary, then $\mathcal{N}'_S$ is weakly reversible.

Now, we show that the kinetic-order CRN $\mathcal{N}'_K$ is also weakly reversible. Let $\mathcal{C}_K$ denote the set of kinetic complexes. Define the mapping

$$\pi : \mathcal{C}_K \to \widetilde{\mathcal{C}}_S$$

which maps each kinetic complex of $\mathcal{N}'_K$ to its associated stoichiometric complex. Moreover, define $F_y = \pi^{-1}(y)$, for $y \in \widetilde{\mathcal{C}}_S$, which is the set of kinetic complexes with the same associated stoichiometric complex.

Let $\widetilde{y} \to \widetilde{y}'$ be a reaction in $\mathcal{N}'_K$. Then $\pi(\widetilde{y}) \to \pi(\widetilde{y}')$ is a reaction in $\mathcal{N}'_S$. Since we have shown that $\mathcal{N}'_S$ is weakly reversible, there exists a directed path $\pi(\widetilde{y}') = \bar{y}_0 \to \bar{y}_1 \to \cdots \to \bar{y}_r = \pi(\widetilde{y})$. Here, each reaction $\bar{y}_i \to \bar{y}_{i+1}$ corresponds to a reaction in $\mathcal{N}'_K$. Hence, we have $\widetilde{y}' \to \widetilde{y}_1 \to \widetilde{y}_2 \to \cdots \to \widetilde{y}_r$ in $\mathcal{N}'_K$ with $\pi(\widetilde{y}_r) = \pi(\widetilde{y})$.

If $\widetilde{y}_r = \widetilde{y}$, we are done. Otherwise, $\widetilde{y}_r, \widetilde{y} \in F_{\pi(\widetilde{y})}$. By construction of phantom edges, there must exist a directed path $\widetilde{y}_k \rightsquigarrow \widetilde{y}$, and vice versa. Concatenating paths yields $\widetilde{y}' \rightsquigarrow \widetilde{y}$. Hence every reaction is contained in a directed cycle. Therefore, $\mathcal{N}'_K$ is weakly reversible.

Lastly, we show that the effective deficiency δ' (i.e., deficiency of $\mathcal{N}'_S$) is equal to 0. Similar to what we did above, we consider the following cases:

- Case 1: $\widetilde{\mathcal{C}}_{S_i} \cap \widetilde{\mathcal{C}}_{S_j} = \emptyset$ $(i \neq j)$

  Since the decomposition of $\mathcal{N}$ is independent, we have $\delta' \leq \sum_{i=1}^{\alpha} \delta'_i$ [2], where δ'_i is the deficiency of the translated subnetwork $\widetilde{\mathcal{N}}_i$. By hypothesis, $\delta'_i = 0$ for all $i = 1, 2, \ldots, \alpha$. Since the deficiency of a network is a nonnegative integer, it follows that $\delta' = 0$.

- Case 2: $\widetilde{\mathcal{C}}_{S_i} \cap \widetilde{\mathcal{C}}_{S_j} \neq \emptyset$ $(i \neq j)$

  Let $y \in \widetilde{\Lambda}_{S_i}$, where $i \in I = \{1, 2, \ldots, p_y\}$. To eliminate common complexes, we need $p_y - 1$ merging of linkage classes. Denote $k := \sum_y (p_y - 1)$. Let $\widetilde{\mathcal{N}}_i = \left(\widetilde{\mathcal{S}}_{S_i}, \widetilde{\mathcal{C}}_{S_i}, \widetilde{\mathcal{R}}_{S_i}\right)$ be the translated i-th subnetwork. We consider the reaction graph $G^{(0)} = \left(V^{(0)}, E^{(0)}\right)$, where $V^{(0)} = \bigsqcup_{i=1}^{\alpha} \widetilde{\mathcal{C}}_{S_i}$ and $E^{(0)} = \bigsqcup_{i=1}^{\alpha} \widetilde{\mathcal{R}}_{S_i}$. We construct a sequence of reaction graphs $G^{(j)}$, for $j = 1, 2, \ldots, k$, by successively merging linkage classes with common complexes. Let $\ell^{(j)}$ be the number of linkage classes of $G^{(j)}$. At step j, we merge $\widetilde{\Lambda}_{S_i}$ and $\widetilde{\Lambda}_{S_j}$ via y where $i \neq j$ and $i, j \in I$. So, $\ell^{(j+1)} = \ell^{(j)} - 1$ which implies that $\ell^{(j)} - \ell^{(j+1)} = 1$. Summing over $j = 0, 1, 2, \ldots, k-1$, we have

  $$\sum_{j=0}^{k-1} \left(\ell^{(j)} - \ell^{(j+1)}\right) = \sum_{j=0}^{k-1} 1$$
  $$m := \ell^{(0)} - \ell^{(k)} = k$$

  Now, we know that $\delta'_i = n'_i - l'_i - s'_i = 0$ for all i by hypothesis. So,

  $$\begin{aligned} \delta' &= n' - l' - s' \\ &= \sum_i (n'_i - k) - \sum_i (l'_i - m) - \sum_i s'_i \\ &= \sum_i (n'_i - l'_i - s'_i) - k + m \\ &= 0 \end{aligned}$$

In both cases, we have shown that $\delta' = 0$. Hence, $\mathcal{N}'_S$ is deficiency zero. □

We note that the phantom edges in the kinetic-order CRN can be constructed in such a way that the resulting reaction network is WR. This is possible because phantom edges do not affect the dynamics of the system. Each phantom edge is assigned a free parameter as its "reaction rate," and thus serves only as a bookkeeping device used to define the graph of the kinetic-order CRN.

By Theorem 2 in the main text, there is no restriction on the kinetic deficiency of the network. When the kinetic deficiency is positive, certain additional conditions are required in order to parametrize the equilibria. Consequently, in Theorem S1, it is not necessary to impose the condition that the kinetic-order CRN is DZ.

Detailed computation of the EFMs

In this subsection, we discuss in detail the computation of EFMs, which constitutes the first step of the newly adopted network translation method. In Step 4b of Algorithm 1 in the main text, a test is performed to determine whether the newly computed vector $r \in \mathbb{R}^r_{\geq 0}$ is an admissible flux vector. We note that if $r \neq 0$, then it is a ray of the polyhedral cone P. Since we need the extreme rays of P, there is a need to check for extremeness. There are several tests for this purpose, but Algorithm 1 performs the Rank Test [3].

For any ray r in a polyhedral cone $P(A)$, its *zero set* is defined to be the index zet $Z(r) = \{i |\, A_{i:}r = 0\}$. In other words, these are the indices of the row vectors of A that are satisfied by r with equality. Moreover, the *zero-set matrix* for r, denoted by $A_{Z(r)}$, is the submatrix of A composed of only those rows of A indexed by $Z(r)$ [3]. Given these definitions, the Rank Test states that if $\text{rank}(A_{Z(r)}) = r-1$, then r is an extreme ray of $P(A)$. (Note that the r in the RHS of the equation is the number of reactions in the network.) Since polyhedral cones can be thought of as being determined by hyperplanes defined from the rows of the representative matrix A, then the extreme rays are the edges of the polyhedral cone which are the vectors that satisfy as many constraints (i.e., $A_{i:}r = 0$) as possible without being forced to be zero. If a ray is an intersection of fewer than $r-1$ constraints, then it can be expressed as a nonnegative linear combination of other rays. Thus, it is not an edge of the polyhedral cone and therefore not an extreme ray.

We now provide the step-by-step procedure in computing the EFMs of the histidine kinase system in the main text. Shown below is the corresponding reaction network of the system and its stoichiometric matrix N for easier reference.

$$X \xrightarrow{k_1} X_p$$

$$X_p + Y \underset{k_3}{\overset{k_2}{\rightleftarrows}} X + Y_p \qquad N = \begin{bmatrix} -1 & 1 & -1 & 0 \\ 1 & -1 & 1 & 0 \\ 0 & -1 & 1 & 1 \\ 0 & 1 & -1 & -1 \end{bmatrix}$$

$$Y_p \xrightarrow{k_4} Y$$

We construct the representative matrix A to be

$$A = \begin{bmatrix} I_r \\ N \\ -N \end{bmatrix} = \left[\begin{array}{cccc|cccc|cccc} 1 & 0 & 0 & 0 & -1 & 1 & 0 & 0 & 1 & -1 & 0 & 0 \\ 0 & 1 & 0 & 0 & 1 & -1 & -1 & 1 & -1 & 1 & 1 & -1 \\ 0 & 0 & 1 & 0 & -1 & 1 & 1 & -1 & 1 & -1 & -1 & 1 \\ 0 & 0 & 0 & 1 & 0 & 0 & 1 & -1 & 0 & 0 & -1 & 1 \end{array}\right]^T$$

where vertical lines are included to separate submatrix components.

Iteration 1. Since the network has 4 reactions, we initialize the algorithm with $k = r + 2 = 4 + 2 = 6$ and

$$A_{\text{old}} = \begin{bmatrix} 1 & 0 & 0 & 0 \\ 0 & 1 & 0 & 0 \\ 0 & 0 & 1 & 0 \\ 0 & 0 & 0 & 1 \end{bmatrix} \qquad R_{\text{old}} = \begin{bmatrix} 1 & 0 & 0 & 0 \\ 0 & 1 & 0 & 0 \\ 0 & 0 & 1 & 0 \\ 0 & 0 & 0 & 1 \end{bmatrix}.$$

For the next step, we choose $v := N_{1:} = [-1 \;\; 1 \;\; -1 \;\; 0]$. So,

$$A_{\text{new}} = \left[\begin{array}{cccc|c|c} 1 & 0 & 0 & 0 & -1 & 1 \\ 0 & 1 & 0 & 0 & 1 & -1 \\ 0 & 0 & 1 & 0 & -1 & 1 \\ 0 & 0 & 0 & 1 & 0 & 0 \end{array}\right]^T.$$

Currently, there are 4 columns of R_{old}. So, we have the index set $\Lambda = \{1, 2, 3, 4\}$. Multiplying v with the columns of R_{old} gives us the following scalars:

$$\begin{aligned} v \cdot R_{\text{old}}(:,1) &= [-1 \;\; 1 \;\; -1 \;\; 0] \cdot [1 \;\; 0 \;\; 0 \;\; 0]^T = -1 \\ v \cdot R_{\text{old}}(:,2) &= [-1 \;\; 1 \;\; -1 \;\; 0] \cdot [0 \;\; 1 \;\; 0 \;\; 0]^T = 1 \\ v \cdot R_{\text{old}}(:,3) &= [-1 \;\; 1 \;\; -1 \;\; 0] \cdot [0 \;\; 0 \;\; 1 \;\; 0]^T = -1 \\ v \cdot R_{\text{old}}(:,4) &= [-1 \;\; 1 \;\; -1 \;\; 0] \cdot [0 \;\; 0 \;\; 0 \;\; 1]^T = 0 \end{aligned}$$

This results in the following partition of the index set Λ:

$$\Lambda_+ = \{2\} \qquad \Lambda_0 = \{4\} \qquad \Lambda_- = \{1,3\}$$

There are two possible combinations from $\Lambda_+ \times \Lambda_-$ which are r_{21} and r_{23}. So,

$$\begin{aligned} r_{21} &= (v \cdot R_{\text{old}}(:,2))\, R_{\text{old}}(:,1) - (v \cdot R_{\text{old}}(:,1))\, R_{\text{old}}(:,2) = [1 \;\; 1 \;\; 0 \;\; 0]^T \\ r_{23} &= (v \cdot R_{\text{old}}(:,2))\, R_{\text{old}}(:,3) - (v \cdot R_{\text{old}}(:,3))\, R_{\text{old}}(:,2) = [0 \;\; 1 \;\; 1 \;\; 0]^T. \end{aligned}$$

Determining the zero set of r_{21}, we have $Z(r_{21}) = \{3,4,5,6\}$. This induces the following zero-set matrix for r_{21}:

$$A_{Z(r_{21})} = \begin{bmatrix} 0 & 0 & 1 & 0 \\ 0 & 0 & 0 & 1 \\ -1 & 1 & -1 & 0 \\ 1 & -1 & 1 & 0 \end{bmatrix}$$

Computing the rank of $A_{Z(r_{21})}$ gives us 3 which makes r_{21} an extreme ray. On the other hand, the zero set of r_{23} is given by $Z(r_{23}) = \{1,4,5,6\}$ which induces the following zero-set matrix:

$$A_{Z(r_{23})} = \begin{bmatrix} 1 & 0 & 0 & 0 \\ 0 & 0 & 0 & 1 \\ -1 & 1 & -1 & 0 \\ 1 & -1 & 1 & 0 \end{bmatrix}$$

Computing the rank of $A_{Z(r_{23})}$ gives us 3 which also makes r_{23} an extreme ray. Adding r_{21} and r_{23} to R_{old} and discarding $R_{\text{old}}(:,1)$, $R_{\text{old}}(:,2)$, and $R_{\text{old}}(:,3)$ gives us

$$R_{\text{new}} = \begin{bmatrix} 0 & 1 & 0 \\ 0 & 1 & 1 \\ 0 & 0 & 1 \\ 1 & 0 & 0 \end{bmatrix}.$$

Lastly, we update $k = 6 + 2 = 8$. We also let $R_{\text{old}} := R_{\text{new}}$ and $A_{\text{old}} := A_{\text{new}}$.

Iteration 2. Choose $v := N_{2:} = [1 \;\; -1 \;\; 1 \;\; 0]$. So, A_{new} becomes

$$\left[\begin{array}{cccc|cc|cc} 1 & 0 & 0 & 0 & -1 & 1 & 1 & -1 \\ 0 & 1 & 0 & 0 & 1 & -1 & -1 & 1 \\ 0 & 0 & 1 & 0 & -1 & 1 & 1 & -1 \\ 0 & 0 & 0 & 1 & 0 & 0 & 0 & 0 \end{array}\right]^T.$$

We only have 3 columns for R_{old}. So, $\Lambda = \{1,2,3\}$. Multiplying v with the columns of R_{old} results in the following partition:

$$\Lambda_+ = \{\} \qquad \Lambda_0 = \{1,2,3\} \qquad \Lambda_- = \{\}$$

There are no elements in Λ_+ and Λ_-, hence no new vector shall be computed as candidate extreme ray for this particular step. So, we update $k = 10$ and let $A_{\text{old}} := A_{\text{new}}$.

Iteration 3. Choose $v := N_{3:} = [0 \;\; -1 \;\; 1 \;\; 1]$. So,

$$A_{\text{new}} = \left[\begin{array}{cccc|ccc|ccc} 1 & 0 & 0 & 0 & -1 & 1 & 0 & 1 & -1 & 0 \\ 0 & 1 & 0 & 0 & 1 & -1 & -1 & -1 & 1 & 1 \\ 0 & 0 & 1 & 0 & -1 & 1 & 1 & 1 & -1 & -1 \\ 0 & 0 & 0 & 1 & 0 & 0 & 1 & 0 & 0 & -1 \end{array}\right]^T.$$

We still have 3 columns of R_{old}. So, $\Lambda = \{1, 2, 3\}$. Multiplying v with the columns of R_{old} results in the following partition:

$$\Lambda_+ = \{1\} \qquad \Lambda_0 = \{3\} \qquad \Lambda_- = \{2\}$$

There is one possible combination from $\Lambda_+ \times \Lambda_-$, namely r_{12}. Determining the entries of this ray,

$$r_{12} = (v \cdot R_{\text{old}}(:,1))\, R_{\text{old}}(:,2) - (v \cdot R_{\text{old}}(:,2))\, R_{\text{old}}(:,1) = [1 \;\; 1 \;\; 0 \;\; 1]^T.$$

Determining its zero set, we have $Z(r_{12}) = \{3, 5, 6, 7, 8, 9, 10\}$ which induces the following zero-set matrix

$$A_{Z(r_{12})} = \begin{bmatrix} 0 & -1 & 1 & 0 & 1 & -1 & 0 \\ 0 & 1 & -1 & -1 & -1 & 1 & 1 \\ 1 & -1 & 1 & 1 & 1 & -1 & -1 \\ 0 & 0 & 0 & 1 & 0 & 0 & -1 \end{bmatrix}^T.$$

Computing the rank of $A_{Z(r_{12})}$ gives us 3 which makes r_{12} an extreme ray. So, we add r_{12} to R_{old} and discard $R_{\text{old}}(:,1)$ and $R_{\text{old}}(:,2)$. This gives us

$$R_{\text{new}} = \begin{bmatrix} 0 & 1 \\ 1 & 1 \\ 1 & 0 \\ 0 & 1 \end{bmatrix}.$$

We then update $k = 12$ and let $R_{\text{old}} := R_{\text{new}}$ and $A_{\text{old}} := R_{\text{new}}$.

Iteration 4. We now choose the remaining row of N left unchosen which is $v := N_{4:} = [0 \;\; 1 \;\; -1 \;\; -1]$. So,

$$A_{\text{new}} = \left[\begin{array}{cccc|cccc|cccc} 1 & 0 & 0 & 0 & -1 & 1 & 0 & 0 & 1 & -1 & 0 & 0 \\ 0 & 1 & 0 & 0 & 1 & -1 & -1 & 1 & -1 & 1 & 1 & -1 \\ 0 & 0 & 1 & 0 & -1 & 1 & 1 & -1 & 1 & -1 & -1 & 1 \\ 0 & 0 & 0 & 1 & 0 & 0 & 1 & -1 & 0 & 0 & -1 & 1 \end{array}\right]^T.$$

We note that $A_{\text{new}} = A$. We only have 2 columns in R_{old}. So, $\Lambda = \{1, 2\}$. Multiplying v with the columns of R_{old} results in the following partition:

$$\Lambda_+ = \{\} \qquad \Lambda_0 = \{1, 2\} \qquad \Lambda_- = \{\}$$

Since no new vector shall be computed as candidate extreme ray, we update $k = 14$ and let $A_{\text{old}} := A_{\text{new}}$. Now, since $k = 14 = 4 + 2 \cdot 4 + 2 = r + 2m + 2$, where m is the number of species in the network, we exit with the algorithm leaving us the final matrix R as follows:

$$\begin{bmatrix} 0 & 1 \\ 1 & 1 \\ 1 & 0 \\ 0 & 1 \end{bmatrix}$$

The columns of R correspond to the EFMs of the network which are $e_1 = \{r_2, r_3\}$ and $e_2 = \{r_1, r_2, r_4\}$.

Construction of the reaction-to-reaction graph

We recall that based on Theorem 3 of the main text, we need to construct a reaction-to-reaction graph $G^{\mathcal{R}} = (V^{\mathcal{R}}, E^{\mathcal{R}})$ that is both CS- and EM-compatible to a given CRN $(\mathcal{S}, \mathcal{C}, \mathcal{R})$. We formulate this as a BLP problem by introducing binary decision variables

$$x_{ij} \in \{0, 1\} \tag{C1}$$

for $i, j = 1, 2, \ldots, r$, $i \neq j$, such that $x_{ij} = 1$ if and only if $(r_i, r_j) \in E^{\mathcal{R}}$ [4]. To guarantee that we will get a CS- and EM-compatible reaction-to-reaction graph to a given CRN, we set up the appropriate constraints using the set of EFMs $\mathcal{E}$ and the set of sets of reactions with shared source complexes $\mathcal{F}$. Formally, the set $\mathcal{F}$ is given by $\mathcal{F} = \{f_1, f_2, \ldots, f_q\}$, where $r_i, r_j \in f_k$, $i \neq j$, for some k if $y_{s(i)} = y_{s(j)}$.

Based on the sets $\mathcal{E}$ and $\mathcal{F}$, we observe that we can identify clusters of reactions that do not interact with other clusters. These clusters would eventually form the disconnected components in the reaction-to-reaction graph. To determine these clusters, we introduce the relation '≡' with the following properties:

- $e_i \equiv e_j$ if $e_i \cap e_j \neq \emptyset$,
- $e_i \equiv e_j$ if there are $r_k \in e_i$ and $r_l \in e_j$ such that $r_k, r_l \in f_u$ for some $f_u \in \mathcal{F}$.

The first property tells us that two EFMs are "related" if they share a reaction. Meanwhile, the second property states that two EFMs are "related" if there is/are reaction/s in each EFM with the same source complex. Given this, we impose the following constraint:

$$x_{ij} = 0 \quad \text{if } r_i \in e_k \text{ and } r_j \in e_l \text{ where } e_k \not\equiv e_l. \tag{C2}$$

To ensure CS-compatibility, we impose that, if $r_i, r_j \in f_l$ for some $f_l \in \mathcal{F}$, then

$$x_{ki} - x_{kj} = 0, \qquad k = 1, 2, \ldots, r. \tag{C3}$$

For EM-compatibility, we impose the following constraints:

$$\begin{aligned} \sum_{r_i, r_j \in e_k} x_{ij} &= |e_k|, \\ \sum_{r_i \in e_k} x_{ij} &= 1, \qquad r_j \in e_k, \\ \sum_{r_j \in e_k} x_{ij} &= 1, \qquad r_i \in e_k. \end{aligned} \tag{C4}$$

The first constraint set above guarantees that the number of edges in the reaction-to-reaction graph corresponding to an EFM is precisely the number of reactions in that EFM. The second and third constraint sets ensure that each vertex in the reaction-to-reaction graph has exactly one outgoing edge and an incoming edge with respect to an EFM, respectively. As a whole, the constraint sets in (C4) guarantees that every reaction belonging to an EFM is part of exactly one cycle on the support of that EFM [4].

Since EM-compatibility requires minimal directed cycles in the reaction-to-reaction graph, we further impose that EFMs can no longer be decomposed into subcycles by introducing a subcycle/subtour elimination constraint. In particular, we impose that, for every EFM $e_k \in \mathcal{E}$ with $l(k) := |e_k| \geq 4$, every combination $e'_k = \{r_{\mu(1)}, \ldots, r_{\mu(l(k'))}\} \subset e_k$ with $2 \leq |e'_k| \leq \left\lfloor \dfrac{|e_k|}{2} \right\rfloor$ satisfies the following:

$$\sum_{r_i, r_j \in e'_k} x_{ij} \leq |e'_k| - 1. \tag{C5}$$

For the objective function, we don't want to add unnecessary edges $(r_i, r_j) \in E^{\mathcal{R}}$. So, we impose the following:

$$\min \sum_{\substack{i,j=1 \\ i \neq j}}^{r} x_{ij}, \tag{C6}$$

which is equivalent to minimizing the number of edges in $(r_i, r_j) \in E^{\mathcal{R}}$.

Therefore, the complete BLP model that determines - if there exists a feasible solution - a reaction-to-reaction graph that is CS- and EM-compatible to a given CRN is by optimizing (C6) over the constraint sets (C1), (C2), (C3), (C4), and (C5) [4].

Now, going back to our histidine kinase system, we have computed that there are two EFMs, which are $e_1 = \{r_2, r_3\}$ and $e_2 = \{r_1, r_2, r_4\}$. Moreover, it is clear from the network that all four reactions have different source complexes. So, we have $\mathcal{F} = \emptyset$. Note that r_2 appears in both EFMs which means that $e_1 \equiv e_2$. Hence, there are no constraints of the form (C2). Since the set $\mathcal{F}$ is empty, it implies that there are also no constraints of the form (C3). Lastly, the

network only has two EFMs which is fewer than the minimum required to warrant inclusion of subcycle elimination constraints. Hence, there are no constraints of the form (C5).

This only leaves us with constraint sets of the form (C1) and (C4). Therefore, the BLP model to determine a reaction-to-reaction graph that is CS- and EM- compatible with the histidine kinase network is given by:

$$\begin{aligned}\min \quad & x_{12}+x_{13}+x_{14}+x_{21}+x_{23}+x_{24}+x_{31}+x_{32}+x_{34}+x_{41}+x_{42}+x_{43}\\ & \text{subject to}\\ & \begin{cases} x_{23}+x_{32}=2\\ x_{12}+x_{14}+x_{21}+x_{24}+x_{41}+x_{42}=3\\ x_{32}=1\\ x_{23}=1\\ x_{21}+x_{41}=1\\ x_{12}+x_{42}=1\\ x_{14}+x_{24}=1\\ x_{12}+x_{14}=1\\ x_{21}+x_{24}=1\\ x_{41}+x_{42}=1\\ x_{ij}\in\{0,1\}\ \ i,j=1,2,3,4,\quad i\neq j \end{cases} .\end{aligned} \tag{C7}$$

Using `intlinprog()` via MATLAB, the solution to the binary linear program (C7) is given by

$$\begin{bmatrix}0&0&0&1\\1&0&1&0\\0&1&0&0\\0&1&0&0\end{bmatrix},$$

where the ij-th entry of the matrix corresponds to the edge from reaction i to reaction j. If the entry is 1, then there exists a directed edge from reaction i to reaction j in the reaction-to-reaction graph; if the entry is 0, no such edge is present. This is the matrix form of the reaction-to-reaction graph shown below.

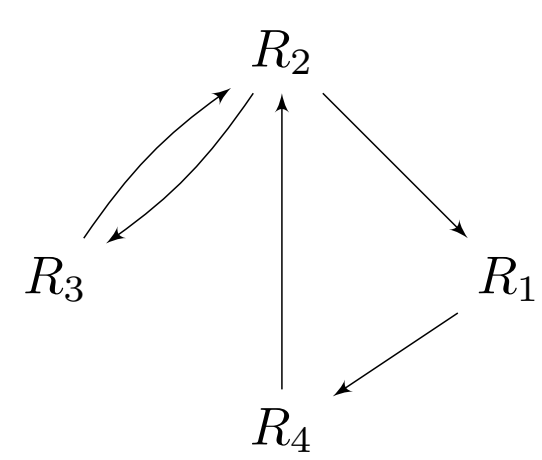

Determination of the translation complexes

The optimization procedure in the previous subsection may create cycles in the reaction-to-reaction graph that do not correspond to minimal EFMs [4]. This implies that the resulting reaction-to-reaction graph fails to be EM-compatible with the reaction network. In this case, Eq (6) of Theorem 3 in the main text will be inconsistent [4]. Therefore, prior to solving the system, we first verify its consistency. If the system is inconsistent, then the procedure fails to construct a WR and DZ translation. Otherwise, we proceed to solve the translation complexes α_i, $i=1,\dots,r$. This serves as a preprocessing step before proceeding to the final stage of the overall network translation procedure.

We now show the step-by-step procedure in the computation of the translation complexes, as outlined in Algorithm 2 in the main text, necessary to construct a WR and DZ translation of the histidine kinase network. Checking for consistency, we construct the matrix equation $A\alpha=b$ of the histidine kinase network and wish to determine whether $\text{rank}(A)=\text{rank}(B)$ where B is the augmented matrix $B=[A \mid b]$. If the ranks of the associated matrices are equal, then Eq (6) of Theorem 3 is consistent and we can proceed to determining the translation complexes $\alpha_i, i=1,2,3,4$. So,

$$\begin{bmatrix} 1 & 0 & 0 & -1 \\ -1 & 1 & 0 & 0 \\ 0 & 1 & -1 & 0 \\ 0 & -1 & 1 & 0 \\ 0 & -1 & 0 & 1 \end{bmatrix} \begin{bmatrix} \alpha_1 \\ \alpha_2 \\ \alpha_3 \\ \alpha_4 \end{bmatrix} = \begin{bmatrix} 0 & -1 & 0 & 1 \\ 0 & 0 & 0 & -1 \\ 0 & 0 & 0 & 0 \\ 0 & 0 & 0 & 0 \\ 0 & 1 & 0 & 0 \end{bmatrix},$$

where $\alpha_1, \alpha_2, \alpha_3$, and α_4 are the translation complexes for R_1, R_2, R_3, and R_4, respectively. We now compute the ranks of matrices A and B which are given by the following:

$$\text{rank}(A) = \text{rank}\left(\begin{bmatrix} 1 & 0 & 0 & -1 \\ -1 & 1 & 0 & 0 \\ 0 & 1 & -1 & 0 \\ 0 & -1 & 1 & 0 \\ 0 & -1 & 0 & 1 \end{bmatrix}\right) = 3,$$

$$\text{rank}(B) = \text{rank}([A \mid b]) = \text{rank}\left(\left[\begin{array}{cccc|cccc} 1 & 0 & 0 & -1 & 0 & -1 & 0 & 1 \\ -1 & 1 & 0 & 0 & 0 & 0 & 0 & -1 \\ 0 & 1 & -1 & 0 & 0 & 0 & 0 & 0 \\ 0 & -1 & 1 & 0 & 0 & 0 & 0 & 0 \\ 0 & -1 & 0 & 1 & 0 & 1 & 0 & 0 \end{array}\right]\right) = 3.$$

Since $\text{rank}(A) = \text{rank}(B)$, the system is consistent. We can now proceed to Step 2 of Algorithm 2. For easier reference, we note that the edges of the reaction-to-reaction graph computed in the previous step are as follows:

$$E^{\mathcal{R}} = \{(r_1, r_4), (r_2, r_1), (r_2, r_3), (r_3, r_2), (r_4, r_2)\}.$$

We first initialize the following sets:

$$\mathcal{P} = \{r_1, r_2, r_3, r_4\} \quad \mathcal{P}' = \{\} \quad \mathcal{P}'' = \{\}$$

Selecting r_2 as the first reaction to process, we set $\alpha_2 = 0$. Updating $\mathcal{P}'$ and $\mathcal{P}$,

$$\mathcal{P}' = \{r_2\} \quad \mathcal{P} = \{r_1, r_3, r_4\}$$

We then choose a $r_j \in \mathcal{P}$ such that $(r_2, r_j) \in E^{\mathcal{R}}$. There are two possible reactions: r_1 or r_3. Suppose we choose r_1. So,

$$\alpha_1 = X + Y_p - X + 0 = Y_p$$

We then mark r_2 as processed and update the following sets:

$$\mathcal{P}' = \{r_1\} \quad \mathcal{P} = \{r_3, r_4\} \quad \mathcal{P}'' = \{\}$$

Now, we choose another reaction $r_j \in \mathcal{P}$ such that $(r_1, r_j) \in E^{\mathcal{R}}$. There is only one possible reaction, which is r_4. So,

$$\alpha_4 = X_p - Y_p + Y_p = X_p$$

Marking r_4 as processed as updating the sets $\mathcal{P}, \mathcal{P}', \mathcal{P}''$, we get:

$$\mathcal{P}' = \{r_4\} \quad \mathcal{P} = \{r_3\} \quad \mathcal{P}'' = \{\}$$

There are no longer reactions $r_j \in \mathcal{P}$ such that $(r_4, r_j) \in E^{\mathcal{R}}$. Note that $(r_4, r_3) \notin E^{\mathcal{R}}$. So, we choose the remaining unprocessed reaction r_3 and set $\alpha_3 = 0$. After processing r_3, we get the following sets:

$$\mathcal{P}' = \{r_3\} \quad \mathcal{P} = \{\} \quad \mathcal{P}'' = \{\}$$

Since there are no longer reactions in $\mathcal{P}$ (or equivalently, all reactions have already been processed), we stop the algorithm and return the following translation complexes:

$$\alpha_1 = Y_p \quad \alpha_2 = 0 \quad \alpha_3 = 0 \quad \alpha_4 = X_p$$

Adding the computed translation complexes to their corresponding reactions, we get the following WR and DZ translation of the histidine kinase network:

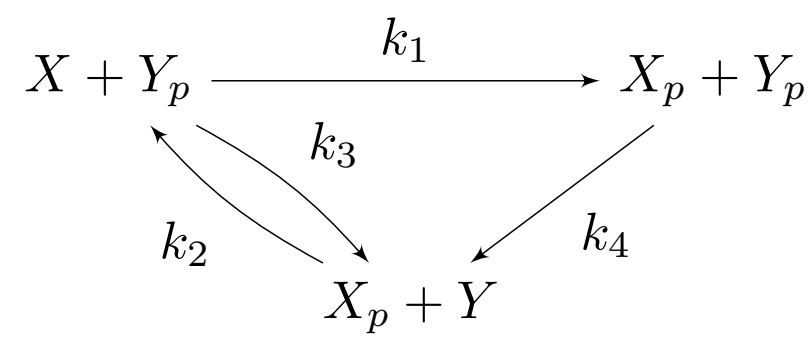

As we can observe, the translated complexes are all nonnegative. So, we no longer need to perform Step 7 of the algorithm.

Proof of multistationarity in the EnvZ-OmpR system

As discussed in the main text, we have the following cubic polynomial in the concentration of X_pY:

$$\left(\gamma_1\alpha_6\gamma_2 - \gamma_1^2\alpha_6\right)(X_pY)^3 + \left(2T_1\gamma_1\alpha_6 - T_1\alpha_6\gamma_2 + \alpha_6^2 - \gamma_1\alpha_6\alpha_7 + \gamma_1\alpha_6T_2\right)(X_pY)^2 + (T_1\alpha_6\alpha_7 - T_1^2\alpha_6 - T_1\alpha_6T_2)X_pY = 0$$

where $\alpha_1, \alpha_2, \alpha_3, \alpha_4, \alpha_5, \alpha_6, \alpha_7, T_1, T_2 > 0$, and $\gamma_1 = 1 + \alpha_1 + \alpha_2 + \alpha_3 + \alpha_4 + \alpha_5$ and $\gamma_2 = \alpha_1 + \alpha_2 + \alpha_3$. We argue that the cubic polynomial has two sign changes.

We first consider $\gamma_1\alpha_6\gamma_2 - \gamma_1^2\alpha_6$.

$$\begin{aligned}\gamma_1\alpha_6\gamma_2 - \gamma_1^2\alpha_6 &= \gamma_1\alpha_6(\gamma_2 - \gamma_1)\\ &= \gamma_1\alpha_6(\alpha_1 + \alpha_2 + \alpha_3 - (1 + \alpha_1 + \alpha_2 + \alpha_3 + \alpha_4 + \alpha_5))\\ &= -\gamma_1\alpha_6(1 + \alpha_4 + \alpha_5) < 0\end{aligned}$$

We then consider the coefficient for X_pY which is given by $T_1\alpha_6\alpha_7 - T_1^2\alpha_6 - T_1\alpha_6T_2$.

$$\begin{aligned}T_1\alpha_6\alpha_7 - T_1^2\alpha_6 - T_1\alpha_6T_2 &= T_1\alpha_6(\alpha_7 - T_1 - T_2)\\ &= T_1\alpha_6(\alpha_7 - (T_1 + T_2))\end{aligned}$$

Note that we have the following conservation equations:

$$\begin{aligned}X + XD + XDY_p + XT + XTY_p + X_p + X_pY &= T_1\\ -X - XD - XT - X_p + Y + Y_p &= T_2\end{aligned}$$

Adding the equations, we get $T_1 + T_2 = XDY_p + XTY_p + X_pY + Y + Y_p$. Moreover, we note that $\alpha_7 = Y_p$. So, $T_1 + T_2 > \alpha_7$ which implies that $T_1\alpha_6\alpha_7 - T_1^2\alpha_6 - T_1\alpha_6T_2 < 0$.

Lastly, we consider the coefficienct of the middle term of the polynomial which is given by $2T_1\gamma_1\alpha_6 - T_1\alpha_6\gamma_2 + \alpha_6^2 - \gamma_1\alpha_6\alpha_7 + \gamma_1\alpha_6T_2$.

$$\begin{aligned}2T_1\gamma_1\alpha_6 - T_1\alpha_6\gamma_2 + \alpha_6^2 - \gamma_1\alpha_6\alpha_7 + \gamma_1\alpha_6T_2 &= \alpha_6(2T_1\gamma_1 - T_1\gamma_2 + \alpha_6 - \gamma_1\alpha_7 + \gamma_1T_2)\\ &= \alpha_6(\alpha_6 + \gamma_1(2T_1 - \alpha_7 + T_2) - T_1\gamma_2)\\ &= \alpha_6(\alpha_6 + T_1\gamma_1 + \gamma_1(T_1 + T_2 - \alpha_7) - T_1\gamma_2\\ &= \alpha_6 + T_1(1 + \alpha_4 + \alpha_5) + \gamma_1(T_1 + T_2 - \alpha_7)\end{aligned}$$

Since $T_1 + T_2 > \alpha_7$, then $2T_1\gamma_1\alpha_6 - T_1\alpha_6\gamma_2 + \alpha_6^2 - \gamma_1\alpha_6\alpha_7 + \gamma_1\alpha_6T_2 > 0$ which proves our claim that the cubic polynomial has two sign changes.

Detailed computation of conservation laws in the EnvZ-OmpR model

Conservation laws of a CRN can be computed by determining the left null space of its stoichiometric matrix [5–7]. That is, a conservation equation is represented by a vector c such that

$$cN = \mathbf{0}, \tag{C8}$$

where N is the stoichiometric matrix. Equivalently, finding the vector c requires solving the null space of N^T. In our EnvZ-OmpR example, the stoichiometric matrix N is given by

$$N = \begin{array}{c} \\ X \\ XD \\ XDY_p \\ XT \\ XTY_p \\ X_p \\ X_pY \\ Y \\ Y_p \end{array} \begin{array}{c} \begin{array}{cccccccccccccc} R_1 & R_2 & R_3 & R_4 & R_5 & R_6 & R_7 & R_8 & R_9 & R_{10} & R_{11} & R_{12} & R_{13} & R_{14} \end{array} \\ \begin{bmatrix} 1 & -1 & -1 & 1 & 0 & 0 & 0 & 1 & 0 & 0 & 0 & 0 & 0 & 0 \\ -1 & 1 & 0 & 0 & 0 & 0 & 0 & 0 & -1 & 1 & 1 & 0 & 0 & 0 \\ 0 & 0 & 0 & 0 & 0 & 0 & 0 & 0 & 1 & -1 & -1 & 0 & 0 & 0 \\ 0 & 0 & 1 & -1 & -1 & 0 & 0 & 0 & 0 & 0 & 0 & -1 & 1 & 1 \\ 0 & 0 & 0 & 0 & 0 & 0 & 0 & 0 & 0 & 0 & 0 & 1 & -1 & -1 \\ 0 & 0 & 0 & 0 & 1 & -1 & 1 & 0 & 0 & 0 & 0 & 0 & 0 & 0 \\ 0 & 0 & 0 & 0 & 0 & 1 & -1 & -1 & 0 & 0 & 0 & 0 & 0 & 0 \\ 0 & 0 & 0 & 0 & 0 & -1 & 1 & 0 & 0 & 0 & 1 & 0 & 0 & 1 \\ 0 & 0 & 0 & 0 & 0 & 0 & 0 & 1 & -1 & 1 & 0 & -1 & 1 & 0 \end{bmatrix} \end{array}$$

Performing Gaussian elimination on N^T to obtain its RREF gives us

$$\begin{array}{c} \begin{array}{ccccccccc} X & XD & XDY_p & XT & XTY_p & X_p & X_pY & Y & Y_p \end{array} \\ \begin{bmatrix} 1 & 0 & 0 & 0 & 0 & 0 & -1 & 0 & 1 \\ 0 & 1 & 0 & 0 & 0 & 0 & -1 & 0 & 1 \\ 0 & 0 & 1 & 0 & 0 & 0 & -1 & 0 & 0 \\ 0 & 0 & 0 & 1 & 0 & 0 & -1 & 0 & 1 \\ 0 & 0 & 0 & 0 & 1 & 0 & -1 & 0 & 0 \\ 0 & 0 & 0 & 0 & 0 & 1 & -1 & 0 & 1 \\ 0 & 0 & 0 & 0 & 0 & 0 & 0 & 1 & -1 \\ 0 & 0 & 0 & 0 & 0 & 0 & 0 & 0 & 0 \\ 0 & 0 & 0 & 0 & 0 & 0 & 0 & 0 & 0 \\ 0 & 0 & 0 & 0 & 0 & 0 & 0 & 0 & 0 \\ 0 & 0 & 0 & 0 & 0 & 0 & 0 & 0 & 0 \\ 0 & 0 & 0 & 0 & 0 & 0 & 0 & 0 & 0 \\ 0 & 0 & 0 & 0 & 0 & 0 & 0 & 0 & 0 \\ 0 & 0 & 0 & 0 & 0 & 0 & 0 & 0 & 0 \end{bmatrix} \end{array}$$

which corresponds to the vector c as in Eq C8

$$c = \begin{bmatrix} X_pY - Y_p \\ X_pY - Y_p \\ X_pY \\ X_pY - Y_p \\ X_pY \\ X_pY - Y_p \\ X_pY \\ Y_p \\ Y_p \end{bmatrix} = X_pY \begin{bmatrix} 1 \\ 1 \\ 1 \\ 1 \\ 1 \\ 1 \\ 1 \\ 0 \\ 0 \end{bmatrix} + Y_p \begin{bmatrix} -1 \\ -1 \\ 0 \\ -1 \\ 0 \\ -1 \\ 0 \\ 1 \\ 1 \end{bmatrix},$$

where X_pY and Y_p are the free variables in the RREF of N^T. This yields two basis vectors in the left null space of N. We can use any two different basis vectors by setting particular values for the free variables to get a set of valid conservation laws of the system as they span the exact same left null space of N. In particular, setting $X_pY = 1$ and $Y_p = 0$ gives us the conservation equation

$$X + XD + XDY_p + XT + XTY_p + X_p + X_pY = T_1, \tag{C9}$$

where T_1 is a constant determined by the initial concentrations of the species involved in the equation. Furthermore, if we set $X_pY = 0$ and $Y_p = 1$, we get

$$-X - XD - XT - X_p + Y + Y_p = T_2, \tag{C10}$$

where T_2 is likewise a constant determined by the initial concentrations of the species involved in the equation. These are the conservation equations obtained by CRITERIA. Notice that we can also set $X_pY = 1$ and $Y_p = 1$ to get the equation

$$XDY_p + XTY_p + X_pY + Y + Y_p = T_3, \tag{C11}$$

which is also a valid conservation law together with either of the conservation equations above. This is the same as adding the corresponding left and right sides of Eq C9 and Eq C10 to obtain Eq C11 with $T_3 = T_1 + T_2$, which is also a constant.

CRITERIA: An enhanced computational package for computing equilibria

We automate the method for computing the equilibria of biochemical reaction networks discussed in the main text by developing a user-friendly, open-source, and publicly available computational package written in MATLAB. For easier reference, we call the package CRITERIA (Computing paRametrized posITive EquilibRIA). The package decomposes a given reaction network into its finest independent decomposition, performs the EFM–based translation for each subnetwork, and then computes the parameterization of the positive equilibria of the resulting network. It also outputs the kinetic deficiency of the generalized CRN as well as the additional conditions required to obtain the final parametrization when the kinetic deficiency is positive (refer to Case 2 of Theorem 2 in the main text). Finally, CRITERIA provides further information by enumerating the free parameters and conservation laws of the system.

Manual for the code

We build upon COMPILES in the development of CRITERIA, in the sense that the inputs of both programs are similar, with some modifications. In this subsection, we describe how to provide the input of CRITERIA and run the main code, `ComputeEquilibria.m`, using the EnvZ–OmpR system as our illustration.

Step 1) Make sure that the files `addReaction.m`, `edge.m`, `graph_.m`, and `vertex.m` are in the same working directory as `steadyState.m`.

Step 2) As with COMPILES, create a structure named `model` by inputting a name for your model in `model.id`.

```
model.id = ‘EnvZ-OmpR System’;
```

Step 3) Use the function `addReaction` to add the reactions of the CRN to the structure `model`. The string in the first line serves only as a visual guide indicating the form of the reaction. The second line specifies the reactant species (strings), their corresponding stoichiometries (cell list), and their respective kinetic orders (array list). The third line specifies the product species with the same inputs as for the reactant species. Finally, the fourth line is a Boolean value indicating whether the reaction is reversible or irreversible.

In contrast to COMPILES, we introduce the following modification in CRITERIA: reversible reactions must be added separately. In particular, the input for the kinetic orders of the product species is left empty, and the fourth line is set to `false`, indicating that reactions are added one at a time, regardless of whether they are part of a reversible pair.

```
model = addReaction(model,‘XD->X’, ...
                          {‘XD’}, {1}, [1], ...
                          {‘X’}, {1}, [ ], ...
                          false);
model = addReaction(model,‘X->XD’, ...
                          {‘X’}, {1}, [1], ...
                          {‘XD’}, {1}, [ ], ...
                          false);
model = addReaction(model,‘X->XT’, ...
                          {‘X’}, {1}, [1], ...
                          {‘XT’}, {1}, [ ], ...
                          false);
model = addReaction(model,‘XT->X’, ...
                          {‘XT’}, {1}, [1], ...
                          {‘X’}, {1}, [ ], ...
                          false);
model = addReaction(model,‘XT->Xp’, ...
```

```
                                {‘XT’}, {1}, [1], ...
                                {‘Xp’}, {1}, [ ], ...
                                false);
model = addReaction(model,‘Xp+Y->XpY’, ...
                                {‘Xp’, ‘Y’}, {1, 1}, [1, 1], ...
                                {‘XpY’}, {1}, [ ], ...
                                false);
model = addReaction(model,‘XpY->Xp+Y’, ...
                                {‘XpY’}, {1}, [1], ...
                                {‘Xp’, ‘Y’}, {1, 1}, [ ], ...
                                false);
model = addReaction(model,‘XpY->X+Yp’, ...
                                {‘XpY’}, {1}, [1], ...
                                {‘X’, ‘Yp’}, {1, 1}, [ ], ...
                                false);
model = addReaction(model,‘XD+Yp->XDYp’, ...
                                {‘XD’, ‘Yp’}, {1, 1}, [1, 1], ...
                                {‘XDYp’}, {1}, [ ], ...
                                false);
model = addReaction(model,‘XDYp->XD+Yp’, ...
                                {‘XDYp’}, {1}, [1], ...
                                {‘XD’, ‘Yp’}, {1, 1}, [ ], ...
                                false);
model = addReaction(model,‘XDYp->XD+Y’, ...
                                {‘XDYp’}, {1}, [1], ...
                                {‘XD’, ‘Y’}, {1, 1}, [ ], ...
                                false);
model = addReaction(model,‘XT+Yp->XTYp’, ...
                                {‘XT’, ‘Yp’}, {1, 1}, [1, 1], ...
                                {‘XTYp’}, {1}, [ ], ...
                                false);
model = addReaction(model,‘XTYp->XT+Yp’, ...
                                {‘XTYp’}, {1}, [1], ...
                                {‘XT’, ‘Yp’}, {1, 1}, [ ], ...
                                false);
model = addReaction(model,‘XTYp->XT+Y’, ...
                                {‘XTYp’}, {1}, [1], ...
                                {‘XT’, ‘Y’}, {1, 1}, [ ], ...
                                false);
```

Step 4) Finally, simply run the function `ComputeEquilibria` with the input `model` and outputs `equation`, `species`, `free_parameter`, `conservation_law`, and `model`, which are similar to COMPILES.

```
[equation, species, free_parameter, conservation_law, model] = steadyState(model);
```

Output) When executed, the function `ComputeEquilibria` first displays the given CRN based on the reactions that were added. It then outputs the number of independent subnetworks in the CRN, followed by the reactions belonging to each subnetwork. For each subnetwork, the program indicates whether a weakly reversible and deficiency

zero translation was successfully found. If successful, the translated reactions for the corresponding subnetwork are displayed. Otherwise, a message is issued indicating that no valid translation was found for that subnetwork.

If the procedure is successful for all subnetworks, the function then outputs the kinetic deficiency of the resulting generalized CRN along with the additional conditions required for the final parametrization when the kinetic deficiency is positive. Furthermore, the function outputs the parametrized equilibria of the system. For reference, the lists of free parameters and conservation laws are also displayed. Based on the kinetic deficiency, the function outputs how many free parameters can be solved using the additional conditions. Finally, the elapsed computation time of the program is reported.

```
- Network -

R1:  XD->X
R2:  X->XD
R3:  X->XT
R4:  XT->X
R5:  XT->Xp
R6:  Xp+Y->XpY
R7:  XpY->Xp+Y
R8:  XpY->X+Yp
R9:  XD+Yp->XDYp
R10:  XDYp->XD+Yp
R11:  XDYp->XD+Y
R12:  XT+Yp->XTYp
R13:  XTYp->XT+Yp
R14:  XTYp->XT+Y

Solving the network...

The network has 2 subnetworks.

- Subnetwork 1 -

R1:  XD->X
R2:  X->XD

Solving Subnetwork 1...

We found a weakly reversible and deficiency zero translation.
R1:  XD->X
R2:  X->XD

- Subnetwork 2 -

R3:  X->XT
R4:  XT->X
R5:  XT->Xp
R6:  Xp+Y->XpY
R7:  XpY->Xp+Y
R8:  XpY->X+Yp
R9:  XD+Yp->XDYp
R10:  XDYp->XD+Yp
R11:  XDYp->XD+Y
R12:  XT+Yp->XTYp
R13:  XTYp->XT+Yp
R14:  XTYp->XT+Y

Solving Subnetwork 2...
```

```
We found a weakly reversible and deficiency zero translation.
R3:  X + XD + XT + Yp -> XD + 2XT + Yp
R4:  XD + 2XT + Yp -> X + XD + XT + Yp
R5:  XD + 2XT + Yp -> XD + XT + Xp + Yp
R6:  XD + XT + Xp + Y -> XD + XT + XpY
R7:  XD + XT + XpY -> XD + XT + Xp + Y
R8:  XD + XT + XpY -> X + XD + XT + Yp
R9:  XD + XT + Xp + Yp -> XDYp + XT + Xp
R10:  XDYp + XT + Xp -> XD + XT + Xp + Yp
R11:  XDYp + XT + Xp -> XD + XT + Xp + Y
R12:  XD + XT + Xp + Yp -> XD + XTYp + Xp
R13:  XD + XTYp + Xp -> XD + XT + Xp + Yp
R14:  XD + XTYp + Xp -> XD + XT + Xp + Y

-----------------------
Kinetic deficiency:  1
-----------------------

---------------------------------------------
Additional conditions to the parametrization:
Eq 1:  1 = (k1*k3*k12)/(k2*sigma1*(k4 + k5))
---------------------------------------------

X = (k8*tau1*(k4 + k5))/(k3*k5)
XD = (k2*k8*tau1*(k4 + k5))/(k1*k3*k5)
XDYp = (k8*k9*tau1*(k13 + k14))/(k9*k11*k13 + k9*k11*k14 + k10*k14*sigma1 + k11*k14*sigma1)
XT = (k8*tau1)/k5
XTYp = (k8*sigma1*tau1*(k10 + k11))/(k9*k11*k13 + k9*k11*k14 + k10*k14*sigma1 + k11*k14*sigma1)
Xp = (tau1*(k7 + k8))/(k6*tau2)
XpY = tau1
Y = tau2
Yp = (k5*sigma1*(k10 + k11)*(k13 + k14))/(k12*(k9*k11*k13 + k9*k11*k14 + k10*k14*sigma1 +
k11*k14*sigma1))

Solving positive steady state parametrization of the network...

The solution is:

X = (XpY*k8*(k4 + k5))/(k3*k5)
XD = (XpY*k2*k8*(k4 + k5))/(k1*k3*k5)
XDYp = (XpY*k8*k9*(k13 + k14))/(k9*k11*k13 + k9*k11*k14 + k10*k14*sigma1 + k11*k14*sigma1)
XT = (XpY*k8)/k5
XTYp = (XpY*k8*sigma1*(k10 + k11))/(k9*k11*k13 + k9*k11*k14 + k10*k14*sigma1 + k11*k14*sigma1)
Xp = (XpY*(k7 + k8))/(Y*k6)
Yp = (k5*sigma1*(k10 + k11)*(k13 + k14))/(k12*(k9*k11*k13 + k9*k11*k14 + k10*k14*sigma1 +
k11*k14*sigma1))
Free parameters:  XpY, Y, sigma1

Kinetic deficiency is positive.
Number of free parameters that can be solved:  1
Refer to the additional conditions displayed above.

Conservation laws:
dX/dt + dXD/dt + dXDYp/dt + dXT/dt + dXTYp/dt + dXp/dt + dXpY/dt = 0
-dX/dt - dXD/dt - dXT/dt - dXp/dt + dY/dt + dYp/dt = 0
Elapsed time is 2.731821 seconds.
```

Underlying algorithm of the code

Since CRITERIA is developed based on COMPILES, it adopts the same core functions of COMPILES, except that the translation step is replaced by the EFM–based translation. Several COMPILES functions are also modified to accommodate the enhancements implemented in CRITERIA. For a detailed description of the core functions used by COMPILES, the reader may refer to the supplementary material of [8].

As in COMPILES, CRITERIA first generates the finest independent decomposition of the inputted CRN and then performs translation for each subnetwork using `TranslationProgramWithDecomp`. Within this function, `indepDecomp` performs the decomposition, and `TranslationProgram` carries out the EFM-based translation by executing the three main steps of the procedure. The translated subnetworks are then merged into a single network by taking the union of their reactions. If successful, the function `GCRN` constructs the generalized CRN of the merged network. When multiple kinetic complexes are associated with a single stoichiometric complex, phantom edges are added in such a way that the resulting reaction graph is weakly reversible. The function also removes self-loop reactions in the kinetic-order CRN by identifying reactions in which the kinetic product equals the kinetic reactant and deleting the corresponding columns in the matrix of complexes. Throughout this process—from decomposition to translation and merging—reaction labels are carefully tracked to ensure accurate parameterization.

Finally, the function `analyticSolution` uses the outputs of `GCRN`—the matrices of complexes in the stoichiometric and kinetic-order CRNs—to compute the parametrization following the method in [1]. Since the kinetic-order CRN may contain multiple linkage classes, tree constants are computed separately for each class by collecting spanning trees per linkage class. This differs from COMPILES, which constructs a kinetic-order CRN for each independent subnetwork, resulting in only a single linkage class per subnetwork. For models with positive kinetic deficiency, additional equations are generated and displayed in the output. We note that the final parametrization reported by the package does not yet incorporate these additional conditions for such models.

SUPPLEMENTARY TABLE

Species	Meaning
$X1$	Gene 1
$X2$	Gene 2
$X3$	dCas9 with single guide RNA 1 complex (Cassg1)
$X4$	dCas9 with single guide RNA 2 complex (Cassg2)
$X5$	Cassg2 complex specifically bound to gene 1
$X6$	Cassg1 complex specifically bound to gene 2
$X7$	Cassg1 complex specifically and Cassg1 complex unspecifically bound to gene 2
$X8$	Cassg2 complex specifically and Cassg2 complex unspecifically bound to gene 1
$X9$	Cassg2 complex specifically and Cassg1 complex unspecifically bound to gene 1
$X10$	Cassg1 complex specifically and Cassg2 complex unspecifically bound to gene 2
$X11$	single guide RNA 1
$X12$	single guide RNA 2
$X13$	dCas9 complex
$X14$	Cassg1 complex unspecifically bound to gene 1
$X15$	Cassg2 complex unspecifically bound to gene 1
$X16$	Cassg1 complex unspecifically bound to gene 2
$X17$	Cassg2 complex unspecifically bound to gene 2

Table A. The species in the full CRISPRi toggle switch model in Fig 3a in the main text.

SUPPLEMENTARY FIGURES

We present in this subsection selected reaction networks corresponding to the biochemical models listed in Table 2 of the main text.

MAPK Model (Model Q)

Model Description

The MAPK network consists of a substrate protein X undergoing sequential phosphorylation by a kinase K and dephosphorylation by a phosphatase M. Each step in the process involves temporary binding between the protein and an enzyme, and can go forward or backward, making the signaling carefully controlled and flexible [9].

Original Network

$$X + K \underset{k_2}{\overset{k_1}{\rightleftarrows}} XK \xrightarrow{k_3} X_p + K \underset{k_5}{\overset{k_4}{\rightleftarrows}} X_pK \xrightarrow{k_6} X_{pp} + K$$

$$X_{pp} + M \underset{k_8}{\overset{k_7}{\rightleftarrows}} X_{pp}M \xrightarrow{k_9} X_pM \underset{k_{11}}{\overset{k_{10}}{\rightleftarrows}} X_p + M$$

$$X_p + M \underset{k_{13}}{\overset{k_{12}}{\rightleftarrows}} X_p^*M \xrightarrow{k_{14}} XM \underset{k_{16}}{\overset{k_{15}}{\rightleftarrows}} X + M$$

Translated Network

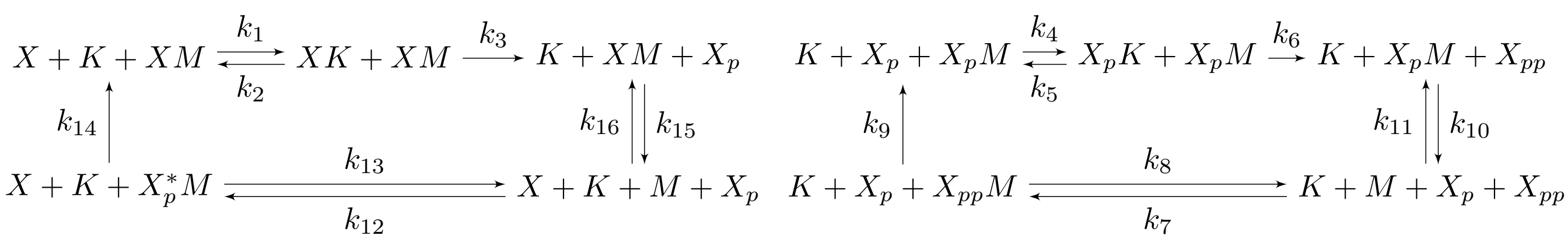

Zigzag Model of Plant-Pathogen Interactions (Model T)

Model Description

In the network below, we make the following assignment of species: X_1 = PAMP, X_2 = PRR, X_3 = PRR*, X_4 = Callose, $X_5 = \mathrm{E_{int}}$, X_6 = R, X_7 = R*, $X_8 = \mathrm{Pathogen_{bulk}}$, X_9 = Pathogen, X_{10} = E, X_{11} = F, X_12 = EF, and X_{13} = FCallose. The CRN is taken from [4].

This network is a conceptual model used to describe the layered immune response in plants. It captures the dynamic interplay between pathogen-associated molecular patterns (PAMPs), pattern recognition receptors (PRRs), resistance proteins, effectors, and immune responses such as callose deposition [10]. The reactions reflect how plants detect presence of pathogen, activate defense responses, and how pathogens attempt to suppress these defenses.

Original Network

$$X_1 + X_2 \underset{k_2}{\overset{k_1}{\rightleftarrows}} X_3 \qquad X_9 \xrightarrow{k_9} X_1 + X_9 \qquad X_7 + X_9 \xrightarrow{k_{15}} X_7$$

$$X_3 \xrightarrow{k_3} X_3 + X_4 \qquad X_9 \xrightarrow{k_{10}} X_{10} + X_9 \qquad X_4 + X_9 \xrightarrow{k_{16}} X_4$$

$$X_4 \xrightarrow{k_4} \emptyset \qquad X_1 \xrightarrow{k_{11}} \emptyset \qquad X_{10} + X_{11} \underset{k_{18}}{\overset{k_{17}}{\rightleftarrows}} X_{12}$$

$$X_4 + X_5 \xrightarrow{k_5} X_5 \qquad X_5 \xrightarrow{k_{12}} \emptyset \qquad X_{12} \xrightarrow{k_{19}} X_5 + X_{11}$$

$$X_5 + X_6 \underset{k_7}{\overset{k_6}{\rightleftarrows}} X_7 \qquad X_9 \xrightarrow{k_{13}} \emptyset \qquad X_4 + X_{11} \underset{k_{21}}{\overset{k_{20}}{\rightleftarrows}} X_{13}$$

$$X_8 \xrightarrow{k_8} X_8 + X_9 \qquad X_{10} \xrightarrow{k_{14}} \emptyset$$

Translated Network

$$X_1 + X_2 \underset{k_2}{\overset{k_1}{\rightleftarrows}} X_3 \underset{k_4, k_5}{\overset{k_3}{\rightleftarrows}} X_3 + X_4 \qquad X_5 + X_6 \underset{k_7}{\overset{k_6}{\rightleftarrows}} X_7 \qquad X_8 \underset{k_{13}, k_{15}, k_{16}}{\overset{k_8}{\rightleftarrows}} X_8 + X_9$$

$$X_9 \underset{k_{11}}{\overset{k_9}{\rightleftarrows}} X_1 + X_9 \qquad X_9 + X_{11} \underset{k_{14}}{\overset{k_{10}}{\rightleftarrows}} X_9 + X_{10} + X_{11} \qquad X_{11} + X_{14} \underset{k_{21}}{\overset{k_{20}}{\rightleftarrows}} X_{13}$$

$$X_9 + X_{10} + X_{11} \underset{k_{18}}{\overset{k_{17}}{\rightleftarrows}} X_9 + X_{12} \qquad X_9 + X_{12} \xrightarrow{k_{12}} X_5 + X_9 + X_{11} \qquad X_5 + X_9 + X_{11} \xrightarrow{k_{12}} X_9 + X_{11}$$

WNT Signaling Pathway (Model W)

Model Description

This network is a key system in regulating cell behavior during development and in adult tissues. It captures the dynamic interactions that determine whether the pathway is in an "off" state—where β-catenin is degraded by a destruction complex—or in an "on" state — leading to β-catenin accumulation [11]. The role of β-catenin is crucial as it serves as a messenger that links external signals to gene expression in the nucleus. The model reflects how molecules interact across different cellular compartments and includes both degradation and synthesis mechanisms, with complex nonlinear behaviors driven by the presence or absence of external signals [11].

Original Network

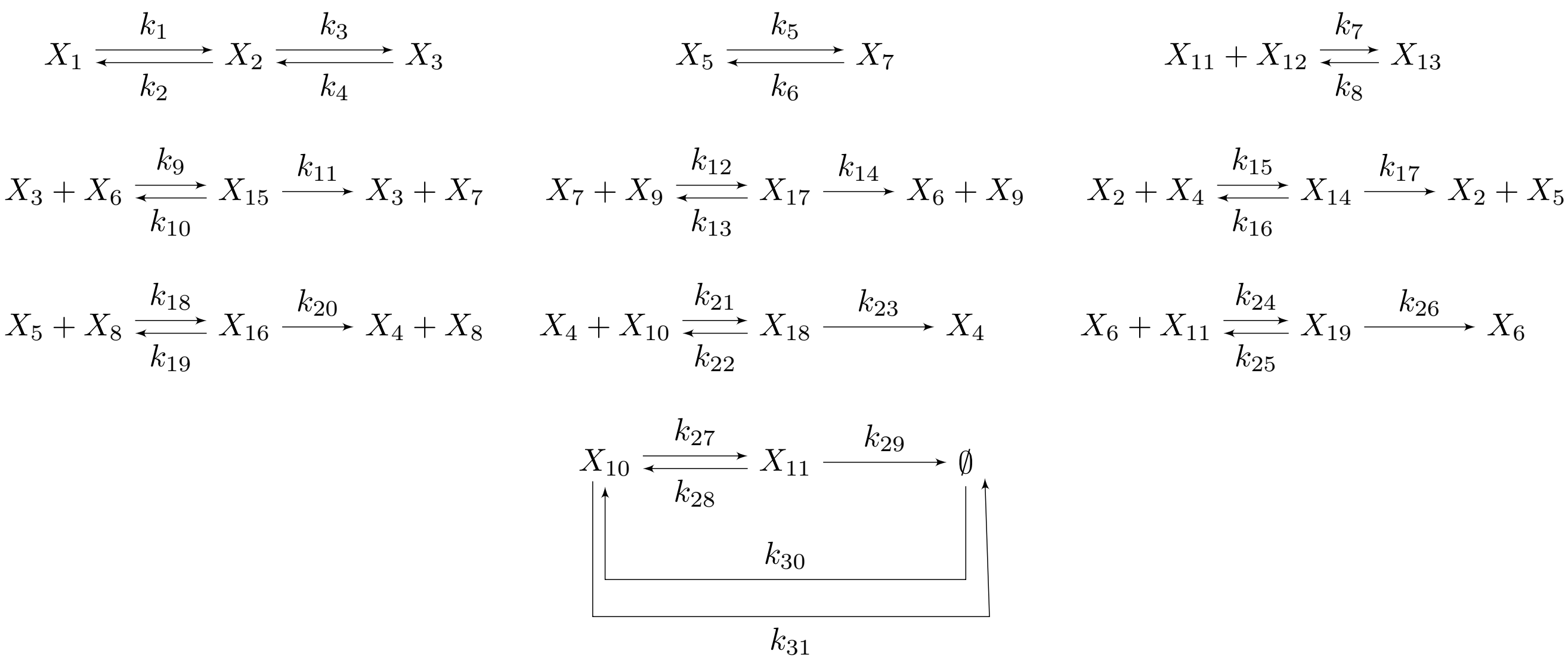

Translated Network

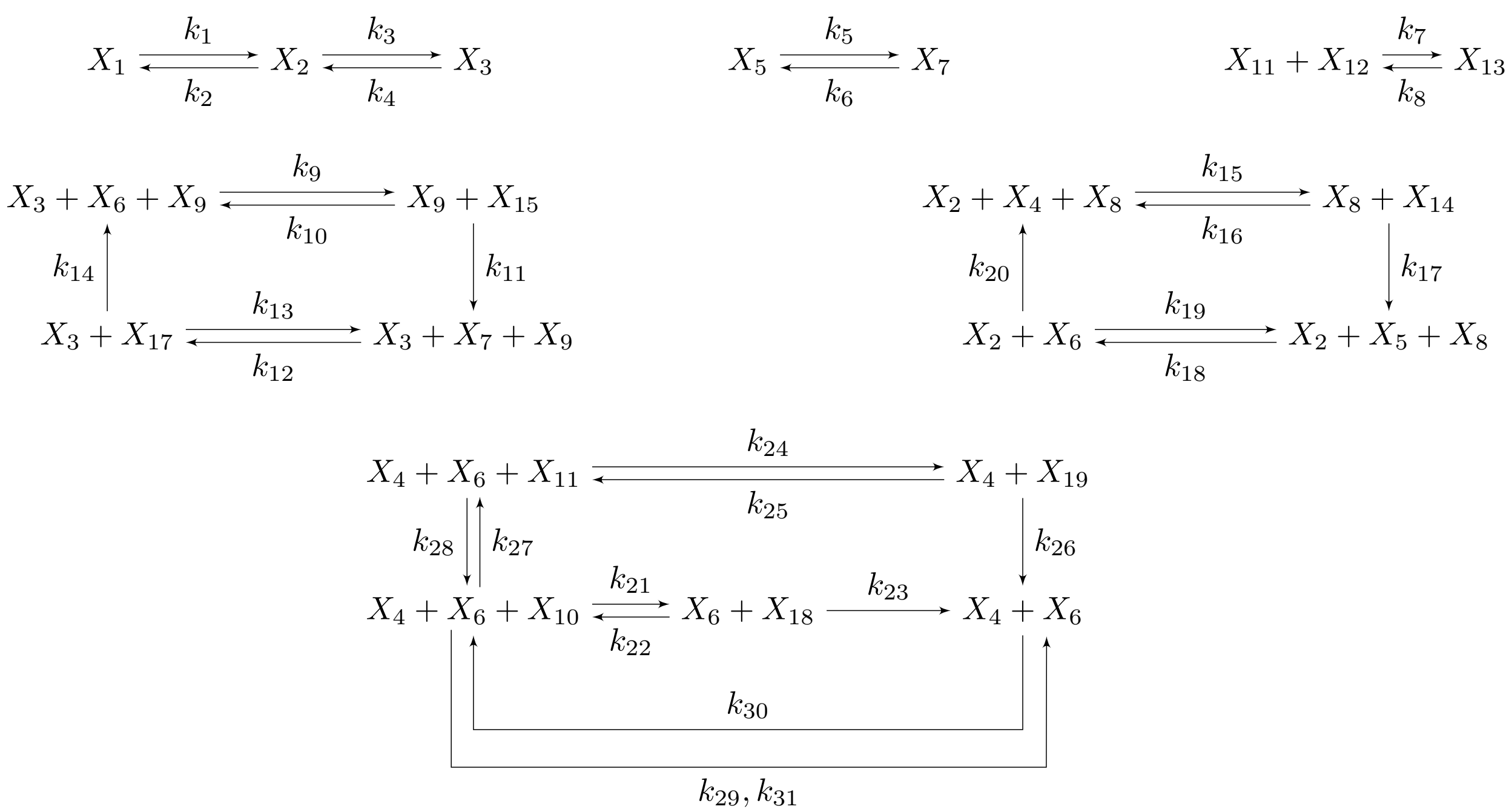

[1] C. P. Matthew Johnston, Stefan Müller, A deficiency-based approach to parametrizing positive equilibria of biochemical reaction systems, Bull Math Biol. **81**, 1143 (2019).
[2] N. Fortun, E. Mendoza, L. Razon, and A. Lao, A deficiency zero theorem for a class of power-law kinetic systems with non-reactant-determined interactions, MATCH Commun. Math. Comput. Chem. **81**, 621 (2019).
[3] I. Klapper, D. Szyld, and K. Zhao, *Metabolic networks, elementary flux modes, and polyhedral cones* (Society for Industrial and Applied Mathematics, Philadelphia, 2021).
[4] M. Johnston and E. Burton, Computing weakly reversible deficiency zero network translations using elementary flux modes, Bull Math Biol. **81**, 1613 (2019).
[5] M. Feinberg, *Foundations of Chemical Reaction Network Theory* (Springer International Publishing, 2019).
[6] E. Feliu and C. Wiuf, Variable elimination in chemical reaction networks with mass-action kinetics, SIAM Journal on Applied Mathematics **72**, 959 (2012).
[7] A. Mahdi, A. Ferragut, C. Valls, and C. Wiuf, Conservation laws in biochemical reaction networks, SIAM Journal on Applied Dynamical Systems **16**, 2213 (2017).
[8] B. Hernandez, P. V. Lubenia, M. Johnston, and J. K. Kim, A framework for deriving analytic steady states of biochemical reaction networks, PLoS Comput Biol. **19**, e1011039 (2023).
[9] N. I. Markevich, J. B. Hoek, and B. N. Kholodenko, Signaling switches and bistability arising from multisite phosphorylation in protein kinase cascades, J. Cell. Biol. **164**, 353–359 (2004).
[10] L. Pritchard and P. R. J. Birch, The zigzag model of plant–microbe interactions: is it time to move on?, Molecular Plant Pathology **15**, 865 (2014).
[11] E. Gross, H. Harrington, Z. Rosen, and B. Sturmfels, Algebraic systems biology: A case study for the Wnt pathway, Bull. Math. Biol. **78**, 21–51 (2016).
