# Supplementary material for: CRITERIA: A network decomposition and elementary flux mode translation-based tool for computing equilibria of biochemical systems
Source: PLoS Comput Biol. 2026 Aug 11;22(8):e1014633. doi: 10.1371/journal.pcbi.1014633 (PMC13475990; doi:10.1371/journal.pcbi.1014633)
Supplement: Supplementary file 2 [file pcbi.1014633.s002.pdf]

CRITERIA: A network decomposition and elementary flux mode translation-based tool for computing equilibria of biochemical systems

This is a substantive work and is within the scope *PLOS Computational Biology*. The paper addresses a well-documented computational bottleneck in an existing, published framework (COMPILES, *PLoS Comput Biol* 2023 paper), extends the applicability class, and packages the result as open-source software.

Recommendation: Accepted with major revision

Comments:

- Page 13, lines 341-343: The correctness of the computed steady states is claimed by substituting the derived equilibria into the ODEs and observing zero derivatives. Given that the main contribution of this work is providing correct analytic equilibria, a more convincing validation is needed. Can the authors explicitly confirm that the ODE substitution check was performed for all 26 models? Moreover, a cross-check/comparison against COMPILES on the subset of cases where the COMPILES does work can provide a more robust validation.
- Page 15, line 384-386: The claim that X_pY can admit *exactly* two positive equilibria or none at all is attributed to the 2 sign changes of the cubic in X_pY. However, the Descartes' Rule of Signs gives an upper bound, not an exact count. The word "exactly," and the word "Equivalently" (in line 385) linking the sign rule to the equilibrium count, claim more than Descartes can deliver. The authors may consider reformulating the proof (one that's more exact and elementary): Observe that every term of the "cubic" in X_pY has a factor of X_pY. That is, $f(X_pY) = X_pY \cdot g(X_pY)$ with g being quadratic. Write $f(X_pY) = 0$, with
$$g(X_pY) = AX_pY + BX_pY + C$$
and discarding the case where $X_pY = 0$ (since a positive equilibrium requires $X_pY > 0$), the positive equilibria are exactly the positive roots of the quadratic g. This leads to a stronger result than the current proof provides. In particular, when $B^2 - 4AC > 0$, an explicit parameter condition for the existence of two distinct positive equilibria is obtained. The existence of the two-equilibria case is then attributed to the explicit instance in Fig 2 (upper curve), not to the sign rule.
- EnvZ–OmpR is Model L, which has kinetic deficiency 1 (Table 2). The package output (SI Text, p. 13) prints a separate side condition, `Eq 1: 1 = (k1*k3*k12)/(k2*sigma1*(k4 + k5))` and S1 Text states explicitly (p. 13, final paragraph) that "the final parametrization reported by the package does not yet incorporate these additional conditions." The multistationarity proof, however, treats $\alpha_1, \ldots, \alpha_7$ as free parameters. Does imposing `Eq 1` affect the multistationarity conclusion?
- S1 Theorem 1 (preservation of weak reversibility and zero deficiency under merging of independent translated subnetworks) is the theoretical foundation of the merge-then-solve approach. The main text introduces merging (pp. 8–9, lines 255–262) without stating or citing this result. The theorem should be stated (at least informally) in the main text at the merging step, with the proof referenced to the supplement.
- All runtimes appear to be single measurements on a MacBook Air (Apple M2, 8GB RAM). Symbolic MATLAB runtimes are highly variable and memory-sensitive. Reporting mean ± standard deviation over repeated runs, and testing whether the largest models (i.e., X, Y, Z) are memory-bound, would strengthen the efficiency claim.

Minor/clerical comments:

- References [5] and [23] are identical: Johnston MD, Burton E. *Computing weakly reversible deficiency zero network translations using elementary flux modes*. Bull Math Biol.
- Pages 8-9, lines 238-251: The kinetic-order for N_1 is labelled N'_{1K} at lines 238/241/250. Is there a typo error in N'_{2K} in line 248? Fig 1b uses N'_{2K} for a separate N_2 branch.
- Page 19, lines 480-481: "(see Fig 3)" for runtime evidence but the data are in Table 3.
- Pages 19-20: Figures 4 and 5 are nearly identical. Consider merging them side-by-side as COMPILES-vs-CRITERIA diagram.
- Spelling: "derivering" (p. 2, l. 61), "woth noting" (p. 9, l. 284), "produding" (p. 16, l. 417), "equlibrium" (p. 17, l. 434), "authophosphorylation" (p. 13, l. 352), "CRIPSRi" (Fig 3 title, p. 17). There could be more so a full proofreading is needed.
- There are two Theorem 1's. The supplement's main result is "Theorem 1," but the Methods also has a "Theorem 1" (from [25]), and the supplement refers back to "Theorem 2 in the main text." Renumbering the supplementary results ("Theorem S1," etc.) would remove the confusion.
