# Supplementary material for: CRITERIA: A network decomposition and elementary flux mode translation-based tool for computing equilibria of biochemical systems
Source: PLoS Comput Biol. 2026 Aug 11;22(8):e1014633. doi: 10.1371/journal.pcbi.1014633 (PMC13475990; doi:10.1371/journal.pcbi.1014633)
Supplement: Supplementary file 3 [file pcbi.1014633.s003.pdf]

Review Comments for
"CRITERIA: A network decomposition and elementary flux mode translation-based tool for computing equilibria of biochemical systems"
submitted to PLOS Computational Biology

Recommendation: *Major Revision*
I recommend that the paper be accepted after the authors have addressed the comments I listed below.

Summary: This paper introduces introduces CRITERIA, a MATLAB-based computational framework for deriving analytic positive equilibria (parametrized steady states) of biochemical reaction networks modeled under mass-action kinetics, with a properly working MATLAB implementation available on GitHub. This work is presented as an improvement of the authors' prior framework COMPILES (Hernandez et al., PLoS Comput Biol. 2023;19(4):e1011039), which relies on the concept of network translation and the theory of generalized chemical reaction networks. The authors provide the three main improvments:

(1) This resolves the issue of prohibitively large computation of COMPILES when generating candidate translated networks, by replacing the exhaustive search with a more efficient approach based on elementary flux modes (EFMs) introduced by Johnston and Burton.

(2) This resolves the inter-dependencies of in derived steady-state parametrizations (e.g., XD and X_pY equations on page 3), by computing steady states on a single unified translated network rather than solving subnetworks independently.

(3) This extends the applicability of COMPILES from the class of CRNs with kinetic deficiency zero to a broader class of CRNs (c.f. Theorem 2 - Case 2 in the manuscript).

This work certainly provides a well justified computational framework for computing steady states of a system of ODEs arising from biochemical reaction networks under the mass-action kinetics assumption. Although there seems to be no mathematically novel results, the framework is useful and practical for researchers in the field of chemical reaction network theory and systems biology.

In summary, this paper is a nice contribution to the field of studying steady states of biochemical reaction networks. However, I have some major comments to be addressed before the paper can be accepted to PLOS Computational Biology, and I also have some minor comments that the authors should consider. Overall, I recommend a major revision.

Major Comments

1. The presentation of the main framework, CRITERIA, can be improved. The authors explain the main idea using a running example on page 4 with Steps 1–3. However, they do not quite

correspond to Steps 1–4 of Figure 1, which is also intended to illustrate the main framework. Also, Figure 1 uses a different (more complex) example on page 7. I think they should share the same example and be more consistent with explicit Step numbers.

It would be significantly better if the authors can revise the Results section and Figure 1 to make them more consistent. Also, they should refer to each part of Figure 1 more explicitly in the text. Currently, only Panels (a) and (b) of Figure 1 are referred to few times in the text. In this way, the readers could follow the main framework with the Results section and Figure 1 together, which would make the presentation much clearer and more intuitive.

2. Regarding the 3rd improvement of CRITERIA about the applicability to CRNs with positive kinetic deficiency, the authors provide many working examples in Table 2 and relevant propoistion, Theorem 2 - Case 2. However, it is not clear why the preivous framework COMPILES cannot be applied to these examples, while CRITERIA can be applied. The authors should provide more detailed discussion on this point, and if possible, provide some examples of CRNs with positive kinetic deficiency that are not covered by COMPILES but are covered by CRITERIA. This would help the readers to better understand the significance of this improvement.

   Note that the 1st and 2nd improvements (and even the sources of these improvements) are well justified and clear to me.

3. The key reference of this work is the authors' previous work COMPILES (Hernandez et al., PLoS Comput Biol. 2023;19(4):e1011039), while in a broader perspective, this work concerns parametrization of steady states of biochemical reaction networks, which is a fundamental problem in chemical reaction network theory. However, in Introduction, the authors only briefly mention COMPILES and do not discuss other related works in the field. I think it would be better if the authors provide a more comprehensive review of the literature on this topic. Please see, for example, the Introduction section of the paper (Bulletin of Mathematical Biology (2019) 81:1143–1172) by Johnston, Müller, and Pantea and the references therein for some examples of relevant works.

Minor Comments

1. Line 66: For consistency, the term "weak reversibility (WR)" should be revised as "being weakly reversible (WR)" as the term "deficiency zero" is used as an adjective in the same sentence.

2. Line 173: "elementary flux modes (EM) compatible" should be revised as "elementary flux modes compatible (EM-compatible)". This is already used by the authors in Line 781. It's a bit tricky as the term "elementary flux modes" is abbreviated as "EFMs" throughout the manuscript. I'd be happy to see any consistent, clear way to abbreviate the terms.

3. Algorithm 2 Bullet point 2: There is a minor quotation mark typo, which is common in Latex-generated PDF. The term ”active” (including the quotation marks) should be revised as “active”. There are more quotation mark typos in the manuscript, e.g., Line 246. Please check the manuscript for more quotation mark typos and fix them.

4. I suggest the authors to merge Figures 4 and 5 into a single figure with two panels, as it would be more efficient to see the similarities and differences between the two frameworks. Not just merging the two figures, but also revising the figures so that the readers can easily compare the two frameworks would be helpful.

5. Line 240: $k_3 P_1$ is the reaction rate for the reaction $G_1 + P_1 \to G_1$, which is obtained after network translation. It is correct. However, please add some explanation why that is the correct reacion rate and also refer the relevant part of Figure 1 or any other equations in the manuscript. It can be confusing to the readers who are not familiar with the concept of network translation and the theory of generalized chemical reaction networks.
