# Supplementary material for: CRITERIA: A network decomposition and elementary flux mode translation-based tool for computing equilibria of biochemical systems
Source: PLoS Comput Biol. 2026 Aug 11;22(8):e1014633. doi: 10.1371/journal.pcbi.1014633 (PMC13475990; doi:10.1371/journal.pcbi.1014633)
Supplement: Supplementary file 4 [file pcbi.1014633.s005.pdf]

CRITERIA: A network decomposition and elementary flux mode translation-based tool for computing equilibria of biochemical systems

Manuscript PCOMPBIOL-D-26-01008 R1 (revised version, 8 July 2026)

The authors have satisfactorily addressed the substantive comments from the first round of review. A few remaining minor errors (detailed below) should be corrected before the manuscript is ready for final acceptance.

- For the toy model of Example 1, with the ODEs given in Example 4, $\frac{db}{dt} = 0$ gives $k_2 a = k_3 b$, $\frac{da}{dt} = 0$ then reduces to $k_1 ab = k_5$, and $\frac{dc}{dt} = 0$ gives $c = \frac{k_1 ab}{k_4}$. Thus,

$$(a, b, c) = \left(\sqrt{\frac{k_3 k_5}{k_1 k_2}}, \sqrt{\frac{k_2 k_5}{k_1 k_3}}, \frac{k_5}{k_4} \right),$$

  whereas the manuscript gives $\left(\sqrt{\frac{k_3 k_5}{k_1 k_4}}, \sqrt{\frac{k_3 k_4}{k_1 k_5}}, \frac{k_3}{k_2} \right)$.

- The URL https://github.com/pvnlubenia (line 435) is a user profile, not the CHECKMATE repository; please link the repository directly.
- SI Table A caption refers to “Fig 6a in the main text”. There is no Fig. 6; it should be Fig. 3a.
