# Supplementary material for: CRITERIA: A network decomposition and elementary flux mode translation-based tool for computing equilibria of biochemical systems
Source: PLoS Comput Biol. 2026 Aug 11;22(8):e1014633. doi: 10.1371/journal.pcbi.1014633 (PMC13475990; doi:10.1371/journal.pcbi.1014633)
Supplement: Supplementary file 5 [file pcbi.1014633.s006.pdf]

Response Letter

Response to Reviewer's Comments
We thank the reviewers for their constructive comments and helpful suggestions for strengthening the presentation and quality of our manuscript. Below, we give our detailed responses to the reviewers' comments. The comments of the reviewers appear in **green** and our responses in **black**. The **blue**, **magenta**, and **orange** texts indicate the revisions in the main manuscript corresponding to the comments of Reviewer 1, 2, and 3, respectively.

Reviewer 1

Summary

This is a substantive work and is within the scope PLOS Computational Biology. The paper addresses a well-documented computational bottleneck in an existing, published framework (COMPILES, PLoS Comput Biol 2023 paper), extends the applicability class, and packages the result as open-source software.

We are grateful to the reviewer for this supportive and encouraging comment.

Comment 1

Page 13, lines 341-343: The correctness of the computed steady states is claimed by substituting the derived equilibria into the ODEs and observing zero derivatives. Given that the main contribution of this work is providing correct analytic equilibria, a more convincing validation is needed. Can the authors explicitly confirm that the ODE substitution check was performed for all 26 models? Moreover, a cross-check/comparison against COMPILES on the subset of cases where the COMPILES does work can provide a more robust validation.

To verify the correctness of the derived equilibria, we used MATLAB to plug in the expressions into the model's ODE system. The MATLAB file named *ModelName*_checking.m in the CRITERIA folder of our GitHub repository does the verification for each model. Kindly see Figure 1.

For instance, we check the correctness of the steady state parametrization of the EnvZ-OmpR model (Model L). See Figure 2 for the MATLAB script of the verification of Model L. Lines 11 to 19 contain the ODE system of the model. This is obtained by using CHECKMATE (https://github.com/pvnlubenia), which is a MATLAB code used to generate the ODE of a chemical reaction network with mass-action kinetics. Lines 24

to 30 contain the steady state parametrization as generated by CRITERIA. Since Model L has kinetic deficiency equal to one, line 21 shows the additional condition necessary for the final parametrization based on Case 2 of Theorem 2 in the main manuscript. Lines 33 to 41 is the checking part. Upon the running the code, it can be observed that time derivatives evaluate to zero. See Figure 3.

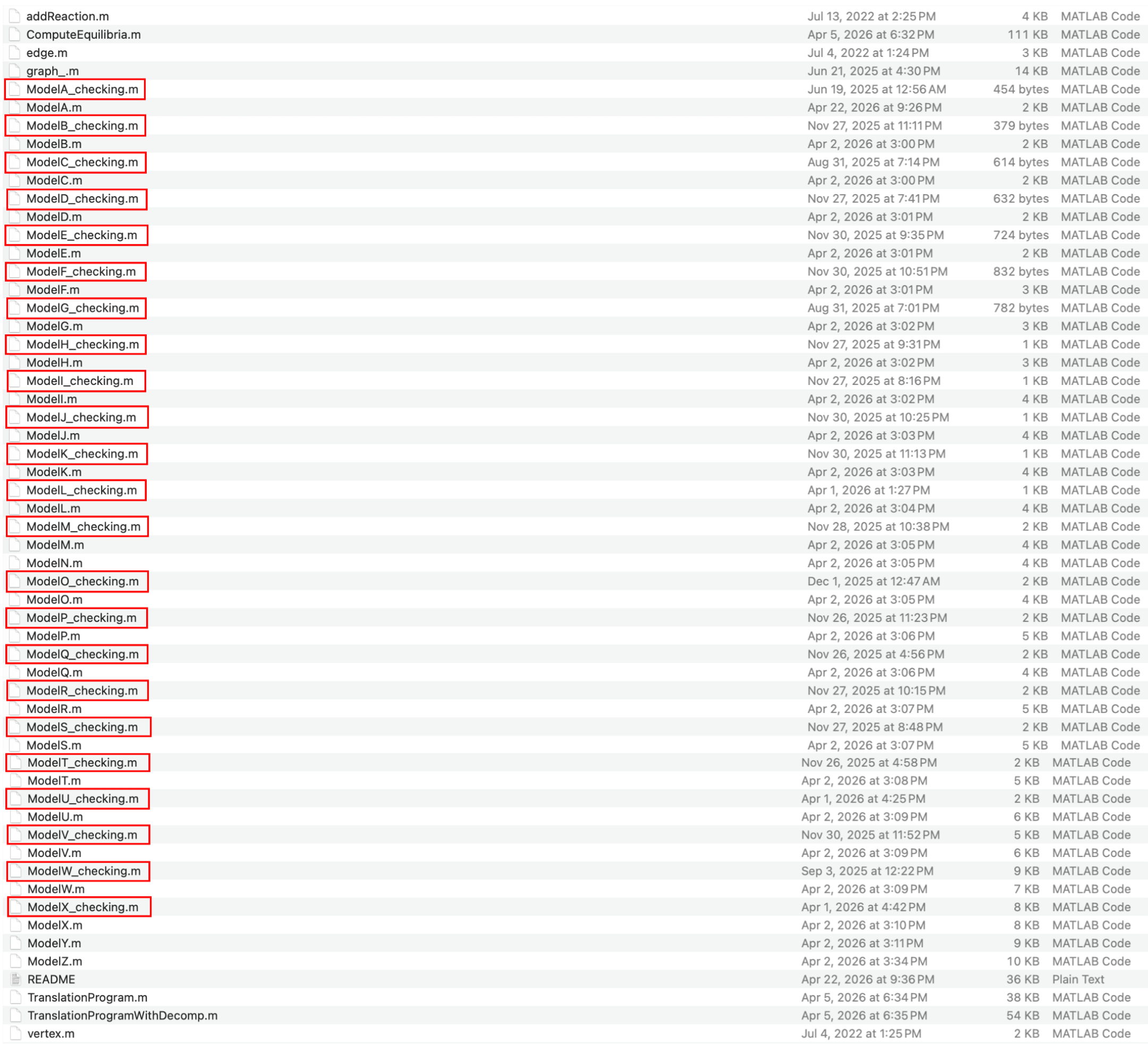

addReaction.m	Jul 13, 2022 at 2:25 PM	4 KB	MATLAB Code
ComputeEquilibria.m	Apr 5, 2026 at 6:32 PM	111 KB	MATLAB Code
edge.m	Jul 4, 2022 at 1:24 PM	3 KB	MATLAB Code
graph_.m	Jun 21, 2025 at 4:30 PM	14 KB	MATLAB Code
ModelA_checking.m	Jun 19, 2025 at 12:56 AM	454 bytes	MATLAB Code
ModelA.m	Apr 22, 2026 at 9:26 PM	2 KB	MATLAB Code
ModelB_checking.m	Nov 27, 2025 at 11:11 PM	379 bytes	MATLAB Code
ModelB.m	Apr 2, 2026 at 3:00 PM	2 KB	MATLAB Code
ModelC_checking.m	Aug 31, 2025 at 7:14 PM	614 bytes	MATLAB Code
ModelC.m	Apr 2, 2026 at 3:00 PM	2 KB	MATLAB Code
ModelD_checking.m	Nov 27, 2025 at 7:41 PM	632 bytes	MATLAB Code
ModelD.m	Apr 2, 2026 at 3:01 PM	2 KB	MATLAB Code
ModelE_checking.m	Nov 30, 2025 at 9:35 PM	724 bytes	MATLAB Code
ModelE.m	Apr 2, 2026 at 3:01 PM	2 KB	MATLAB Code
ModelF_checking.m	Nov 30, 2025 at 10:51 PM	832 bytes	MATLAB Code
ModelF.m	Apr 2, 2026 at 3:01 PM	3 KB	MATLAB Code
ModelG_checking.m	Aug 31, 2025 at 7:01 PM	782 bytes	MATLAB Code
ModelG.m	Apr 2, 2026 at 3:02 PM	3 KB	MATLAB Code
ModelH_checking.m	Nov 27, 2025 at 9:31 PM	1 KB	MATLAB Code
ModelH.m	Apr 2, 2026 at 3:02 PM	3 KB	MATLAB Code
ModelI_checking.m	Nov 27, 2025 at 8:16 PM	1 KB	MATLAB Code
ModelI.m	Apr 2, 2026 at 3:02 PM	4 KB	MATLAB Code
ModelJ_checking.m	Nov 30, 2025 at 10:25 PM	1 KB	MATLAB Code
ModelJ.m	Apr 2, 2026 at 3:03 PM	4 KB	MATLAB Code
ModelK_checking.m	Nov 30, 2025 at 11:13 PM	1 KB	MATLAB Code
ModelK.m	Apr 2, 2026 at 3:03 PM	4 KB	MATLAB Code
ModelL_checking.m	Apr 1, 2026 at 1:27 PM	1 KB	MATLAB Code
ModelL.m	Apr 2, 2026 at 3:04 PM	4 KB	MATLAB Code
ModelM_checking.m	Nov 28, 2025 at 10:38 PM	2 KB	MATLAB Code
ModelM.m	Apr 2, 2026 at 3:05 PM	4 KB	MATLAB Code
ModelN.m	Apr 2, 2026 at 3:05 PM	4 KB	MATLAB Code
ModelO_checking.m	Dec 1, 2025 at 12:47 AM	2 KB	MATLAB Code
ModelO.m	Apr 2, 2026 at 3:05 PM	4 KB	MATLAB Code
ModelP_checking.m	Nov 26, 2025 at 11:23 PM	2 KB	MATLAB Code
ModelP.m	Apr 2, 2026 at 3:06 PM	5 KB	MATLAB Code
ModelQ_checking.m	Nov 26, 2025 at 4:56 PM	2 KB	MATLAB Code
ModelQ.m	Apr 2, 2026 at 3:06 PM	4 KB	MATLAB Code
ModelR_checking.m	Nov 27, 2025 at 10:15 PM	2 KB	MATLAB Code
ModelR.m	Apr 2, 2026 at 3:07 PM	5 KB	MATLAB Code
ModelS_checking.m	Nov 27, 2025 at 8:48 PM	2 KB	MATLAB Code
ModelS.m	Apr 2, 2026 at 3:07 PM	5 KB	MATLAB Code
ModelT_checking.m	Nov 26, 2025 at 4:58 PM	2 KB	MATLAB Code
ModelT.m	Apr 2, 2026 at 3:08 PM	5 KB	MATLAB Code
ModelU_checking.m	Apr 1, 2026 at 4:25 PM	2 KB	MATLAB Code
ModelU.m	Apr 2, 2026 at 3:09 PM	6 KB	MATLAB Code
ModelV_checking.m	Nov 30, 2025 at 11:52 PM	5 KB	MATLAB Code
ModelV.m	Apr 2, 2026 at 3:09 PM	6 KB	MATLAB Code
ModelW_checking.m	Sep 3, 2025 at 12:22 PM	9 KB	MATLAB Code
ModelW.m	Apr 2, 2026 at 3:09 PM	7 KB	MATLAB Code
ModelX_checking.m	Apr 1, 2026 at 4:42 PM	8 KB	MATLAB Code
ModelX.m	Apr 2, 2026 at 3:10 PM	8 KB	MATLAB Code
ModelY.m	Apr 2, 2026 at 3:11 PM	9 KB	MATLAB Code
ModelZ.m	Apr 2, 2026 at 3:34 PM	10 KB	MATLAB Code
README	Apr 22, 2026 at 9:36 PM	36 KB	Plain Text
TranslationProgram.m	Apr 5, 2026 at 6:34 PM	38 KB	MATLAB Code
TranslationProgramWithDecomp.m	Apr 5, 2026 at 6:35 PM	54 KB	MATLAB Code
vertex.m	Jul 4, 2022 at 1:25 PM	2 KB	MATLAB Code

Figure 1: Files inside the CRITERIA folder.

There are no verification files for Models N, Y, and Z because of their complicated additional conditions as these are high kinetic deficiency models. In other words, it is difficult to solve for the sigmas using the additional conditions. Other models with positive kinetic deficiency, namely Models M, O, Q, R, S, and X, have been verified to have zero time derivatives when plugged by their respective steady state parametrizations.

Now, we compare the models which worked for both COMPILES and CRITERIA for a more robust validation. See Table 1 for the summary of results. There are 13 models which worked for both COMPILES and CRITERIA. Among these models, five of which have identical steady state parametrizations. Of the remaining eight models with differing steady state parametrizations, COMPILES fails to produce zero time derivatives in three

ModelL_checking.m

```
% Clear all variables
clear all
clc
clear

% Define the symbols
syms k1 k2 k3 k4 k5 k6 k7 k8 k9 k10 k11 k12 k13 k14
syms X XD XDYp XT XTYp Xp XpY Y Yp

% ODEs
odeX  = k1*XD - k3*X - k2*X + k8*XpY + k4*XT;
odeXD  = k2*X - k1*XD + k10*XDYp + k11*XDYp - k9*XD*Yp;
odeXDYp  = k9*XD*Yp - k11*XDYp - k10*XDYp;
odeXT  = k3*X - k4*XT - k5*XT + k13*XTYp + k14*XTYp - k12*XT*Yp;
odeXTYp  = k12*XT*Yp - k14*XTYp - k13*XTYp;
odeXp  = k7*XpY + k5*XT - k6*Xp*Y;
odeXpY  = k6*Xp*Y - k8*XpY - k7*XpY;
odeY  = k11*XDYp + k7*XpY + k14*XTYp - k6*Xp*Y;
odeYp  = k10*XDYp + k8*XpY + k13*XTYp - k9*XD*Yp - k12*XT*Yp;

sigma1 = k1*k3*k12/(k2*(k4+k5));

% Solution
X = (XpY*k8*(k4 + k5))/(k3*k5);
XD = (XpY*k2*k8*(k4 + k5))/(k1*k3*k5);
XDYp = (XpY*k8*k9*(k13 + k14))/(k9*k11*k13 + k9*k11*k14 + k10*k14*sigma1 + k11*k14*sigma1);
XT = (XpY*k8)/k5;
XTYp = (XpY*k8*sigma1*(k10 + k11))/(k9*k11*k13 + k9*k11*k14 + k10*k14*sigma1 + k11*k14*sigma1);
Xp = (XpY*(k7 + k8))/(Y*k6);
Yp = (k5*sigma1*(k10 + k11)*(k13 + k14))/(k12*(k9*k11*k13 + k9*k11*k14 + k10*k14*sigma1 + k11*k14*sigma1));

% Check
checkX  = simplify(subs(odeX))
checkXD  = simplify(subs(odeXD))
checkXDYp  = simplify(subs(odeXDYp))
checkXT  = simplify(subs(odeXT))
checkXTYp  = simplify(subs(odeXTYp))
checkXp  = simplify(subs(odeXp))
checkXpY  = simplify(subs(odeXpY))
checkY  = simplify(subs(odeY))
checkYp  = simplify(subs(odeYp))
```

Figure 2: Verification of Model L.

cases, whereas CRITERIA correctly yields zero time derivatives for these same models. This is another source of improvement for CRITERIA over COMPILES.

Lastly, it can be observed in Table 1 that it is possible to have different yet valid steady state parametrizations. Indeed, this is possible as can be seen in a simple example. Consider the following chemical reaction network below consisting of three reactions.

$$A + B \xrightarrow{k_1} 2B \qquad B \xrightarrow{k_2} C \qquad C \xrightarrow{k_3} A$$

Assuming mass-action kinetcs, its ODE system is given by

$$\begin{aligned} \frac{dA}{dt} &= -k_1 AB + k_3 C \\ \frac{dB}{dt} &= k_1 AB - k_2 B \\ \frac{dC}{dt} &= k_2 B - k_3 C \end{aligned} \quad .$$

Solving the steady state expressions, we set each time derivatives to zero. From the second equation, we have $A = \dfrac{k_2}{k_1}$. If we set C as a free parameter, we have $B = \dfrac{k_3 \sigma}{k_2}$. This gives

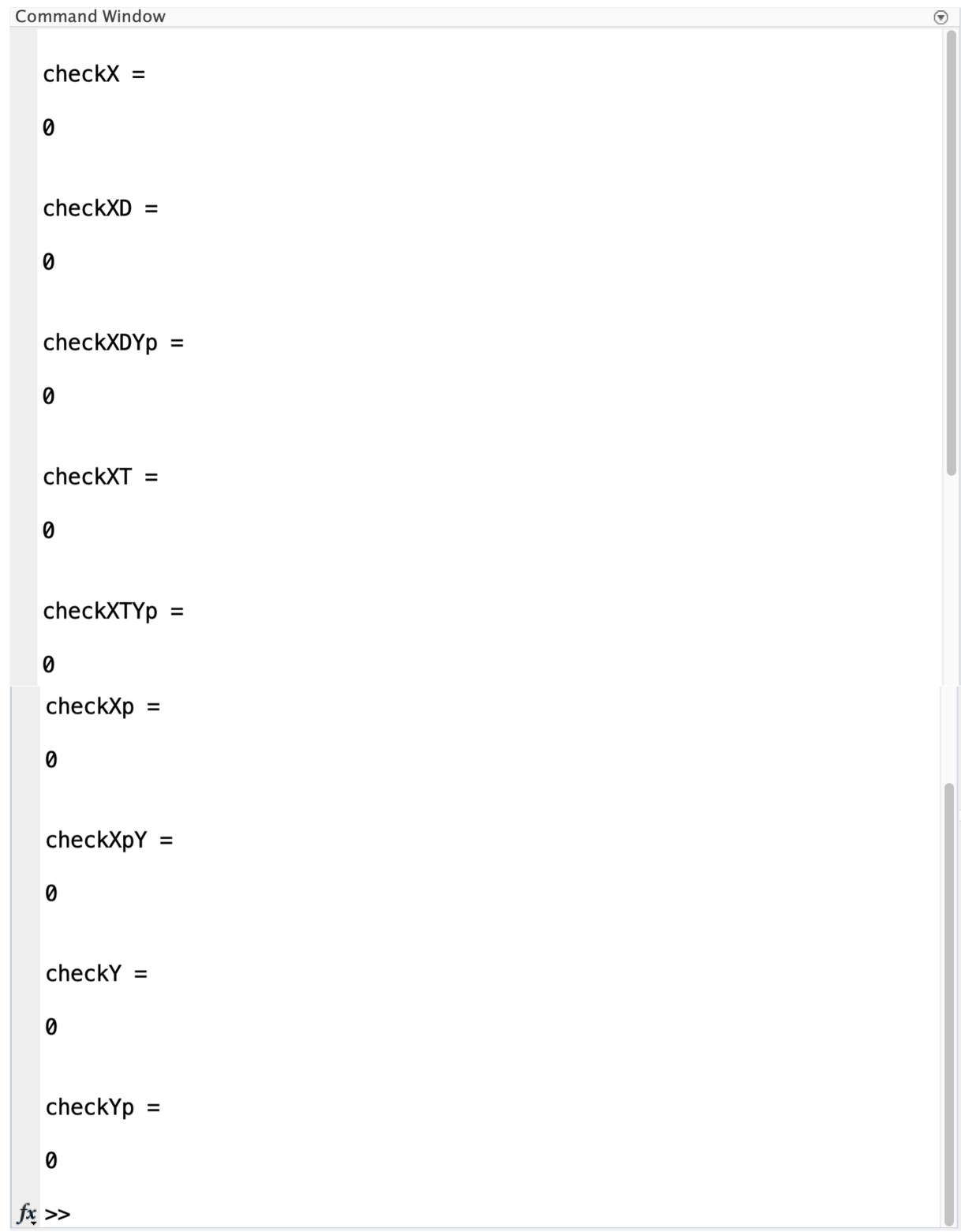

Figure 3: Result of the verification of Model L.

us the following state state parametrization:

$$(A, B, C) = \left(\frac{k_2}{k_1}, \frac{k_3\sigma}{k_2}, \sigma\right).$$

On the other hand, if we set B to be the free parameter, we have $C = \dfrac{k_2\sigma}{k_3}$. This gives another steady state parametrization:

$$(A^*, B^*, C^*) = \left(\frac{k_2}{k_1}, \sigma, \frac{k_2\sigma}{k_3}\right).$$

It can be verified that we get zero time derivatives upon plugging these parametrizations to the ODE system above. This shows that it is possible to have distinct yet valid steady state parametrization for the same model.

We revise as follows: (Page 15, lines 432-438)

We verify the correctness of the output produced by CRITERIA by substituting the derived equilibrium expressions into the model's ODE system using MATLAB. To get the mass-action ODE system of the models, we use CHECKMATE (https://github.com/pvnlubenia), which is a MATLAB code used to generate the ODE of a CRN with mass-action kinetics. We then plug in the output produced by CRITERIA into the ODE system. Indeed, the time derivatives evaluate to zero, confirming that the solutions are indeed valid equilibria.

Model	Is the output similar for both COMPILES and CRITERIA?	Do the output of COMPILES result to zero time derivatives?
Model A	Yes	-
Model B	Yes	-
Model C	No	No
Model D	Yes	-
Model E	Yes	-
Model G	Yes	-
Model I	No	Yes
Model J	No	Yes
Model K	No	Yes
Model P	No	No
Model Q	No	No
Model T	No	Yes
Model X	No	Yes

Table 1: Models which worked for both COMPILES and CRITERIA.

Comment 2

Page 15, line 384-386: The claim that X_pY can admit exactly two positive equilibria or none at all is attributed to the 2 sign changes of the cubic in X_pY. However, the Descartes' Rule of Signs gives an upper bound, not an exact count. The word "exactly," and the word "Equivalently" (in line 385) linking the sign rule to the equilibrium count, claim more than Descartes can deliver. The authors may consider reformulating the proof (one that's more exact and elementary): Observe that every term of the "cubic" in X_pY has a factor of X_pY. That is, $f(X_pY) = X_pY \cdot g(X_pY)$ with g being quadratic. Write $f(X_pY) = 0$, with $g(X_pY) = AX_pY + BX_pY + C$ and discarding the case where $X_pY = 0$ (since a positive equilibrium requires $X_pY > 0$), the positive equilibria are exactly the positive roots of the quadratic g. This leads to a stronger result than the current proof provides. In particular, when $B^2 - 4AC = 0$, an explicit parameter condition for the existence of two distinct positive equilibria is obtained. The existence of the two-equilibria case is then attributed to the explicit instance in Fig 2 (upper curve), not to the sign rule.

We thank the reviewer for his/her very insightful suggestion. We adopted it in the main manuscript with further enhancements for a stronger proof on our claim. We decided to retain our proof in the Supplementary Information because the signs of the coefficients are used to argue that the roots of the quadratic g are distinct and positive.

To avoid confusion and ambiguity, we revised the wording and explanation of our conclusion using Descartes' Rule of Signs.

The revisions are given as follows:

(Page 18, lines 491-509)

The left hand side of the equation is a cubic polynomial in X_pY with two sign changes indicating that the equation can either have two or zero positive real solutions by Descartes' Rule of Signs. Refer to the Supplementary Information for the proof. This means that X_pY can admit either two positive equilibria or none at all, implying that the system has the capacity for multistationarity. In other words, the system can have two positive equilibria for a particular choice of rate constants.

Alternatively, observe that the cubic expression in Eq (7) has a common factor X_pY. We can then write the cubic polynomial as $f(X_pY) = X_pY \cdot g(X_pY)$, with g being quadratic. Setting $f(X_pY) = 0$, we have $g(X_pY) = A(X_pY)^2 + BX_pY + C = 0$, where

$$\begin{aligned} A &= \gamma_1\alpha_6\gamma_2 - \gamma_1^2\alpha_6 \\ B &= 2T_1\gamma_1\alpha_6 - T_1\alpha_6\gamma_2 + \alpha_6^2 - \gamma_1\alpha_6\alpha_7 + \gamma_1\alpha_6T_2 \\ C &= T_1\alpha_6\alpha_7 - T_1^2\alpha_6 - T_1\alpha_6T_2. \end{aligned}$$

We discard the case when $X_pY = 0$ since we are interested in looking at the positive equilibrium. When the discriminant $B^2 - 4AC$ of g is positive, an explicit parameter condition for the existence of two distinct equilibria is obtained. Suppose $B^2 - 4AC > 0$. From the Supplementary Information, it has been shown that $A < 0$, $B > 0$, and $C < 0$. We can infer from the Vieta's formulas that the product of the roots of g is $\frac{C}{A} > 0$ so that either both roots are positive or both of them are negative. By the quadratic formula, we have

$$x_1 = \frac{-B}{2A} + \frac{\sqrt{B^2 - 4AC}}{2A} \qquad x_2 = \frac{-B}{2A} - \frac{\sqrt{B^2 - 4AC}}{2A}$$

Note that $\frac{-B}{2A} > 0$ and $\frac{\sqrt{B^2 - 4AC}}{2A} < 0$. So, we have $x_2 > 0$ implying that $x_1 > 0$. Under the $B^2 - 4AC > 0$ assumption, these two positive roots correspond to the two distinct and positive equilibria of the system.

(Page 18, lines 513-515)

In the context of the EnvZ-OmpR system, the possibility for the existence of two positive equilibria or none at all provides a mechanistic explanation for how the EnvZ–OmpR pathway can implement threshold-based osmotic sensing.

Comment 3

EnvZ–OmpR is Model L, which has kinetic deficiency 1 (Table 2). The package output (SI Text, p. 13) prints a separate side condition, `Eq 1: 1 = (k1*k3*k12)/(k2*sigma1*(k4 + k5))|` and S1 Text states explicitly (p. 13, final paragraph) that "the final parametrization reported by the package does not yet incorporate these additional conditions." The multistationarity proof, however, treats $\alpha_1, \ldots, \alpha_7$ as free parameters. Does imposing Eq 1 affect the multistationarity conclusion?

Yes, the final parametrization reported by CRITERIA does not yet incorporate the additional conditions when the model has positive kinetic deficiency. This is also the reason why we also displayed the additional conditions as a separate output from the obtained parametrization.

In the multistationarity proof of the EnvZ-OmpR model, the condition *Eq 1: 1 = (k1*k3*k12)/(k2*sigma1*(k4 + k5))* was incorporated in the parametrization by solving *sigma1* in the additional condition in terms of the rate constants and plugging the resulting expression to the output of CRITERIA. As can be observed in the parametrization displayed in the main manuscript (Pages 16-17, lines 463-464), *sigma1* no longer appears in the expressions.

Furthermore, the proof does not treat $a_1, \ldots, \alpha_7$ as free parameters. Rather, they are products and/or sums of rate constants. They are used to simplify the expressions and facilitate our computations. For instance, the steady state expression for the species X is given by $\dfrac{X_p Y k_8(k_4 + k_5)}{k_3 k_5}$. In the main manuscript, we let $\alpha_1 := \dfrac{k_8(k_4 + k_5)}{k_3 k_5}$ so that we have $X = \alpha_1 X_p Y$ as written in the main manuscript. The same is true for $\alpha_2, \alpha_3, \alpha_4, \alpha_5, \alpha_6$, and α_7.

The additional conditions are necessary to obtain a more accurate steady state parametrization for models with positive kinetic deficiency.

Comment 4

S1 Theorem 1 (preservation of weak reversibility and zero deficiency under merging of independent translated subnetworks) is the theoretical foundation of the merge-then-solve approach. The main text introduces merging (pp. 8–9, lines 255–262) without stating or citing this result. The theorem should be stated (at least informally) in the main text at the merging step, with the proof referenced to the supplement.

We thank the reviewer for this pointing this out. The revision is given as follows:

(Pages 9-10, lines 298-302)

The next step is to merge the subnetworks into a single unified network instead of treating each subnetwork as an individual piece. This step is supported by Theorem S1 in the Supporting Information, stating that the preservation of weak reversibility and zero deficiency under merging of independent translated subnetworks possessing these properties is guaranteed.

Comment 5

All runtimes appear to be single measurements on a MacBook Air (Apple M2, 8GB RAM). Symbolic MATLAB runtimes are highly variable and memory-sensitive. Reporting mean $\pm$ standard deviation over repeated runs, and testing whether the largest models (i.e., X, Y, Z) are memory-bound, would strengthen the efficiency claim.

We thank the revewer for his/her suggestion. In our original submission, Table 3 reported only the mean runtime obtained from five runs. Following your suggestion, we now also report the standard deviation across these runs. Furthermore, we present all measurements to three decimal places instead of four to ensure that the table fits in the page. The revised results are presented in Table 3.

To address the question of whether the largest models are memory-bound, we reran TOWARDZ on Model Z while monitoring MATLAB's memory usage throughout the computation. See Figure 4 for the statistics. The peak real memory consumption was 209 MB, which is relatively small compared with the 8 GB of available system memory of the machine. This observation suggests that the computation is unlikely to be memory-bound and that the failure of TOWARDZ to generate a valid translation within the prescribed maximum runtime is not caused by insufficient RAM. Rather, its inability to obtain the desired output is more likely attributable to the computational complexity of the translation search process.

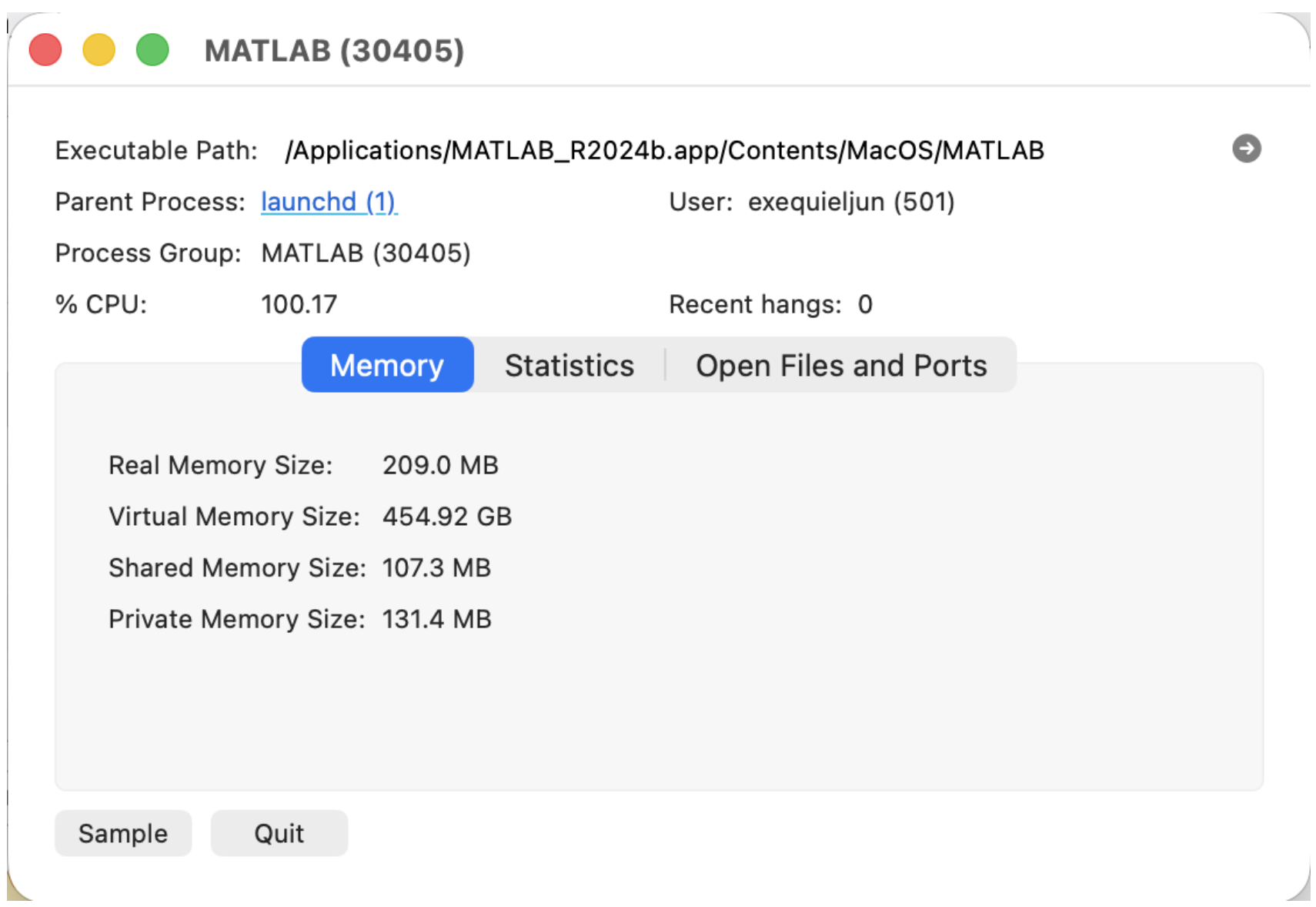

Figure 4: Memory usage of MATLAB during the execution of TOWARDZ on Model Z.

The revisions are given as follows:

(Page 12, lines 369-375)

For our computational experiments, we perform five independent runs, and report the mean and standard deviation of the resulting runtimes. In the first experiment, we compare the runtime performance of the EFM-based network translation, which we call REWARDZ (tRanslation via Elementary flux modes towards WeAkly Reversible Deficiency Zero networks), with TOWARDZ (TranslatiOn toward WeAkly Reversible and Deficiency Zero networks), the translation method used in COMPILES.

(Page 15, lines 406-407)

Moreover, we repeat the experiment five times, and the results are reported as the mean $\pm$ standard deviation across these runs.

(Page 12, lines 381-388)

It is important to note that the inability of TOWARDZ to generate the desired output within the prescribed maximum runtime is not caused by insufficient RAM. We monitored the memory usage of MATLAB throughout the computation for Model Z and observed that the peak real memory consumption is 209 MB which is relatively small compared with the 8 GB of available system memory of the machine. This suggests that its failure to obtain a valid translation is more likely attributable to the computational complexity of the translation search process.

Comments 6-11

6. References [5] and [23] are identical: Johnston MD, Burton E. Computing weakly reversible deficiency zero network translations using elementary flux modes. Bull Math Biol.
7. Pages 8-9, lines 238-251: The kinetic-order for N_1 is labelled N'_{1K} at lines 238/241/250. Is there a typo error in N'_{2K} in line 248? Fig 1b uses N'_{2K} for a separate N_2 branch.
8. Page 19, lines 480-481: "(see Fig 3)" for runtime evidence but the data are in Table 3.
9. Pages 19-20: Figures 4 and 5 are nearly identical. Consider merging them side-by-side as COMPILES-vs-CRITERIA diagram.
10. Spelling: "derivering" (p. 2, l. 61), "woth noting" (p. 9, l. 284), "produding" (p. 16, l. 417), "equlibrium" (p. 17, l. 434), "authophosphorylation" (p. 13, l. 352), "CRIPSRi" (Fig 3 title, p. 17). There could be more so a full proofreading is needed.
11. There are two Theorem 1's. The supplement's main result is "Theorem 1," but the Methods also has a "Theorem 1" (from [25]), and the supplement refers back to "Theorem 2 in the main text." Renumbering the supplementary results ("Theorem S1," etc.) would remove the confusion.

<u>Comment 6</u>

Reference [23] appeared in Table 2 as the citation for Models Q and T. So, we delete this duplicate reference.

We revise as follows:

(Page 12)

Model Q: MAPK [5]

Model T: Zigzag Model of Plant-Pathogen Interactions [5]

(Pages 32-33)

5. Johnston MD, Burton E. Computing weakly reversible deficiency zero network translations using elementary flux modes Bull Math Biol. 2019;81:1613–1644.

23. Hong H, Hernandez BS, Kim J, Kim JK. Computational translation framework identifies biochemical reaction networks with special topologies and their long-term dynamics. SIAM J Appl Math. 2023.

Comment 7

We revise as follows: (Page 9, lines 291-292)

This construction yields the kinetic-order CRN $\mathcal{N}'_{1_K}$, which is both WR and DZ ($\tilde{\delta} = \tilde{n} - \tilde{l} - \tilde{s} = 3 - 1 - 2 = 0$).

Comment 8

We revise as follows: (Page 21, lines 602-603)

We were able to show that CRITERIA indeed outperforms COMPILES, particularly for large reaction networks, in terms of computational runtime (see Table 3).

Comment 9

We thank the reviewer for his/her helpful suggestion. We combined the original Figures 4 and 5 into a single figure that presents the two computational frameworks in a vertical side-by-side layout, facilitating a direct comparison between them. The revised figure is now presented as Figure 4.

We revise as follows:

(Page 21, line 577)

(see Fig 4 and its accompanying discussion)

(Page 21, lines 587-588)

(see Fig 4 for the side-by-side comparison of the schematic diagrams of both frameworks)

(Page 22)

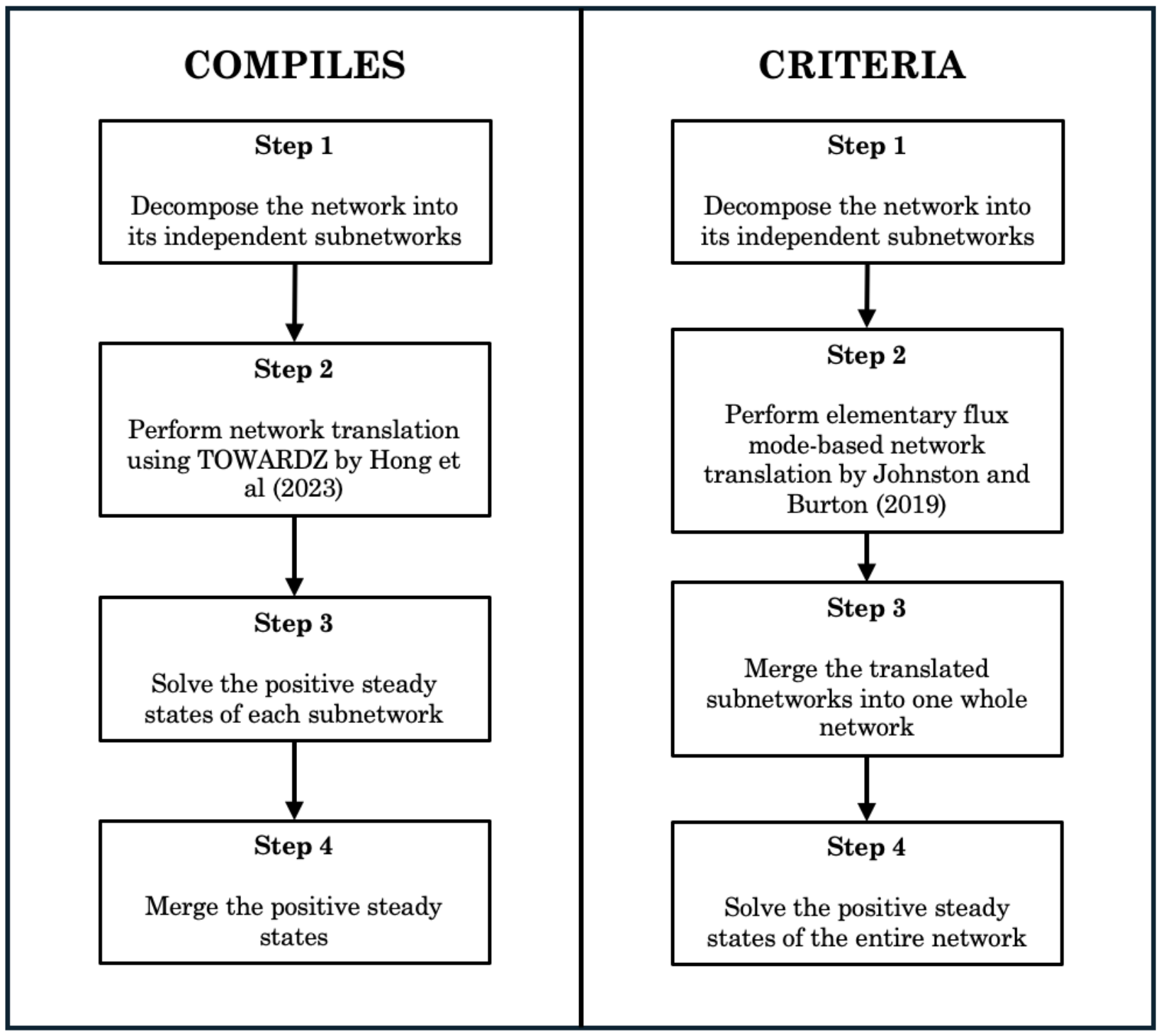

Fig 4. Side-by-side comparison of the COMPILES and CRITERIA frameworks for deriving analytic equilibria. For both frameworks, the method starts by decomposing the network into its independent subnetworks, thereby simplifying the analysis by focusing on smaller individual pieces rather than looking the network as a whole. The next step is to perform network translation for each subnetwork that is not WR and DZ. For COMPILES, translation is performed using the TOWARDZ approach introduced in [23]. On the other hand, CRITERIA uses an elementary flux mode-based approach [5]. Both frameworks differ in the last two steps. Once all subnetworks satisfy WR and DZ, COMPILES proceeds by determining the positive equilibria of each subnetwork through the parametrization method in [22]. Finally, the equilibria of the original network are obtained by merging the equilibria of the subnetworks. However, for CRITERIA, it first performs merging of the translated subnetworks into a single network by taking the union of the reactions in the subnetworks before proceeding to compute the positive equilibria of the entire network, which constitutes the last step of the enhanced procedure.

Comment 10

We revise as follows:

(Page 2, line 64)

deriving

(Page 11, line 365)

worth noting

(Page 19, line 541)

producing

(Page 20, line 557)

equilibrium

(Page 15, line 447)

autophosphorylation

(Page 20)

CRISPRi

Comment 11

To remove the confusion, we rename Theorem 1 in the Supplementary Information as Theorem S1. Moreover, we rename equation numbers in the *Construction of the reaction-to-reaction graph* section from (S1), (S2), ..., (S7) to (C1), (C2), ..., (C7), respectively, to avoid ambiguity from Theorem S1.

We revise as follows:

(*Preservation of weak reversibility and zero deficiency under network merging of independent subnetworks* section, Page 1)

Theorem S1 *Let $\mathcal{N}$ be a chemical reaction network (CRN) with α independent subnetworks $\mathcal{N}_1, \mathcal{N}_2, \ldots, \mathcal{N}_\alpha$. Suppose that for each $i = 1, 2, \ldots, \alpha$, there exists a weakly reversible and deficiency zero translation $\widetilde{\mathcal{N}}_i$ for $\mathcal{N}_i$. Then the network $\mathcal{N}' = \bigcup_{i=1}^{\alpha} \widetilde{\mathcal{N}}_i$ admits a generalized CRN such that its stoichiometric CRN $\mathcal{N}'_S$ is weakly reversible and deficiency zero, and its kinetic-order CRN $\mathcal{N}'_K$ is weakly reversible.*

(*Preservation of weak reversibility and zero deficiency under network merging of independent subnetworks* section, Page 2)

Consequently, in Theorem S1, it is not necessary to impose the condition that the kinetic-order CRN is DZ.

(*Construction of the reaction-to-reaction graph* section, Pages 5-7)

Equation numbers are renamed from (S1), (S2), ..., (S7) to (C1), (C2), ..., (C7), respectively.

Reviewer 2

Summary

This paper addresses the problem of computing steady state parametrizations for mass-action systems. In previous work, the authors utilized the network-based process of network translation to construct a MATLAB program called COMPILES. This work extends that program in a new direction by utilizing an elementary-flux-mode-based algorithm for determining network translations. The authors also swap the order of steady state determination and network recombination, leaving the algebraic step of steady state determination for the final step. The authors present a MATLAB program called CRITERIA which includes modules for both the translation and steady state parametrization portions of this workflow. The authors present documentation and a number of interesting worked out examples, including the EnvZ-OmpR signaling pathway and CRISPRi toggle switch.

The technical details utilized are mathematically sound and the program works incredibly well on all of the examples I checked. The program utilizes two recent theoretical advances which have not previously been implemented computationally in a widespread manner: Johnston and Burton's (2019) elementary flux mode approach to network translation, and Johnston, Muller, and Pantea's (2019) rational parametrization approach to weakly reversible networks. Implementing these theoretical formulas into a single computational package is highly non-trivial as it involves binary programming, convex polyhedral geometry, generalized inverses, and algebraic manipulation, among other steps. The computational test cases and worked-out examples presented in the paper are appropriate.

I expect that, once widely known, this program will be of profound benefit to the chemical reaction network and systems biology communities.

We appreciate the kind comments of the reviewer.

Comment 1

Line 11: "Analytically deriving equilibria, sometimes referred to as steady states, which often..." Remove "sometimes referred to as steady states"

We revise as follows:

(Page 1, lines 11-13)

Analytically deriving equilibria, which often govern the long-term behavior of biochemical reaction networks, is essential for understanding cellular decision-making and robustness, yet remains computationally challenging for large and complex systems.

Comment 2

Lines 55-56: "... since they characterize the long-term behavior of these systems." They "sometimes characterize" or "often characterize" the long-term behavior (limit cycles, chaos, and unbounded solutions are possible too)

The revision is given as follows:

(Page 2, lines 52-55)

The study of equilibria is fundamental to understanding biochemical systems, which are typically modeled using ordinary differential equations based on mass-action kinetics, since they often characterize the long-term behavior of these systems.

Comment 3

Equation between lines 101-102: Perhaps a better example could be used here? I am not sure what the problem is here since XpY in the second equation could be substituted into the first equation and then you just have XD=0 or some equation for sigma1. If you had XD in the denominator or something like that, you would end up with a quadratic and the point might be more clear. That said, it seems like you are drawing from a real example, but I might suggest picking a more uncomfortable example than the one presented.

We thank the reviewer for this observation. We agree that the original EnvZ–OmpR example does not exhibit a particularly complicated algebraic dependency. Our intent was not to suggest that the resulting equations are difficult to solve, but rather to illustrate that COMPILES may return recursive equilibrium expressions involving other species variables instead of expressions solely in terms of the free parameters. We have replaced the EnvZ-OmpR example with a better one, and revised the manuscript to explain more clearly and in detail this issue.

The revisions are as follows:

(Page 3, lines 120-132)

For instance, COMPILES obtains an equilibrium parametrization of the following species in the MAPK model [5] with their corresponding functional forms as follows

$$X_{pp} = f(X_{pp}M, X_p^*M, X_p, \sigma_1, \sigma_2), \quad X_{pp}M = g(X_{pp}, X_p^*M, X_p), \quad X_p = h(X_{pp}, \sigma_2)$$

where only X_p^*M, σ_1, and σ_2 are classified by COMPILES as free parameters. It can be observed that both the species X_{pp} and $X_{pp}M$ appear in each other's equilibrium expressions. Although COMPILES identifies equilibrium relations among the species, its output is not immediately an explicit equilibrium parametrization because some species remain recursively defined in terms of other species. As a result, the equilibrium concentrations cannot be computed directly from the chosen free parameters without further

algebraic manipulation. Merging equilibrium expressions requires extensive symbolic manipulation, which may cause MATLAB to fail in obtaining a closed-form solution free of interdependencies. Although not intrinsic to COMPILES, it nonetheless limits the method's applicability to more complex biochemical networks.

(Page 10, lines 330-341)

To illustrate this issue more clearly, we consider again the MAPK example in the Introduction. The parametrization obtained by COMPILES for the species X_{pp} and $X_{pp}M$ is given by

$$\boldsymbol{X_{pp}} = \frac{X_p \boldsymbol{X_{pp}M} k_8 k_{11} k_{12} \sigma_1 + X_p \boldsymbol{X_{pp}M} k_9 k_{11} k_{12} \sigma_1}{X_p^* M k_7 k_{13} k_{16} \sigma_2 + X_p^* M k_7 k_{14} k_{16} \sigma_2}$$

$$\boldsymbol{X_{pp}M} = \frac{\boldsymbol{X_{pp}} X_p^* M k_7 (k_{13} + k_{14})}{X_p k_{12} (k_8 + k_9)}.$$

There is an interdependency issue in this example because the two parametrization equations depend on each other in a circular way, so that none of the involved species can be expressed solely in terms of the free parameters and rate constants without further algebraic manipulation. If we substitute the equation for $X_{pp}M$ to that of X_{pp}, we arrive at the equation

$$k_{16}\sigma_2 = k_{11}\sigma_1$$

which is a hidden algebraic condition on the parameters. However, after observing this relation, substituting the parametrization into the ODE system does not make all time derivatives vanish indicating that the output COMPILES obtained is incorrect. This is what we aim to address as far as the interdependency issue is concerned.

Comments 4-10

4. Line 61: "Recent research has focused on network-based approaches in DERIVERING analytic..." DELIVERING

5. Lines 97-98: "Second, there is a merging issue in COMPILES resulting TO interdependencies in the derived equilibrium parametrization." resulting in

6. Lines 163-164: "Several variations of the algorithm EXISTS." exist

7. Lines 241-242: "... while assigning to each of the source NODE of the edges..." nodes

8. Line 284: "It is WOTH noting here that all benchmark models are..." WORTH noting

9. Figure 1: "... the complex in parentheses is the kinetic complex associated IN that node." with

10. Line 324: "Now, it can be observed that..." "Now" is too colloquial for a journal paper. I would suggest simply "It can be observed that..."

Comment 4

The revision is as follows: (Page 2, line 64)
deriving

Comment 5

The revision is as follows: (Page 3, lines 117-118)
resulting in

Comment 6

The revision is as follows: (Page 5, line 191)
exist

Comment 7

The revision is as follows: (Page 9, line 284)
nodes

Comment 8

The revision is as follows: (Page 11, line 365)
worth

Comment 9

The revision is as follows: (Figure 1)
with

Comment 10

The revision is as follows: (Page 15, lines 415-416)
It can be observed that COMPILES performed slightly better than CRITERIA in six models.

Comment 11

Equation between lines 369 and 370: It is interesting and nonstandard that you allow conservation laws with negative coefficients. This also seems to be a feature of the computational package. This is particular interesting because there is an obvious second conservation law with positive coefficient for this example (Y+Yp+XpY+XDYp+XTYp=constant). Is it guaranteed that this will not lead to problems for positivity of the final parametriza-

tion? Is this just for show? At the very least, the possibility of conservation laws with negative coefficients should be noted in the paper.

We thank the reviewer for this observation. The second conservation law of the EnvZ-OmpR example has negative coefficients because of the way conservation equations are computed by CRITERIA. Conservation laws can be computed by determining the left null space of the stoichiometric matrix of the CRN. That is, a conservation equation is represented by a vector c such that

$$cN = \mathbf{0}, \tag{1}$$

where N is the stoichiometric matrix. Equivalently, finding the vector c requires solving the null space of N^T. In our EnvZ-OmpR example, the stoichiometric matrix N is given by

$$N = \begin{array}{c} \\ X \\ XD \\ XDY_p \\ XT \\ XTY_p \\ X_p \\ X_pY \\ Y \\ Y_p \end{array} \begin{array}{c} \begin{array}{cccccccccccccc} R_1 & R_2 & R_3 & R_4 & R_5 & R_6 & R_7 & R_8 & R_9 & R_{10} & R_{11} & R_{12} & R_{13} & R_{14} \end{array} \\ \begin{bmatrix} 1 & -1 & -1 & 1 & 0 & 0 & 0 & 1 & 0 & 0 & 0 & 0 & 0 & 0 \\ -1 & 1 & 0 & 0 & 0 & 0 & 0 & 0 & -1 & 1 & 1 & 0 & 0 & 0 \\ 0 & 0 & 0 & 0 & 0 & 0 & 0 & 0 & 1 & -1 & -1 & 0 & 0 & 0 \\ 0 & 0 & 1 & -1 & -1 & 0 & 0 & 0 & 0 & 0 & 0 & -1 & 1 & 1 \\ 0 & 0 & 0 & 0 & 0 & 0 & 0 & 0 & 0 & 0 & 0 & 1 & -1 & -1 \\ 0 & 0 & 0 & 0 & 1 & -1 & 1 & 0 & 0 & 0 & 0 & 0 & 0 & 0 \\ 0 & 0 & 0 & 0 & 0 & 1 & -1 & -1 & 0 & 0 & 0 & 0 & 0 & 0 \\ 0 & 0 & 0 & 0 & 0 & -1 & 1 & 0 & 0 & 0 & 1 & 0 & 0 & 1 \\ 0 & 0 & 0 & 0 & 0 & 0 & 0 & 1 & -1 & 1 & 0 & -1 & 1 & 0 \end{bmatrix} \end{array}.$$

Performing Gaussian elimination on N^T to obtain its RREF gives us

$$\begin{array}{c} \begin{array}{ccccccccc} X & XD & XDY_p & XT & XTY_p & X_p & X_pY & Y & Y_p \end{array} \\ \begin{bmatrix} 1 & 0 & 0 & 0 & 0 & 0 & -1 & 0 & 1 \\ 0 & 1 & 0 & 0 & 0 & 0 & -1 & 0 & 1 \\ 0 & 0 & 1 & 0 & 0 & 0 & -1 & 0 & 0 \\ 0 & 0 & 0 & 1 & 0 & 0 & -1 & 0 & 1 \\ 0 & 0 & 0 & 0 & 1 & 0 & -1 & 0 & 0 \\ 0 & 0 & 0 & 0 & 0 & 1 & -1 & 0 & 1 \\ 0 & 0 & 0 & 0 & 0 & 0 & 0 & 1 & -1 \\ 0 & 0 & 0 & 0 & 0 & 0 & 0 & 0 & 0 \\ 0 & 0 & 0 & 0 & 0 & 0 & 0 & 0 & 0 \\ 0 & 0 & 0 & 0 & 0 & 0 & 0 & 0 & 0 \\ 0 & 0 & 0 & 0 & 0 & 0 & 0 & 0 & 0 \\ 0 & 0 & 0 & 0 & 0 & 0 & 0 & 0 & 0 \\ 0 & 0 & 0 & 0 & 0 & 0 & 0 & 0 & 0 \\ 0 & 0 & 0 & 0 & 0 & 0 & 0 & 0 & 0 \end{bmatrix} \end{array}$$

which corresponds to the vector c as in Equation 1

$$c = \begin{bmatrix} X_pY - Y_p \\ X_pY - Y_p \\ X_pY \\ X_pY - Y_p \\ X_pY \\ X_pY - Y_p \\ X_pY \\ Y_p \\ Y_p \end{bmatrix} = X_pY \begin{bmatrix} 1 \\ 1 \\ 1 \\ 1 \\ 1 \\ 1 \\ 1 \\ 0 \\ 0 \end{bmatrix} + Y_p \begin{bmatrix} -1 \\ -1 \\ 0 \\ -1 \\ 0 \\ -1 \\ 0 \\ 1 \\ 1 \end{bmatrix},$$

where X_pY and Y_p are the free variables in the RREF of N^T. This yields two basis vectors in the left null space of N. We can use any two different basis vectors by setting particular values for the free variables to get a set of valid conservation laws of the system as they span the exact same left null space of N. In particular, setting $X_pY = 1$ and $Y_p = 0$ gives us the conservation equation

$$X + XD + XDY_p + XT + XTY_p + X_p + X_pY = T_1, \tag{2}$$

where T_1 is a constant determined by the initial concentrations of the species involved in the equation. Furthermore, if we set $X_pY = 0$ and $Y_p = 1$, we get

$$-X - XD - XT - X_p + Y + Y_p = T_2, \tag{3}$$

where T_2 is likewise a constant determined by the initial concentrations of the species involved in the equation. These are the conservation equations obtained by CRITERIA. This demonstrates the possibility of having negative coefficients in the conservation laws. Notice that we can also set $X_pY = 1$ and $Y_p = 1$ to get the equation as specified by the reviewer

$$XDY_p + XTY_p + X_pY + Y + Y_p = T_3, \tag{4}$$

which is also a valid conservation law together with either of the conservation equations above. This is the same as adding the corresponding left and right sides of Equations 2 and 3 to obtain Equation 4 with $T_3 = T_1 + T_2$, which is also a constant.

Regarding positivity of the final equilibrium parametrization, we wish to clarify that the conservation laws are not used in its derivation. In CRITERIA, conservation laws are computed independently (via the procedure explained above) from the parametrization procedure. In other words, the derivation of the equilibrium parametrization and the computation of conservation laws are independent procedures. Therefore, the choice of conservation equations has no effect on the resulting equilibrium parametrization or its positivity properties.

We agree with the reviewer that this should be clarified in the manuscript. So, we have added some remarks to explain this more clearly. We have also included an additional

section in the Supplementary Information describing the computation of conservation laws to demonstrate why conservation laws with negative coefficients may arise in the output.

The revisions are as follows:

(Page 17, lines 469-483)

It is important to note that the conservation laws reported by CRITERIA are obtained by computing a basis for the left null space of the stoichiometric matrix of the reaction network [24,29,30]. Consequently, the reported conservation laws are not guaranteed to have positive coefficients as can be observed by the second conservation law of the EnvZ–OmpR model. This, however, does not affect the final equilibrium parametrization since conservation laws are not used in its derivation. In other words, the derivation of the equilibrium parametrization and the computation of conservation laws are independent procedures.

Therefore, the choice of a basis for the left null space of the stoichiometric matrix has no effect on the equilibrium parametrization or its positivity properties. In particular, replacing a reported conservation law with another valid conservation law with negative coefficients would not alter any of the steady state expressions generated by CRITERIA. Such valid conservation laws can always be obtained as linear combinations of the reported basis vectors. A detailed discussion on the computation of conservation laws is provided in the Supplementary Information.

(Supplementary Information, pages 9-11)

Detailed computation of conservation laws in the EnvZ-OmpR model

Conservation laws of a CRN can be computed by determining the left null space of its stoichiometric matrix [5-7]. That is, a conservation equation is represented by a vector c such that

$$cN = \mathbf{0}, \tag{5}$$

where N is the stoichiometric matrix. Equivalently, finding the vector c requires solving the null space of N^T. In our EnvZ-OmpR example, the stoichiometric matrix N is given

by

$$N = \begin{array}{c} \\ X \\ XD \\ XDY_p \\ XT \\ XTY_p \\ X_p \\ X_pY \\ Y \\ Y_p \end{array} \begin{array}{c} \begin{array}{cccccccccccccc} R_1 & R_2 & R_3 & R_4 & R_5 & R_6 & R_7 & R_8 & R_9 & R_{10} & R_{11} & R_{12} & R_{13} & R_{14} \end{array} \\ \begin{bmatrix} 1 & -1 & -1 & 1 & 0 & 0 & 0 & 1 & 0 & 0 & 0 & 0 & 0 & 0 \\ -1 & 1 & 0 & 0 & 0 & 0 & 0 & 0 & -1 & 1 & 1 & 0 & 0 & 0 \\ 0 & 0 & 0 & 0 & 0 & 0 & 0 & 0 & 1 & -1 & -1 & 0 & 0 & 0 \\ 0 & 0 & 1 & -1 & -1 & 0 & 0 & 0 & 0 & 0 & 0 & -1 & 1 & 1 \\ 0 & 0 & 0 & 0 & 0 & 0 & 0 & 0 & 0 & 0 & 0 & 1 & -1 & -1 \\ 0 & 0 & 0 & 0 & 1 & -1 & 1 & 0 & 0 & 0 & 0 & 0 & 0 & 0 \\ 0 & 0 & 0 & 0 & 0 & 1 & -1 & -1 & 0 & 0 & 0 & 0 & 0 & 0 \\ 0 & 0 & 0 & 0 & 0 & -1 & 1 & 0 & 0 & 0 & 1 & 0 & 0 & 1 \\ 0 & 0 & 0 & 0 & 0 & 0 & 0 & 1 & -1 & 1 & 0 & -1 & 1 & 0 \end{bmatrix} \end{array}.$$

Performing Gaussian elimination on N^T to obtain its RREF gives us

$$\begin{array}{c} \begin{array}{ccccccccc} X & XD & XDY_p & XT & XTY_p & X_p & X_pY & Y & Y_p \end{array} \\ \begin{bmatrix} 1 & 0 & 0 & 0 & 0 & 0 & -1 & 0 & 1 \\ 0 & 1 & 0 & 0 & 0 & 0 & -1 & 0 & 1 \\ 0 & 0 & 1 & 0 & 0 & 0 & -1 & 0 & 0 \\ 0 & 0 & 0 & 1 & 0 & 0 & -1 & 0 & 1 \\ 0 & 0 & 0 & 0 & 1 & 0 & -1 & 0 & 0 \\ 0 & 0 & 0 & 0 & 0 & 1 & -1 & 0 & 1 \\ 0 & 0 & 0 & 0 & 0 & 0 & 0 & 1 & -1 \\ 0 & 0 & 0 & 0 & 0 & 0 & 0 & 0 & 0 \\ 0 & 0 & 0 & 0 & 0 & 0 & 0 & 0 & 0 \\ 0 & 0 & 0 & 0 & 0 & 0 & 0 & 0 & 0 \\ 0 & 0 & 0 & 0 & 0 & 0 & 0 & 0 & 0 \\ 0 & 0 & 0 & 0 & 0 & 0 & 0 & 0 & 0 \\ 0 & 0 & 0 & 0 & 0 & 0 & 0 & 0 & 0 \\ 0 & 0 & 0 & 0 & 0 & 0 & 0 & 0 & 0 \end{bmatrix} \end{array}$$

which corresponds to the vector c as in Eq 5

$$c = \begin{bmatrix} X_pY - Y_p \\ X_pY - Y_p \\ X_pY \\ X_pY - Y_p \\ X_pY \\ X_pY - Y_p \\ X_pY \\ Y_p \\ Y_p \end{bmatrix} = X_pY \begin{bmatrix} 1 \\ 1 \\ 1 \\ 1 \\ 1 \\ 1 \\ 1 \\ 0 \\ 0 \end{bmatrix} + Y_p \begin{bmatrix} -1 \\ -1 \\ 0 \\ -1 \\ 0 \\ -1 \\ 0 \\ 1 \\ 1 \end{bmatrix},$$

where X_pY and Y_p are the free variables in the RREF of N^T. This yields two basis vectors in the left null space of N. We can use any two different basis vectors by setting particular

values for the free variables to get a set of valid conservation laws of the system as they span the exact same left null space of N. In particular, setting $X_pY = 1$ and $Y_p = 0$ gives us the conservation equation

$$X + XD + XDY_p + XT + XTY_p + X_p + X_pY = T_1, \tag{6}$$

where T_1 is a constant determined by the initial concentrations of the species involved in the equation. Furthermore, if we set $X_pY = 0$ and $Y_p = 1$, we get

$$-X - XD - XT - X_p + Y + Y_p = T_2, \tag{7}$$

where T_2 is likewise a constant determined by the initial concentrations of the species involved in the equation. These are the conservation equations obtained by CRITERIA. Notice that we can also set $X_pY = 1$ and $Y_p = 1$ to get the equation

$$XDY_p + XTY_p + X_pY + Y + Y_p = T_3, \tag{8}$$

which is also a valid conservation law together with either of the conservation equations above. This is the same as adding the corresponding left and right sides of Eq 6 and Eq 7 to obtain Eq 8 with $T_3 = T_1 + T_2$, which is also a constant.

Comment 12

Example in paragraph bound by lines 382 and 389: It is striking that you are able to show multistationarity as well as absolute concentration robustness for this example. To the best of my knowledge, this is the first time this has been shown for this model. This is very interesting!

We appreciate the kind comment of the reviewer.

Comment 13

General note on elementary flux modes: It should be noted that the elementary flux mode approach will only work if the coefficients of all the reactions in every elementary flux mode can be scaled to one. For example, the method will not work if one of the elementary flux modes has coefficients (1,2,1)—that is, one firing of the first reaction, two of the second, and one of the third are required to obtain stoichiometric balance. Since this limitation inherited from the underlying Johnston and Burton paper will be a limitation here as well, it should be mentioned.

We thank the reviewer for pointing this out. We have added a remark in the main theorem of the translation procedure under the Methods section emphasizing that the method only works for reaction networks whose set of EFMs is unitary. This is also mentioned in the first item of our recommendations in the Conclusion section.

We revise as follows:

(Page 31, lines 935-943)

Remarks

1. *It is important to note that Theorem 3 is only applicable when the set of EFMs of the reaction network is unitary. Consequently, CRITERIA cannot obtain an equilibrium parametrization of a CRN when its set of EFMs is non-unitary. The limitation of the method in [5] is inherited in CRITERIA.*
2. *As shown in Table 3, REWARDZ without network decomposition was unable to generate a valid translation for Model N because its set of EFMs is non-unitary. However, this limitation was overcome by first performing network decomposition, after which REWARDZ successfully obtained a valid translation.*

(Page 23, line 661-667)

The translation method is currently applicable only to reaction networks whose set of EFMs are unitary (see Remark of Theorem 3 in the Methods section). Future work will focus on adapting the translation procedure to account for models whose EFMs are not unitary. A promising avenue is to investigate the effect of network decomposition on the EFMs of a reaction network. In particular, it would be of interest to characterize the conditions under which network decomposition transforms a non-unitary set of EFMs into a unitary one.

Comments 14-17

14. Lines 433-434: "ACR suggests an intrinsic buffering mechanism wherein the EQULIBRIUM concentration of dCas9 remains..." equilibrium
15. Line 454: "... (see Fig 5 and its ACCOMPANIED discussion)." accompanying
16. Figure 4: "... thereby simplifying the analysis by focusing on smaller individual pieces rather than looking the network as a whole." rather than looking AT
17. Line 687: "In this subsection, we formally define the concept of a GCRN, which IS pioneered by..." was

<u>Comment 14</u>

The revision is as follows: (Page 20, line 557)

equilibrium

<u>Comment 15</u>

The revision is as follows: (Page 21, line 577)

accompanying

<u>Comment 16</u>

The revision is as follows: (Figure 4)

at

<u>Comment 17</u>

The revision is as follows: (Page 28, line 819)

was

Comment 18

Note on CRITERIA: The CRITERIA program works very well in the examples I checked. There are two notes I would like to make:

(1) It would be very nice if a user-friendly interface for implementing networks (like CRN-Tool or CoNtRol) could be developed. That said, the program is very well documented and I have no problem digging into the prebuilt MATLAB programs to change networks. (2) For higher kinetic deficiency systems, the program does not seem to identify parameters generated from phantom edges (i.e., the sigmas) as free parameters. For example, when I run ModelL.m, it gives a parametrization including the parameter sigma1 but only says "Free parameters: XpY, Y".

- We thank the reviewer for this comment. We agree that it would be nice to develop a user-friendly interface or an application similar to CRNToolbox or CoNtRol that would implement the framework proposed in this paper, as the current package is fully MATLAB-based. We incorporate this suggestion as a recommendation in the main manuscript. This would be interesting for future work.

- We thank the reviewer for pointing this out. Indeed, the program does not identify the sigmas and taus as free parameters in the final display. To address this, we added some lines in the main function (i.e., `ComputeEquilibria.m`) to correctly display all free parameters in the final parametrization.

  Figure 5 shows the updated output of CRITERIA in the EnvZ-OmpR model. Here, sigma1 is already listed as a free parameter in the final parametrization. Furthermore, we emphasized in the updated output the number of free parameters that can be solved using the additional conditions stated in Case 2 of Theorem 2 of the main manuscript. In the EnvZ-OmpR model, the kinetic deficiency is one. As shown in Figure 5, the updated output of CRITERIA indicates that one free parameter can be solved. Looking at the *Additional conditions to the parametrization* line in the

output of CRITERIA, we have

$$\begin{aligned} 1 &= \frac{k_1 k_3 k_{12}}{k_2 \sigma_1 (k_4 + k_5)} \\ \implies \sigma_1 &= \frac{k_1 k_3 k_{12}}{k_2 (k_4 + k_5)}. \end{aligned}$$

For models with positive kinetic deficiency, the additional conditions are displayed separately from the final parametrization.

```
Solving positive steady state parametrization of the network...

The solution is:

X = (XpY*k8*(k4 + k5))/(k3*k5)
XD = (XpY*k2*k8*(k4 + k5))/(k1*k3*k5)
XDYp = (XpY*k8*k9*(k13 + k14))/(k9*k11*k13 + k9*k11*k14 + k10*k14*sigma1 + k11*k14*sigma1)
XT = (XpY*k8)/k5
XTYp = (XpY*k8*sigma1*(k10 + k11))/(k9*k11*k13 + k9*k11*k14 + k10*k14*sigma1 + k11*k14*sigma1)
Xp = (XpY*(k7 + k8))/(Y*k6)
Yp = (k5*sigma1*(k10 + k11)*(k13 + k14))/(k12*(k9*k11*k13 + k9*k11*k14 + k10*k14*sigma1 + k11*k14*sigma1))
Free parameters: XpY, Y, sigma1

Kinetic deficiency is positive.
Number of free parameters that can be solved: 1
Refer to the additional conditions displayed above.
```

Figure 5: Updated output of CRITERIA in the EnvZ-OmpR model.

We revise as follows:

(Page 24, lines 683-689)

Future work may also focus on developing a user-friendly interface or application that implements the enhanced computational framework proposed in this study, and hence extends its accessibility beyond the current MATLAB environment.In addition, this could be expanded to incorporate other analyses and computational methods in CRN theory that are not yet available in existing applications. This would provide a more comprehensive platform that researchers in the field of applied mathematics, biochemistry, and systems biology can readily use.

(Supplementary Information, page 13)

Based on the kinetic deficiency, the function outputs how many free parameters can be solved using the additional conditions.

(Supplementary Information, page 14)

```
Solving positive steady state parametrization of the network...

The solution is:
```

```
X = (XpY*k8*(k4 + k5))/(k3*k5)
XD = (XpY*k2*k8*(k4 + k5))/(k1*k3*k5)
XDYp = (XpY*k8*k9*(k13 + k14))/(k9*k11*k13 + k9*k11*k14 + k10*k14*sigma1 +
k11*k14*sigma1)
XT = (XpY*k8)/k5
XTYp = (XpY*k8*sigma1*(k10 + k11))/(k9*k11*k13 + k9*k11*k14 + k10*k14*sigma1
+ k11*k14*sigma1)
Xp = (XpY*(k7 + k8))/(Y*k6)
Yp = (k5*sigma1*(k10 + k11)*(k13 + k14))/(k12*(k9*k11*k13 + k9*k11*k14 +
k10*k14*sigma1 + k11*k14*sigma1))
Free parameters:  XpY, Y, sigma1

Kinetic deficiency is positive.
Number of free parameters that can be solved:  1
Refer to the additional conditions displayed above.

Conservation laws:
dX/dt + dXD/dt + dXDYp/dt + dXT/dt + dXTYp/dt + dXp/dt + dXpY/dt = 0
-dX/dt - dXD/dt - dXT/dt - dXp/dt + dY/dt + dYp/dt = 0
Elapsed time is 2.731821 seconds.
```

Reviewer 3

Summary

This paper introduces introduces CRITERIA, a MATLAB-based computational framework for deriving analytic positive equilibria (parametrized steady states) of biochemical reaction networks modeled under mass-action kinetics, with a properly working MATLAB implementation available on GitHub. This work is presented as an improvement of the authors' prior framework COMPILES (Hernandez et al., PLoS Comput Biol. 2023;19(4):e1011039), which relies on the concept of network translation and the theory of generalized chemical reaction networks. The authors provide the three main improvements:

1. This resolves the issue of prohibitively large computation of COMPILES when generating candidate translated networks, by replacing the exhaustive search with a more efficient approach based on elementary flux modes (EFMs) introduced by Johnston and Burton.
2. This resolves the inter-dependencies of in derived steady-state parametrizations (e.g., XD and XpY equations on page 3), by computing steady states on a single unified translated network rather than solving subnetworks independently.
3. This extends the applicability of COMPILES from the class of CRNs with kinetic deficiency zero to a broader class of CRNs (c.f. Theorem 2 - Case 2 in the manuscript).

This work certainly provides a well justified computational framework for computing steady states of a system of ODEs arising from biochemical reaction networks under the mass-action kinetics assumption. Although there seems to be no mathematically novel results, the framework is useful and practical for researchers in the field of chemical reaction network theory and systems biology.

In summary, this paper is a nice contribution to the field of studying steady states of biochemical reaction networks. However, I have some major comments to be addressed before the paper can be accepted to PLOS Computational Biology, and I also have some minor comments that the authors should consider. Overall, I recommend a major revision.

We are grateful to the reviewer for appreciating the contribution of our work.

Comment 1

The presentation of the main framework, CRITERIA, can be improved. The authors explain the main idea using a running example on page 4 with Steps 1–3. However, they do not quite correspond to Steps 1–4 of Figure 1, which is also intended to illustrate the main framework. Also, Figure 1 uses a different (more complex) example on page 7.

I think they should share the same example and be more consistent with explicit Step numbers.

It would be significantly better if the authors can revise the Results section and Figure 1 to make them more consistent. Also, they should refer to each part of Figure 1 more explicitly in the text. Currently, only Panels (a) and (b) of Figure 1 are referred to few times in the text. In this way, the readers could follow the main framework with the Results section and Figure 1 together, which would make the presentation much clearer and more intuitive.

In the main manuscript, there are two processes that are demonstrated to discuss the changes made in COMPILES to produce CRITERIA. The first process is the elementary flux mode-based network translation process, which uses the histidine kinase system as an example to illustrate the translation steps. The second process is the main framework of the study, which is the derivation of the analytic steady states. This uses a two-protein gene transcription model as an example. The main framework has four main steps as shown in Figure 1. The second step, which is the EFM-based network translation, has three substeps which we now label as Step T1, T2, and T3 to avoid confusion. This is the first process demonstrated in the main text.

The rationale behind using two different examples is that the two-protein gene transcription model better illustrates the main framework because it is a simple network which has several independent subnetworks. It illustrates the merging of the different subnetworks as a one whole, unified network before solving the positive steady states. On the other hand, the histidine kinase system better illustrates the elementary flux mode-based network translation step because it is a nontrivial example that simply demonstrates each step of the translation procedure. If we were to use the gene transcription network to illustrate the translation process, we would not be able to clearly explain and demonstrate every step of the procedure since each subnetwork only contains two reactions. On the other hand, if we were to use the histidine kinase system to present the main framework, we would be unable to illustrate the decomposition and merging steps because the network does not admit a nontrivial independent decomposition. Thus, the selected examples were chosen to highlight different aspects of the proposed framework.

Lastly, we have expanded the accompanying discussion of Figure 1 to present clearly and in detail all steps of the main framework using the chosen example. We agree that, in the original submission, only panels (a) and (b) were discussed in detail. Accordingly, we have added explanations for panels (c) and (d) to complete the presentation of the framework and to guide the reader through the remaining steps.

The revisions are as follows:

(Page 4, line 178)

Step T1.

(Page 6, line 199)

Step T2.

(Page 6, line 216)

Step T3.

(Page 7, lines 245-250)

We use a different example to better illustrate the framework. The process involves decomposing the network into its independent subnetworks and subsequently merging translated versions of the subnetworks. The histidine kinase example does not admit a nontrivial independent decomposition, so it does not demonstrate these decomposition and merging steps.

(Page 9, line 265)

three-step

(Pages 10, lines 302-316)

In the two-protein gene transcription example, Fig 1c illustrates how the subnetworks are merged. This is simply done by taking the union of the reaction sets of the translated subnetworks. Since the subnetworks do not share any common complexes, the merging process preserves the interconnections within each subnetwork, effectively treating them as components of a single larger network. It can be easily verified that the resulting merged network is both WR and DZ. In particular, both the stoichiometric CRN and kinetic-order CRN of the merged network are WR and DZ. This is also guaranteed by Theorem S1 in the Supplementary Information.

Finally, we compute the equilibrium parametrization using the full network rather than treating each subnetwork individually. We refer the readers to [2,22] for a detailed discussion on equilibrium parametrization. Fig 1d shows the final equilibrium parametrization of the two-protein gene transcription model. It can be observed that P_1 and P_2 are free parameters in the parametrization. If we set particular values for the rate constants and free parameters P_1 and P_2, we get the steady state concentration values of the species G_1, G_2, D, C_1, and C_2.

(Figure 1)

The translation complexes for $\mathcal{N}_1$ and $\mathcal{N}_2$ are solved via Steps T1, T2, and T3 discussed in the previous subsection.

Comment 2

Regarding the 3rd improvement of CRITERIA about the applicability to CRNs with positive kinetic deficiency, the authors provide many working examples in Table 2 and relevant propoistion, Theorem 2 - Case 2. However, it is not clear why the preivous framework COMPILES cannot be applied to these examples, while CRITERIA can be

applied. The authors should provide more detailed discussion on this point, and if possible, provide some examples of CRNs with positive kinetic deficiency that are not covered by COMPILES but are covered by CRITERIA. This would help the readers to better understand the significance of this improvement.
Note that the 1st and 2nd improvements (and even the sources of these improvements) are well justified and clear to me.

We thank the reviewer for highlighting the need to clarify the distinction between COMPILES and CRITERIA regarding their applicability to networks with positive kinetic deficiency. The reviewer is correct in saying that the previous COMPILES framework lacks the capability to handle these cases. Its core algorithm does not consider the theoretical foundations for models with positive kinetic deficiency according to Case 2 of Theorem 2. In other words, COMPILES do not process and output these additional algebraic conditions necessary for the final parametrization of networks with positive kinetic deficiency. For example, the MAPK model (Model Q) considered in the study has kinetic deficiency equal to one. When COMPILES is used to derive its analytic steady states, it is unable to complete the parametrization because it does not implement the procedure for deriving the additional algebraic conditions required for reaction networks with positive kinetic deficiency. Figure 6 shows the output of COMPILES for the MAPK model. As can be observed, the required additional condition cannot be seen in the output. For comparison, Figure 7 presents the corresponding output of CRITERIA. Not only does CRITERIA display the required additional condition, it also reports the kinetic deficiency of the model and indicates the number of free parameters that can be solved using these additional conditions. This bridges the gap present in COMPILES.

Moreover, the equilibrium parametrization of COMPILES does not satisfy the governing ODE system of the MAPK model. This may be attributed to the fact that COMPILES does not account for the constraint required by Case 2 of Theorem 2. As shown in Figure 8, substituting the parametrization obtained by COMPILES into the ODE system does not make all time derivatives evaluate to zero indicating that the steady state parametrization generated by COMPILES is not valid.

In this work, we considered ten models with positive kinetic deficiency. Only three of these models (namely, the Insulin Signaling Pathway, the EnvZ-OmpR model, and the MAPK model) were examined by the original Hernandez et al. (2023) paper. Under the original maximum runtime of 10 seconds, COMPILES was able to derive a steady state parametrization only for the Insulin Signaling Pathway model.

For a more comprehensive comparison, we increased the maximum runtime for COMPILES to 3600 seconds and evaluated it on the same three models mentioned above together with seven additional models (Models M, N, O, R, S, Y, and Z). Using this benchmark, CRITERIA successfully derived valid steady state parametrizations for all ten models. The runtime was less than 7 seconds for every model except Model Z, for which CRITERIA required approximately 13 seconds.

In contrast, COMPILES failed to derive a parametrization for eight of the ten models.

```
Command Window
The network has 2 subnetworks.

- Subnetwork 1 -

R1: X+K->XK
R2: XK->X+K
R3: XK->Xp+K
R12: Xp+M->XpsM
R13: XpsM->Xp+M
R14: XpsM->XM
R15: XM->X+M
R16: X+M->XM

Solving Subnetwork 1...

  609.7415

K = (k16*tau2*(k13 + k14))/(k12*sigma1*tau1)
M = (tau2*(k13 + k14))/(k12*tau1)
X = (k12*k14*sigma1*tau1*(k2 + k3))/(k1*k3*k16*(k13 + k14))
XK = (k14*tau2)/k3
XM = (k14*tau2*(k1*k3 + k2*sigma1 + k3*sigma1))/(k1*k3*k15)
Xp = tau1
XpsM = tau2

- Subnetwork 2 -

R4: Xp+K->XpK
R5: XpK->Xp+K
R6: XpK->Xpp+K
R7: Xpp+M->XppM
R8: XppM->Xpp+M
R9: XppM->XpM
R10: XpM->Xp+M
R11: Xp+M->XpM

Solving Subnetwork 2...

  613.7917

K = (k11*tau4*(k8 + k9))/(k7*sigma2*tau3)
M = (tau4*(k8 + k9))/(k7*tau3)
Xp = (k7*k9*sigma2*tau3*(k5 + k6))/(k4*k6*k11*(k8 + k9))
XpK = (k9*tau4)/k6
XpM = (k9*tau4*(k4*k6 + k5*sigma2 + k6*sigma2))/(k4*k6*k10)
Xpp = tau3
XppM = tau4

Solving positive steady state parametrization of the entire network...

Could not write parametrization in terms of free parameters.
At least Xp and Xpp are dependent on each other.

The solution is:

K = (XpsM*k13*k16 + XpsM*k14*k16)/(Xp*k12*sigma1)
M = (XpsM*(k13 + k14))/(Xp*k12)
X = (Xp*k12*k14*sigma1*(k2 + k3))/(k1*k3*k16*(k13 + k14))
XK = (XpsM*k14)/k3
XM = (XpsM*k14*(k1*k3 + k2*sigma1 + k3*sigma1))/(k1*k3*k15)
Xp = (Xpp*k7*k9*sigma2*(k5 + k6))/(k4*k6*k11*(k8 + k9))
XpK = (XppM*k9)/k6
XpM = (XppM*k9*(k4*k6 + k5*sigma2 + k6*sigma2))/(k4*k6*k10)
Xpp = (Xp*XppM*k8*k11*k12*sigma1 + Xp*XppM*k9*k11*k12*sigma1)/(XpsM*k7*k13*k16*sigma2 + XpsM*k7*k14*k16*sigma2)
XppM = (Xpp*XpsM*k7*(k13 + k14))/(Xp*k12*(k8 + k9))
Free parameter: XpsM

Conservation laws:
dK/dt + dXK/dt + dXpK/dt = 0
-dK/dt - dM/dt + dX/dt + dXp/dt + dXpp/dt = 0
dM/dt + dXM/dt + dXpM/dt + dXppM/dt + dXpsM/dt = 0
```

Figure 6: Output of COMPILES for the MAPK model.

Of the remaining two models, COMPILES produced a correct parametrization only for the Insulin Signaling Pathway model. Although it also generated a parametrization for the MAPK model, that parametrization is incorrect as discussed above.

To make the significance of the improvements of CRITERIA clearer to the reader, we have added another section in the main manuscript to explain in detail what we did to address the issues and limitations of COMPILES.

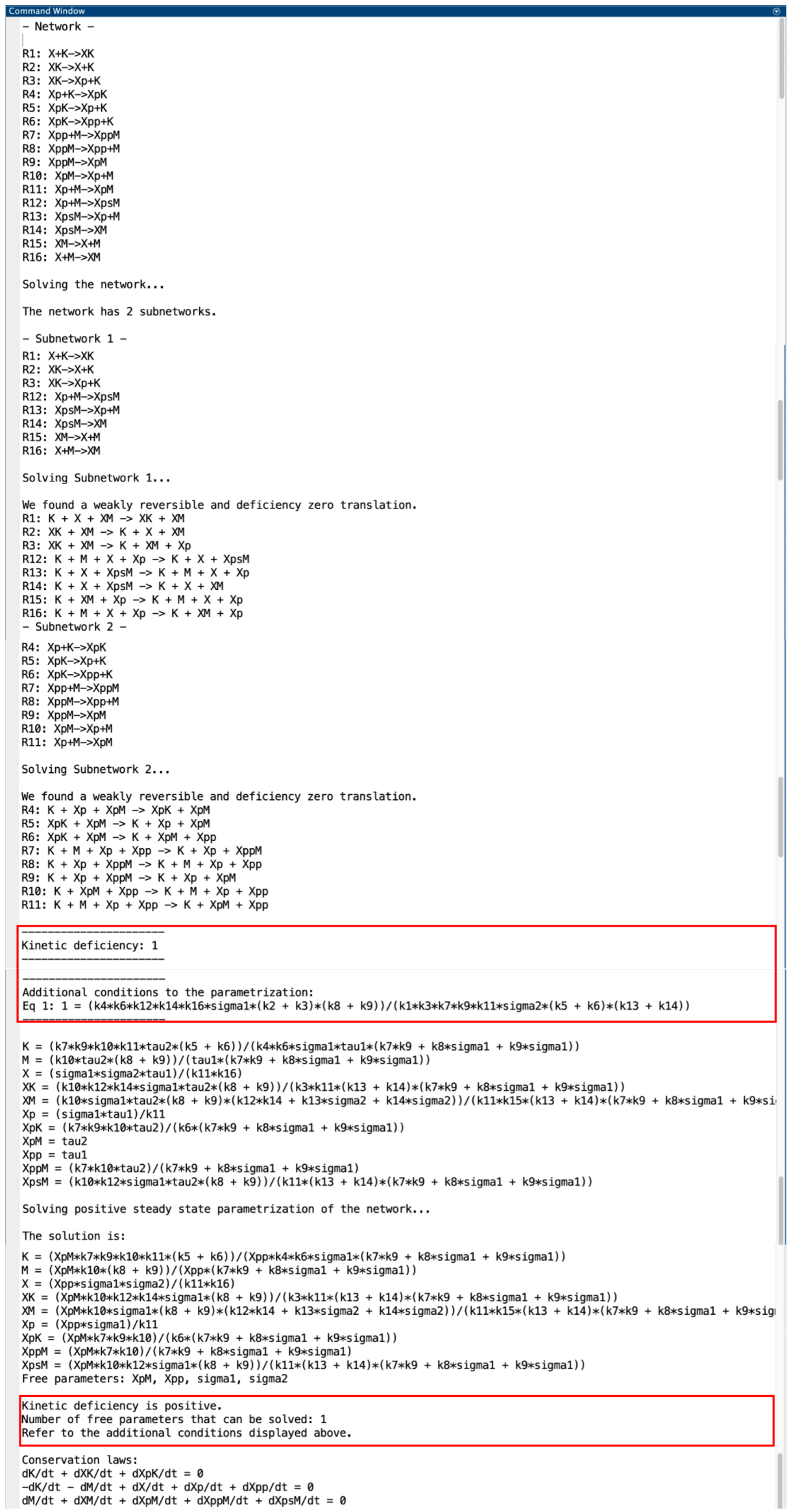

```
Command Window
- Network -

R1: X+K->XK
R2: XK->X+K
R3: XK->Xp+K
R4: Xp+K->XpK
R5: XpK->Xp+K
R6: XpK->Xpp+K
R7: Xpp+M->XppM
R8: XppM->Xpp+M
R9: XppM->XpM
R10: XpM->Xp+M
R11: Xp+M->XpM
R12: Xp+M->XpsM
R13: XpsM->Xp+M
R14: XpsM->XM
R15: XM->X+M
R16: X+M->XM

Solving the network...

The network has 2 subnetworks.

- Subnetwork 1 -
R1: X+K->XK
R2: XK->X+K
R3: XK->Xp+K
R12: Xp+M->XpsM
R13: XpsM->Xp+M
R14: XpsM->XM
R15: XM->X+M
R16: X+M->XM

Solving Subnetwork 1...

We found a weakly reversible and deficiency zero translation.
R1: K + X + XM -> XK + XM
R2: XK + XM -> K + X + XM
R3: XK + XM -> K + XM + Xp
R12: K + M + X + Xp -> K + X + XpsM
R13: K + X + XpsM -> K + M + X + Xp
R14: K + X + XpsM -> K + X + XM
R15: K + XM + Xp -> K + M + X + Xp
R16: K + M + X + Xp -> K + XM + Xp
- Subnetwork 2 -
R4: Xp+K->XpK
R5: XpK->Xp+K
R6: XpK->Xpp+K
R7: Xpp+M->XppM
R8: XppM->Xpp+M
R9: XppM->XpM
R10: XpM->Xp+M
R11: Xp+M->XpM

Solving Subnetwork 2...

We found a weakly reversible and deficiency zero translation.
R4: K + Xp + XpM -> XpK + XpM
R5: XpK + XpM -> K + Xp + XpM
R6: XpK + XpM -> K + XpM + Xpp
R7: K + M + Xp + Xpp -> K + Xp + XppM
R8: K + Xp + XppM -> K + M + Xp + Xpp
R9: K + Xp + XppM -> K + Xp + XpM
R10: K + XpM + Xpp -> K + M + Xp + Xpp
R11: K + M + Xp + Xpp -> K + XpM + Xpp

----------------------
Kinetic deficiency: 1
----------------------

----------------------
Additional conditions to the parametrization:
Eq 1: 1 = (k4*k6*k12*k14*k16*sigma1*(k2 + k3)*(k8 + k9))/(k1*k3*k7*k9*k11*sigma2*(k5 + k6)*(k13 + k14))
----------------------

K = (k7*k9*k10*k11*tau2*(k5 + k6))/(k4*k6*sigma1*tau1*(k7*k9 + k8*sigma1 + k9*sigma1))
M = (k10*tau2*(k8 + k9))/(tau1*(k7*k9 + k8*sigma1 + k9*sigma1))
X = (sigma1*sigma2*tau1)/(k11*k16)
XK = (k10*k12*k14*sigma1*tau2*(k8 + k9))/(k3*k11*(k13 + k14)*(k7*k9 + k8*sigma1 + k9*sigma1))
XM = (k10*sigma1*tau2*(k8 + k9)*(k12*k14 + k13*sigma2 + k14*sigma2))/(k11*k15*(k13 + k14)*(k7*k9 + k8*sigma1 + k9*si
Xp = (sigma1*tau1)/k11
XpK = (k7*k9*k10*tau2)/(k6*(k7*k9 + k8*sigma1 + k9*sigma1))
XpM = tau2
Xpp = tau1
XppM = (k7*k10*tau2)/(k7*k9 + k8*sigma1 + k9*sigma1)
XpsM = (k10*k12*sigma1*tau2*(k8 + k9))/(k11*(k13 + k14)*(k7*k9 + k8*sigma1 + k9*sigma1))

Solving positive steady state parametrization of the network...

The solution is:

K = (XpM*k7*k9*k10*k11*(k5 + k6))/(Xpp*k4*k6*sigma1*(k7*k9 + k8*sigma1 + k9*sigma1))
M = (XpM*k10*(k8 + k9))/(Xpp*(k7*k9 + k8*sigma1 + k9*sigma1))
X = (Xpp*sigma1*sigma2)/(k11*k16)
XK = (XpM*k10*k12*k14*sigma1*(k8 + k9))/(k3*k11*(k13 + k14)*(k7*k9 + k8*sigma1 + k9*sigma1))
XM = (XpM*k10*sigma1*(k8 + k9)*(k12*k14 + k13*sigma2 + k14*sigma2))/(k11*k15*(k13 + k14)*(k7*k9 + k8*sigma1 + k9*sig
Xp = (Xpp*sigma1)/k11
XpK = (XpM*k7*k9*k10)/(k6*(k7*k9 + k8*sigma1 + k9*sigma1))
XppM = (XpM*k7*k10)/(k7*k9 + k8*sigma1 + k9*sigma1)
XpsM = (XpM*k10*k12*sigma1*(k8 + k9))/(k11*(k13 + k14)*(k7*k9 + k8*sigma1 + k9*sigma1))
Free parameters: XpM, Xpp, sigma1, sigma2

Kinetic deficiency is positive.
Number of free parameters that can be solved: 1
Refer to the additional conditions displayed above.

Conservation laws:
dK/dt + dXK/dt + dXpK/dt = 0
-dK/dt - dM/dt + dX/dt + dXp/dt + dXpp/dt = 0
dM/dt + dXM/dt + dXpM/dt + dXppM/dt + dXpsM/dt = 0
```

Figure 7: Output of CRITERIA for the MAPK model.

The revisions are given as follows:

(Page 10, lines 378-329)

Improvements in CRITERIA over COMPILES

In this subsection, we summarize the improvements introduced in CRITERIA over COMPILES. Specifically, we incorporate three key modifications to the COMPILES framework to address its issues and limitations. First, we adopt the method developed in [5] to translate networks into becoming WR and DZ using EFMs. This was discussed in detail in a previous subsection using a simple histidine kinase example. By replacing the original translation procedure in COMPILES, we resolve a major computational bottleneck

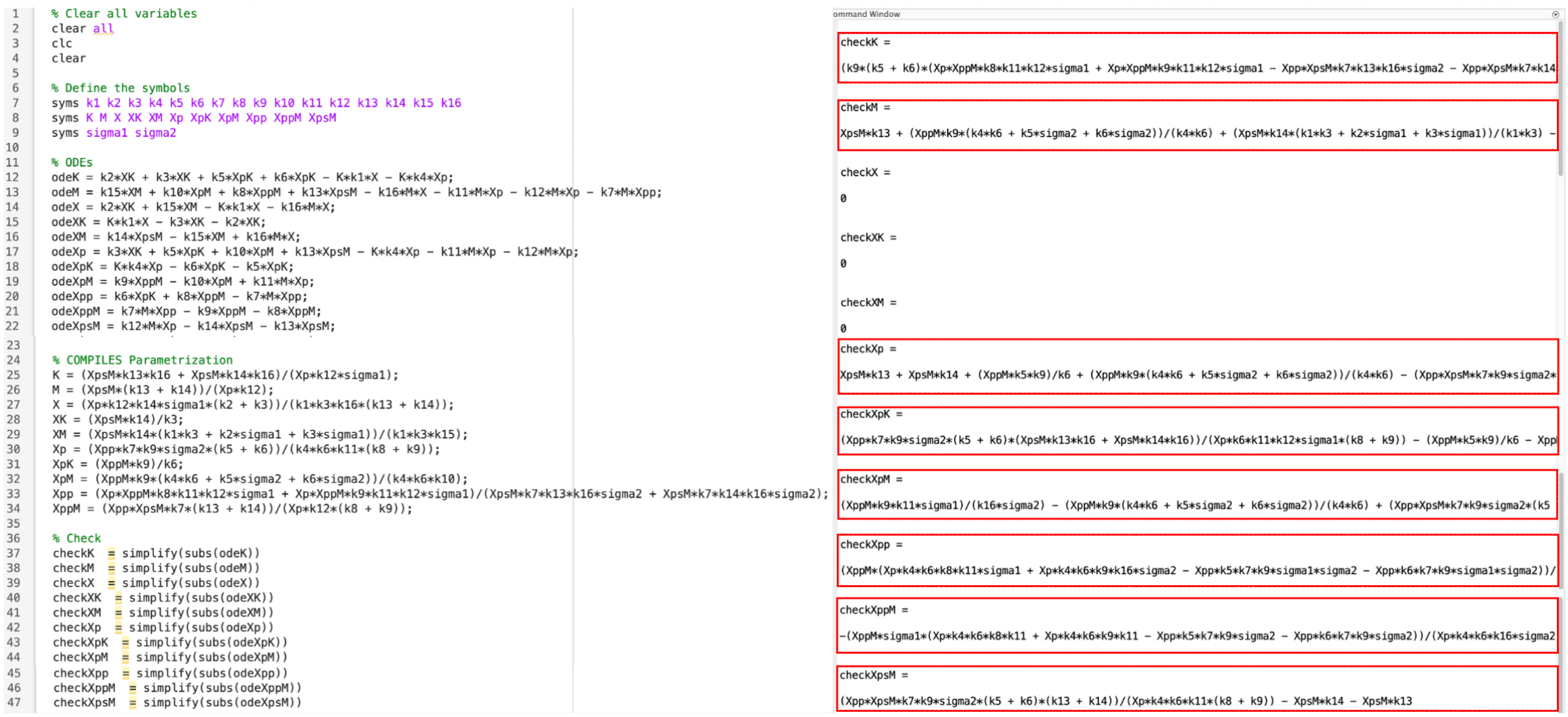

Figure 8: Checking of the parametrization obtained by COMPILES for the MAPK model.

making CRITERIA more practical for analyzing larger reaction networks.

Second, we reorder the steps of COMPILES. Instead of computing the equilibrium parametrization of each subnetwork individually, we first merge all subnetworks into a single network by taking the union of their reactions, and then compute the parametrization for the entire network. This modification is crucial for avoiding dependencies in the equilibrium expressions, as described in the Introduction section.

(Pages 10-11, lines 342-355)

Finally, we extend the applicability of the parametrization procedure to handle cases in which the deficiency of the kinetic-order CRN is positive based on the theory established in [22]. In particular, the core algorithm in COMPILES does not consider the theoretical foundations for models with positive kinetic deficiency according to Case 2 of Theorem 2 (see the Methods section). This means that COMPILES neither processes nor outputs the additional algebraic conditions necessary for the final parametrization of networks with positive kinetic deficiency. Take, for example, the MAPK model considered above. Its kinetic deficiency is equal to one. By Case 2 of Theorem 2, one additional condition needs to be imposed in the final parametrization. Applying CRITERIA, we get

$$\sigma_2 = \frac{k_4 k_6 k_{12} k_{14} k_{16} \sigma_1 (k_2 + k_3)(k_8 + k_9)}{k_1 k_3 k_7 k_9 k_{11} (k_5 + k_6)(k_{13} + k_{14})}$$

as the additional condition which is not captured by COMPILES. By considering Case 2 of Theorem 2, we get a valid final parametrization for models whose kinetic defiency is positive. In the next subsection, we compare the runtime performance of COMPILES and CRITERIA by applying them to a variety of models.

Comment 3

The key reference of this work is the authors' previous work COMPILES (Hernandez et al., PLoS Comput Biol. 2023;19(4):e1011039), while in a broader perspective, this work concerns parametrization of steady states of biochemical reaction networks, which is a fundamental problem in chemical reaction network theory. However, in Introduction, the authors only briefly mention COMPILES and do not discuss other related works in the field. I think it would be better if the authors provide a more comprehensive review of the literature on this topic. Please see, for example, the Introduction section of the paper (Bulletin of Mathematical Biology (2019) 81:1143–1172) by Johnston, Müller, and Pantea and the references therein for some examples of relevant works.

We thank the reviewer for this valuable suggestion. We agree that our original Introduction did not adequately place our work within the broader literature on steady state parametrization. In the revised manuscript, we have expanded the Introduction to include a more comprehensive review of the relevant literature. We believe these additions better position our work within the existing body of research on the topic.

We revise as follows:

(Page 2, lines 60-84)

Despite their importance in understanding biochemical systems, the mathematical study of positive equilibria remains challenging because of the high dimensionality of the underlying dynamical systems, the significant nonlinearities they exhibit, and the large number of parameters, many of which are typically unknown. Hence, recent research has focused on network-based approaches for deriving analytic positive equilibria of biochemical systems that are valid independently of specific parameter values [2-4,18,22].

Several approaches have been developed towards this goal. In [31-33], the set of steady states in mass-action systems is characterized using tools from algebraic geometry. Specifically, the authors showed that the set of positive steady states can be characterized as an algebraic variety, allowing techniques such as Gröbner bases to be used to study positive equilibria. Another line of research exploits the structure of particular classes of biochemical reaction networks. In [4,34], the rational parametrization theorem was established for a class of multisite post-translational modification systems, which states that every positive steady states of these systems admits a rational parametrization in terms of a collection of free parameters and the reaction rate constants. The authors also provided a procedure for obtaining this parametrization. More recently, a general framework was developed for explicitly constructing positive equilibrium parametrizations for broader classes of biochemical reaction networks [22]. Their approach uses deficiency theory [16], generalized mass-action systems [17], and network translation [18]. Rather than using tools from algebraic geometry, the method draws on techniques from linear algebra.

Building upon this theoretical framework, a publicly-available computational package built in MATLAB called COMPILES (COMPutIng anaLytic stEady States) was devel-

oped to facilitate this process [2].

Comments 4,5,6

a. Line 66: For consistency, the term “weak reversibility (WR)” should be revised as “being weakly reversible (WR)” as the term “deficiency zero” is used as an adjective in the same sentence.
b. Line 173: “elementary flux modes (EM) compatible” should be revised as “elementary flux modes compatible (EM-compatible)”. This is already used by the authors in Line 781. It’s a bit tricky as the term “elementary flux modes” is abbreviated as “EFMs” throughout the manuscript. I’d be happy to see any consistent, clear way to abbreviate the terms.
c. Algorithm 2 Bullet point 2: There is a minor quotation mark typo, which is common in Latex-generated PDF. The term ”active” (including the quotation marks) should be revised as “active”. There are more quotation mark typos in the manuscript, e.g., Line 246. Please check the manuscript for more quotation mark typos and fix them.

<u>Comment 4</u>

The revision is as follows: (Page 2, line 86)

being *weakly reversible* (WR)

<u>Comment 5</u>

The revision is as follows:

(Page 6, line 200)

CS-compatible

EM-compatible

(Algorithm 1)

EFMs

<u>Comment 6</u>

The revisions are as follows:

(Algorithm 2)

“active”

“processed”

(Page 25, line 719)

“sink”

(Page 25, line 720)

"source"

(Page 9, line 288)

"rate constant"

Comment 7

I suggest the authors to merge Figures 4 and 5 into a single figure with two panels, as it would be more efficient to see the similarities and differences between the two frameworks. Not just merging the two figures, but also revising the figures so that the readers can easily compare the two frameworks would be helpful.

We thank the reviewer for this comment. This is also one of the comments of the first reviewer. We have combined Figures 4 and 5 into a single figure that presents the two computational frameworks in a vertical side-by-side layout, facilitating a direct comparison between them. The revised figure is now presented as Figure 4. See Page 22 in the main manuscript.

Comment 8

Line 240: $k_3 P_1$ is the reaction rate for the reaction $G_1 + P_1 \to G_1$, which is obtained after network translation. It is correct. However, please add some explanation why that is the correct reaction rate and also refer the relevant part of Figure 1 or any other equations in the manuscript. It can be confusing to the readers who are not familiar with the concept of network translation and the theory of generalized chemical reaction networks.

We thank the reviewer for this very helpful suggestion to further improve the clarity of the manuscript. We have added some discussion to explain why the assigned reaction rate is the correct choice for the translated reaction and how it preserves the dynamics of the original reaction network. We have also added explicit references to the relevant panel of Figure 1 to better guide readers through the whole procedure. Lastly, we have revised the Generalized chemical reaction network subsection of the Methods section to provide a more detailed explanation of the underlying principles and to illustrate them using a simple toy example for added reference.

The revisions are given as follows:

(Page 9, line 267)

Refer to Fig 1b.

(Page 9, line 272-283)

To do this, we preserve the reaction rates of the original network so that the translated network remains dynamically equivalent to the original system (see Methods section under Generalized chemical reaction networks for a more detailed discussion). In particular, we assign the reaction rate k_3P_1 to the translated reaction $G_1 + P_1 \longrightarrow G_1$ because P_1 is the source complex of the corresponding reaction in the original network. In this manner, we preserve the reaction kinetics of the original reaction. Moreover, the reaction vector of the translated reaction is given by

$$(G_1) - (G_1 + P_1) = -P_1 = [0\ \ 0\ \ -1\ \ 0\ \ 0\ \ 0\ \ 0]^T,$$

which is precisely the third column in the stoichiometric matrix of the original network (see Eq (1)). These guarantee that the translated network generates the same dynamical system as the original network. Otherwise, the translated network would no longer be dynamically equivalent to the original network, and the subsequent steady state analysis would not correspond to the original biochemical system.

(Page 9, line 296-297)

This yields a translated network that is both WR and DZ as shown in Fig 1b.

(Page 28, lined 829-831)

We can do this by allowing the kinetic complex to determine the reaction rate while having the stoichiometric complex determine the reaction vector. Doing this for all reactions, we are assured to get the same ODE system since the original reaction rates and stoichiometric matrix are preserved.

(Page 29, lines 870-877)

Determining the reaction rates of the GCRN, we have

$$R_1 : k_1ab \quad R_2 : k_2a \quad R_3 : k_3b \quad R_4 : k_4c \quad R_5 : k_5.$$

Moreover, the reaction vectors of the GCRN are the following:

$$\begin{aligned}
&R_1 : (B + C) - (A + B) = C - A = [-1\ \ 0\ \ 1]^T \\
&R_2 : (B) - (A) = B - A = [-1\ \ 1\ \ 0]^T \\
&R_3 : (A) - (B) = A - B = [1\ \ -1\ \ 0]^T \\
&R_4 : (A + B) - (A + B + C) = -C = [0\ \ 0\ \ -1]^T \\
&R_5 : (A + B + C) - (B + C) = A = [1\ \ 0\ \ 0]^T
\end{aligned}$$

It can be observed that the reaction vectors we have computed coincide with those obtained in Example 1, while the reaction rates agree exactly with those derived in Example 4. Hence, the original CRN and the GCRN in this example are dynamically equivalent. Finally, it can be verified that the GCRN has zero effective deficiency ($\delta = 0$) and zero kinetic deficiency ($\tilde{\delta} = 0$), so Case 1 of Theorem 2 applies.
