# Supplementary material for: CRITERIA: A network decomposition and elementary flux mode translation-based tool for computing equilibria of biochemical systems
Source: PLoS Comput Biol. 2026 Aug 11;22(8):e1014633. doi: 10.1371/journal.pcbi.1014633 (PMC13475990; doi:10.1371/journal.pcbi.1014633)
Supplement: Peer review history

# Peer review: Decision Letter 0

**DOI:** 10.1371/journal.pcbi.1014633.r001

**Authors:** Pedro Mendes, Sylvain Soliman

17 Jun 2026

PCOMPBIOL-D-26-01008

CRITERIA: A network decomposition and elementary flux mode translation-based tool for computing equilibria of biochemical systems

PLOS Computational Biology

Dear Dr. Hernandez,

Thank you for submitting your manuscript to PLOS Computational Biology. After careful consideration, we feel that it has merit but does not fully meet PLOS Computational Biology's publication criteria as it currently stands. Therefore, we invite you to submit a revised version of the manuscript that addresses the points raised during the review process.

Please submit your revised manuscript by Aug 17 2026 11:59PM. If you will need more time than this to complete your revisions, please reply to this message or contact the journal office at ploscompbiol@plos.org. When you're ready to submit your revision, log on to https://www.editorialmanager.com/pcompbiol/ and select the 'Submissions Needing Revision' folder to locate your manuscript file.

Please include the following items when submitting your revised manuscript:

* A letter that responds to each point raised by the editor and reviewer(s). You should upload this letter as a separate file labeled 'Response to Reviewers'. This file does not need to include responses to formatting updates and technical items listed in the 'Journal Requirements' section below.

* A marked-up copy of your manuscript that highlights changes made to the original version. You should upload this as a separate file labeled 'Revised Manuscript with Track Changes'.

* An unmarked version of your revised paper without tracked changes. You should upload this as a separate file labeled 'Manuscript'.

If you would like to make changes to your financial disclosure, competing interests statement, or data availability statement, please make these updates within the submission form at the time of resubmission. Guidelines for resubmitting your figure files are available below the reviewer comments at the end of this letter.

As the corresponding author, your ORCID iD is verified in the submission system and will appear in the published article. PLOS supports the use of ORCID, and we encourage all coauthors to register for an ORCID iD and use it as well. Please encourage your coauthors to verify their ORCID iD within the submission system before final acceptance, as unverified ORCID iDs will not appear in the published article. Only the individual author can complete the verification step; PLOS staff cannot verify ORCID iDs on behalf of authors.

We look forward to receiving your revised manuscript.

Kind regards,

Sylvain Soliman

Academic Editor

PLOS Computational Biology

Pedro Mendes

Section Editor

PLOS Computational Biology

**Additional Editor Comments (if provided):**

**Journal Requirements:**

If the reviewer comments include a recommendation to cite specific previously published works, please review and evaluate these publications to determine whether they are relevant and should be cited. There is no requirement to cite these works unless the editor has indicated otherwise.

1)<carina-action-element class="ng-star-inserted">Update of 'Manuscript' file type requested</carina-action-element>

<carina-action-element class="ng-star-inserted">We ask that a manuscript source file is provided at Revision. Please upload your manuscript file as a .doc, .docx, .rtf or .tex. If you are providing a .tex file, please upload it under the item type u2018LaTeX Source Fileu2019 and leave your .pdf version as the item type u2018Manuscriptu2019.</carina-action-element>

2)<carina-action-element class="ng-star-inserted">Figures - embedded</carina-action-element>

<carina-action-element class="ng-star-inserted">Please upload all main figures as separate Figure files in .tif or .eps format. For more information about how to convert and format your figure files please see our guidelines: </carina-action-element>

<carina-action-element class="ng-star-inserted">https://journals.plos.org/</carina-action-element><carina-action-element class="ng-star-inserted">ploscompbiol</carina-action-element><carina-action-element class="ng-star-inserted">/s/figures</carina-action-element>

3)<carina-action-element class="ng-star-inserted">Citation missing figure</carina-action-element>

<carina-action-element class="ng-star-inserted">Please upload a copy of </carina-action-element><carina-action-element class="ng-star-inserted">Figures</carina-action-element><carina-action-element class="ng-star-inserted"> </carina-action-element><carina-action-element class="ng-star-inserted">1A-D.2, 3A-C, 4, and 5</carina-action-element><carina-action-element class="ng-star-inserted"> which you refer to in your text on </carina-action-element><carina-action-element class="ng-star-inserted">pages</carina-action-element><carina-action-element class="ng-star-inserted"> </carina-action-element><carina-action-element class="ng-star-inserted">3, 15, 16, and 18</carina-action-element><carina-action-element class="ng-star-inserted">. Or, if the figure is no longer to be included as part of the submission please remove all reference to it within the text.</carina-action-element>

4) <carina-action-element class="ng-star-inserted">Financial disclosure issues</carina-action-element>

<carina-action-element class="ng-star-inserted">Please </carina-action-element><carina-action-element class="ng-star-inserted">amend your</carina-action-element><carina-action-element class="ng-star-inserted"> detailed Financial Disclosure statement. This is published with the article. It must therefore be completed in full sentences and contain the exact wording you wish to be published.</carina-action-element>

<carina-action-element class="ng-star-inserted">1) State the initials, alongside each funding source, of each author to receive each grant. For example: "This work was supported by the National Institutes of Health (####### to AM; ###### to CJ) and the National Science Foundation (###### to AM)." 2) State what role the funders took in the study. If the funders had no role in your study, please state: "The funders had no role in study design, data collection and analysis, decision to publish, or preparation of the manuscript." 3) If any authors received a salary from any of your funders, please state which authors and which funders..</carina-action-element>

<carina-action-element class="ng-star-inserted">If you did not receive any funding for this study, please simply state: u201cThe authors received no specific funding for this work.u201d</carina-action-element>

<carina-action-element class="ng-star-inserted"></carina-action-element>

**Reviewers' comments:**

Reviewer's Responses to Questions

**Comments to the Authors:**

**Please note here if the review is uploaded as an attachment.**

Reviewer #1: Review is uploaded as an attachment.

Reviewer #2: This is a referee report for "CRITERIA: A network decomposition and elementary flux mode translation-based tool for computing equilibria of biochemical systems" by Exequiel Jun V. Villejo, Aurelio A. de los Reyes V, Bryan S. Hernandez (PCOMPBIOL-D-26-01008).

This paper addresses the problem of computing steady state parametrizations for mass-action systems. In previous work, the authors utilized the network-based process of network translation to construct a MATLAB program called COMPILES. This work extends that program in a new direction by utilizing an elementary-flux-mode-based algorithm for determining network translations. The authors also swap the order of steady state determination and network recombination, leaving the algebraic step of steady state determination for the final step. The authors present a MATLAB program called CRITERIA which includes modules for both the translation and steady state parametrization portions of this workflow. The authors present documentation and a number of interesting worked out examples, including the EnvZ-OmpR signaling pathway and CRISPRi toggle switch.

The technical details utilized are mathematically sound and the program works incredibly well on all of the examples I checked. The program utilizes two recent theoretical advances which have not previously been implemented computationally in a widespread manner: Johnston and Burton's (2019) elementary flux mode approach to network translation, and Johnston, Muller, and Pantea's (2019) rational parametrization approach to weakly reversible networks. Implementing these theoretical formulas into a single computational package is highly non-trivial as it involves binary programming, convex polyhedral geometry, generalized inverses, and algebraic manipulation, among other steps. The computational test cases and worked-out examples presented in the paper are appropriate.

I expect that, once widely known, this program will be of profound benefit to the chemical reaction network and systems biology communities.

Suggestions/notes:

Line 11: "Analytically deriving equilibria, sometimes referred to as steady states, which often..." Remove "sometimes referred to as steady states"

Lines 55-56: "... since they characterize the long-term behavior of these systems." They "sometimes characterize" or "often characterize" the long-term behavior (limit cycles, chaos, and unbounded solutions are possible too)

Equation between lines 101-102: Perhaps a better example could be used here? I am not sure what the problem is here since XpY in the second equation could be substituted into the first equation and then you just have XD=0 or some equation for sigma1. If you had XD in the denominator or something like that, you would end up with a quadratic and the point might be more clear. That said, it seems like you are drawing from a real example, but I might suggest picking a more uncomfortable example than the one presented.

Line 61: "Recent research has focused on network-based approaches in DERIVERING analytic..." DELIVERING

Lines 97-98: "Second, there is a merging issue in COMPILES resulting TO interdependencies in the derived equilibrium parametrization." resulting in

Lines 163-164: "Several variations of the algorithm EXISTS." exist

Lines 241-242: "... while assigning to each of the source NODE of the edges..." nodes

Line 284: "It is WOTH noting here that all benchmark models are..." WORTH noting

Figure 1: "... the complex in parentheses is the kinetic complex associated IN that node." with

Line 324: "Now, it can be observed that..." "Now" is too colloquial for a journal paper. I would suggest simply "It can be observed that..."

Equation between lines 369 and 370: It is interesting and nonstandard that you allow conservation laws with negative coefficients. This also seems to be a feature of the computational package. This is particular interesting because there is an obvious second conservation law with positive coefficient for this example (Y+Yp+XpY+XDYp+XTYp=constant). Is it guaranteed that this will not lead to problems for positivity of the final parametrization? Is this just for show? At the very least, the possibility of conservation laws with negative coefficients should be noted in the paper.

Example in paragraph bound by lines 382 and 389: It is striking that you are able to show multistationarity as well as absolute concentration robustness for this example. To the best of my knowledge, this is the first time this has been shown for this model. This is very interesting!

General note on elementary flux modes: It should be noted that the elementary flux mode approach will only work if the coefficients of all the reactions in every elementary flux mode can be scaled to one. For example, the method will not work if one of the elementary flux modes has coefficients (1,2,1)---that is, one firing of the first reaction, two of the second, and one of the third are required to obtain stoichiometric balance. Since this limitation inherited from the underlying Johnston and Burton paper will be a limitation here as well, it should be mentioned.

Lines 433-434: "ACR suggests an intrinsic buffering mechanism wherein the EQULIBRIUM concentration of dCas9 remains..." equilibrium

Line 454: "... (see Fig 5 and its ACCOMPANIED discussion)." accompanying

Figure 4: "... thereby simplifying the analysis by focusing on smaller individual pieces rather than looking the network as a whole." rather than looking AT

Line 687: "In this subsection, we formally define the concept of a GCRN, which IS pioneered by..." was

Note on CRITERIA: The CRITERIA program works very well in the examples I checked. There are two notes I would like to make:

(1) It would be very nice if a user-friendly interface for implementing networks (like CRNTool or CoNtRol) could be developed. That said, the program is very well documented and I have no problem digging into the prebuilt MATLAB programs to change networks.

(2) For higher kinetic deficiency systems, the program does not seem to identify parameters generated from phantom edges (i.e., the sigmas) as free parameters. For example, when I run ModelL.m, it gives a parametrization including the parameter sigma1 but only says "Free parameters: XpY, Y".

Reviewer #3: Please refer to the attached PDF file for review comments.

**********

**Have the authors made all data and (if applicable) computational code underlying the findings in their manuscript fully available?**

The PLOS Data policy requires authors to make all data and code underlying the findings described in their manuscript fully available without restriction, with rare exception (please refer to the Data Availability Statement in the manuscript PDF file). The data and code should be provided as part of the manuscript or its supporting information, or deposited to a public repository. For example, in addition to summary statistics, the data points behind means, medians and variance measures should be available. If there are restrictions on publicly sharing data or code —e.g. participant privacy or use of data from a third party—those must be specified.

Reviewer #1: Yes

Reviewer #2: Yes

Reviewer #3: Yes

**********

PLOS authors have the option to publish the peer review history of their article (what does this mean?). If published, this will include your full peer review and any attached files.

If you choose “no”, your identity will remain anonymous but your review may still be made public.

**Do you want your identity to be public for this peer review?**  For information about this choice, including consent withdrawal, please see our Privacy Policy.

Reviewer #1: No

Reviewer #2: No

Reviewer #3: No

[NOTE: If reviewer comments were submitted as an attachment file, they will be attached to this email and accessible via the submission site. Please log into your account, locate the manuscript record, and check for the action link "View Attachments". If this link does not appear, there are no attachment files.]

**Figure resubmission:**

While revising your submission, we strongly recommend that you use PLOS’s NAAS tool (https://ngplosjournals.pagemajik.ai/artanalysis) to test your figure files. NAAS can convert your figure files to the TIFF file type and meet basic requirements (such as print size, resolution), or provide you with a report on issues that do not meet our requirements and that NAAS cannot fix.

After uploading your figures to PLOS’s NAAS tool - https://ngplosjournals.pagemajik.ai/artanalysis, NAAS will process the files provided and display the results in the "Uploaded Files" section of the page as the processing is complete. If the uploaded figures meet our requirements (or NAAS is able to fix the files to meet our requirements), the figure will be marked as "fixed" above. If NAAS is unable to fix the files, a red "failed" label will appear above. When NAAS has confirmed that the figure files meet our requirements, please download the file via the download option, and include these NAAS processed figure files when submitting your revised manuscript.

**Reproducibility:**

To enhance the reproducibility of your results, we recommend that authors of applicable studies deposit laboratory protocols in protocols.io, where a protocol can be assigned its own identifier (DOI) such that it can be cited independently in the future. Additionally, PLOS ONE offers an option to publish peer-reviewed clinical study protocols. Read more information on sharing protocols at https://plos.org/protocols?utm_medium=editorial-email&utm_source=authorletters&utm_campaign=protocols

Attachment Submitted filename: PLOS review on CRITERIA paper.pdf

Attachment Submitted filename: PCOMPBIOL-D-26-01008-review.pdf

# Author comment: Author response to Decision Letter 1

**DOI:** 10.1371/journal.pcbi.1014633.r002

8 Jul 2026

Attachment Submitted filename: Response to Reviewers.pdf

# Peer review: Decision Letter 1

**DOI:** 10.1371/journal.pcbi.1014633.r003

**Authors:** Pedro Mendes, Sylvain Soliman

26 Jul 2026

Dear Dr. Hernandez,

We are pleased to inform you that your manuscript 'CRITERIA: A network decomposition and elementary flux mode translation-based tool for computing equilibria of biochemical systems' has been provisionally accepted for publication in PLOS Computational Biology.

Before your manuscript can be formally accepted you will need to complete some formatting changes, which you will receive in a follow up email. A member of our team will be in touch with a set of requests.

Please note that your manuscript will not be scheduled for publication until you have made the required changes, so a swift response is appreciated.

IMPORTANT: The editorial review process is now complete. PLOS will only permit corrections to spelling, formatting or significant scientific errors from this point onwards. Requests for major changes, or any which affect the scientific understanding of your work, will cause delays to the publication date of your manuscript.

Should you, your institution's press office or the journal office choose to press release your paper, you will automatically be opted out of early publication. We ask that you notify us now if you or your institution is planning to press release the article. All press must be co-ordinated with PLOS.

Thank you again for supporting Open Access publishing; we are looking forward to publishing your work in PLOS Computational Biology.

Best regards,

Sylvain Soliman

Academic Editor

PLOS Computational Biology

Pedro Mendes

Section Editor

PLOS Computational Biology

***********************************************************

Please take into account the remaining minor changes for the final version.

Reviewer's Responses to Questions

**Comments to the Authors:**

**Please note here if the review is uploaded as an attachment.**

Reviewer #1: Review is uploaded as an attachment.

Reviewer #2: The suggested edits have been made to my satisfaction.

Reviewer #3: The authors have adequately addressed all comments.

**********

**Have the authors made all data and (if applicable) computational code underlying the findings in their manuscript fully available?**

The PLOS Data policy requires authors to make all data and code underlying the findings described in their manuscript fully available without restriction, with rare exception (please refer to the Data Availability Statement in the manuscript PDF file). The data and code should be provided as part of the manuscript or its supporting information, or deposited to a public repository. For example, in addition to summary statistics, the data points behind means, medians and variance measures should be available. If there are restrictions on publicly sharing data or code —e.g. participant privacy or use of data from a third party—those must be specified.

Reviewer #1: Yes

Reviewer #2: Yes

Reviewer #3: Yes

**********

PLOS authors have the option to publish the peer review history of their article (what does this mean?). If published, this will include your full peer review and any attached files.

If you choose “no”, your identity will remain anonymous but your review may still be made public.

**Do you want your identity to be public for this peer review?**  For information about this choice, including consent withdrawal, please see our Privacy Policy.

Reviewer #1: No

Reviewer #2: No

Reviewer #3: No

Attachment Submitted filename: PLOS review for revised CRITERIA paper.pdf

# Editor report: Acceptance letter

**DOI:** 10.1371/journal.pcbi.1014633.r004

**Authors:** Pedro Mendes, Sylvain Soliman

PCOMPBIOL-D-26-01008R1

CRITERIA: A network decomposition and elementary flux mode translation-based tool for computing equilibria of biochemical systems

Dear Dr Hernandez,

I am pleased to inform you that your manuscript has been formally accepted for publication in PLOS Computational Biology. Your manuscript is now with our production department and you will be notified of the publication date in due course.

The corresponding author will soon be receiving a typeset proof for review, to ensure errors have not been introduced during production. Please review the PDF proof of your manuscript carefully, as this is the last chance to correct any errors. Please note that major changes, or those which affect the scientific understanding of the work, will likely cause delays to the publication date of your manuscript.

Soon after your final files are uploaded, unless you have opted out, the early version of your manuscript will be published online. The date of the early version will be your article's publication date. The final article will be published to the same URL, and all versions of the paper will be accessible to readers.

For Research, Software, and Methods articles, you will receive an invoice from PLOS for your publication fee after your manuscript has reached the completed accept phase. If you receive an email requesting payment before acceptance or for any other service, this may be a phishing scheme. Learn how to identify phishing emails and protect your accounts at https://explore.plos.org/phishing.

Thank you again for supporting PLOS Computational Biology and open-access publishing. We are looking forward to publishing your work!

With kind regards,

Janani Seenivasan

PLOS Computational Biology | Carlyle House, Carlyle Road, Cambridge CB4 3DN | United Kingdom ploscompbiol@plos.org | Phone +44 (0) 1223-442824 | ploscompbiol.org | @PLOSCompBiol
